# Diverse bacterial pattern recognition receptors sense the core phage proteome

**DOI:** 10.1038/s41586-026-10852-6

**Published:** 2026-07-29

**Authors:** Hyunbin Lee, Sofia Luengo-Woods, Jianxiu Zhang, Kira S. Makarova, Yuri I. Wolf, Collin Chiu, Simone A. Evans, Junyi Chen, Haopeng Xiao, Liang Feng, Eugene V. Koonin, Alex Gao

**Affiliations:** 1 Department of Biochemistry, Stanford University, Stanford, CA USA; 2 Department of Microbiology and Immunology, Stanford University, Stanford, CA USA; 3 Department of Molecular and Cellular Physiology, Stanford University, Stanford, CA USA; 4 Computational Biology Branch, Division of Intramural Research, National Library of Medicine, National Institutes of Health, Bethesda, MD USA; 5 Department of Genetics, Stanford University, Stanford, CA USA; 6 Stanford Cancer Institute, Stanford University, Stanford, CA USA

**Keywords:** Molecular biology, Microbiology, Cryoelectron microscopy

## Abstract

Recognition of foreign molecules inside cells is critical for immunity across all domains of life. Proteins of the STAND NTPase superfamily^1,2^, including eukaryotic NOD-like receptors, play a central role in this process^3,4^. In bacteria and archaea, although several STAND families sense phage proteins^5–9^, their functional diversity remains largely unexplored. Here we conduct a systematic phylogenetic analysis of prokaryotic STAND NTPases and identify at least 90 structurally distinct families associated with antiviral defence. We first show that the uncharacterized Avs7 family recognizes the major capsid protein (MCP) of tailed phages. Three cryogenic electron microscopy structures of *Salmonella enterica* Avs7 reveal an asymmetric, butterfly-shaped tetramer that assembles stepwise through large, MCP-induced conformational changes, incorporating bacterial elongation factor Tu (EF-Tu) as a structural component that enhances defence. Using genetic screens with a library of 687 phage genes, we further show that 13 additional STAND families sense 13 conserved phage proteins, encompassing most of the core structural and replicative components of tailed phages. These include 2 distinct MCP-sensing families (Avs8 and Avs10) and 11 others (Avs11–21), which recognize the portal, portal adaptor, tail nozzle, head–tail connector, tail terminator, tail tube protein, tail assembly chaperone, tape measure protein, DNA polymerase, helicase/RecA-type ATPase and single-stranded DNA annealing protein, respectively. Together, our findings reveal a mechanism of host-factor repurposing and establish structure-based pattern recognition as a fundamental strategy of bacterial immunity.

## Abstract (web summary)

Systematic analysis of prokaryotic STAND NTPases — relatives of animal and plant immune receptors — uncovers diverse antiviral sensors that detect most of the core structural and replicative proteins of bacteriophages.

## Main

Intracellular surveillance for foreign molecules is a cornerstone of host defence against pathogens. In animals and plants, a major part of this process is orchestrated by NLR (NOD-like receptor) proteins, which belong to the STAND (signal transduction ATPases with numerous domains) NTPase superfamily. STANDs typically have a tripartite architecture consisting of a central NTPase domain, a C-terminal sensor and an N-terminal effector^1,2^. Immune-related members of this superfamily, including NLRs, sense pathogen-associated molecular patterns and oligomerize to initiate inflammation or programmed cell death^3^. Mammalian NLRs include NAIP, which detects bacterial flagellin and type III secretion systems^10,11^, and NOD1 and 2, which recognize peptidoglycan fragments of bacterial cell walls^12,13^.

STAND NTPases are also abundant in bacteria and archaea^1,2^, and some of these, dubbed antiviral STANDs (Avs), provide protection against phage infection^4^. We previously demonstrated that four families, Avs1–4, recognize the folds of two conserved phage proteins, the large terminase subunit and portal, through direct binding^5^. Recent studies have revealed other STAND activation mechanisms, including recognition of many proteins by a single sensor^6,7^, a jumbophage protein^9^ and host chaperone perturbation^8^. Several more STANDs have been reported to confer anti-phage protection^4,14–17^, and one branch—the NACHT clade—has been systematically analysed in the phylogenetic context^16^, but, on the whole, the vast repertoire of prokaryotic STANDs remains poorly characterized. Here, we combine large-scale genome mining with structural, biochemical and genetic analyses to define the landscape of prokaryotic defence-associated STAND proteins and reveal mechanistic principles of their activation.

## Phylogeny of prokaryotic defence-associated STANDs

**Fig. 1:**
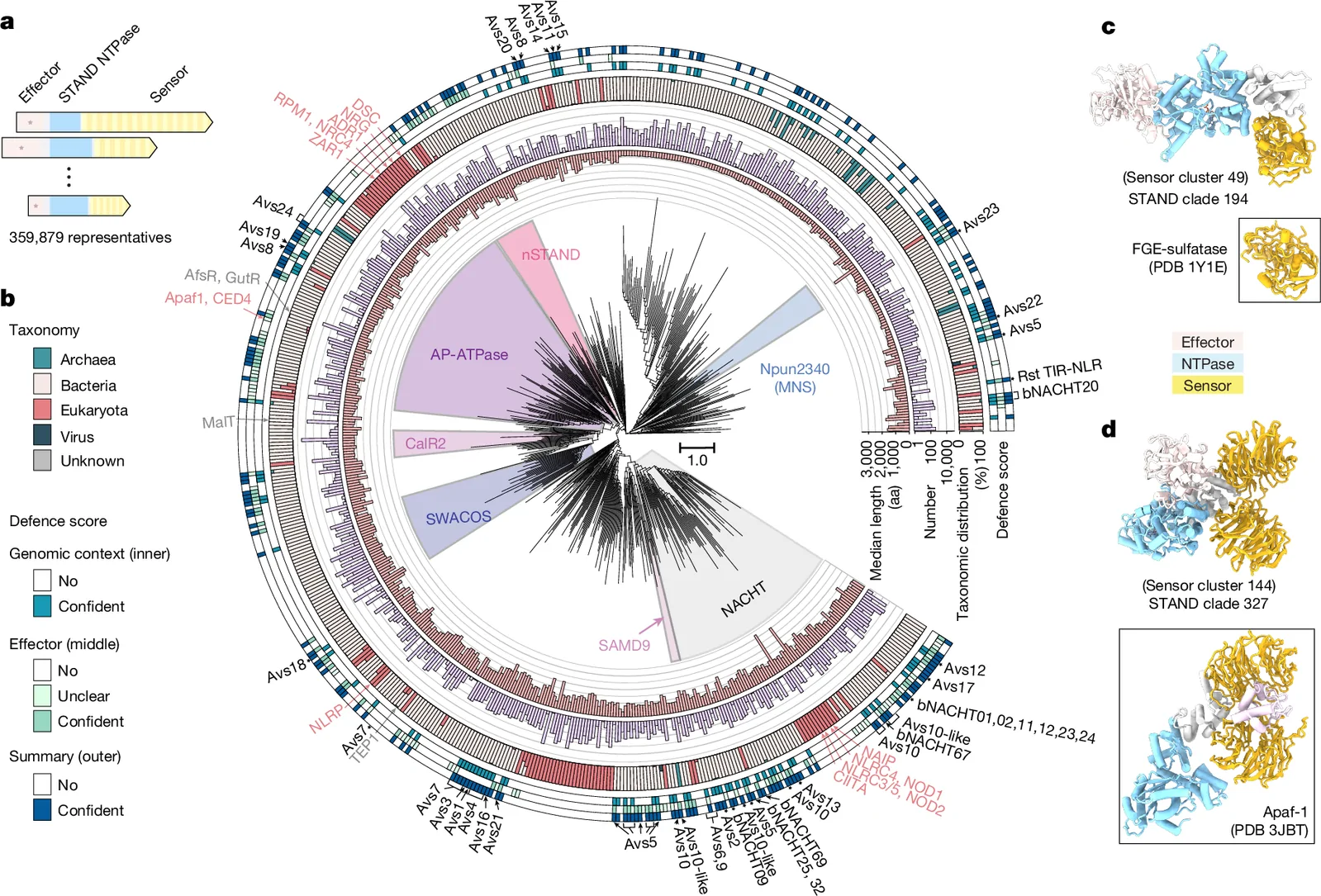
Phylogeny of defence-associated STAND NTPases in prokaryotes. **a**, Representative domain architectures of STAND NTPases. **b**, Maximum likelihood tree of the NTPase domains of 589 clades, comprising 359,879 representative sequences (Methods). MNS, NACHT, SWACOS, CalR2, AP-ATPase and nSTAND refer to previously defined clades. Eukaryotic STANDs are annotated in red. Defence scores were assigned on the basis of two criteria: the frequency of known defence genes in the genomic neighbourhood of STAND genes (genomic context) and the proportion of STANDs carrying immune-related effector domains (effector; Supplementary Table 3). Clades were classified as confident defence-associated (black) if they satisfied either criterion (Methods). Non-defence proteins are shown in grey. **c**,**d**, Representative AlphaFold3 structural models of representatives with FGE-sulfatase (**c**) or Apaf1-like WD40 β-propellers (**d**) as sensors. Numbers in parentheses represent structural clusters (1–684). aa, amino acids; Apaf-1, apoptotic protease-activating factor 1; bNACHT, bacterial NACHT; FGE, formylglycine-generating enzyme; SAMD9, sterile α-motif domain-containing 9.

**Extended Data Fig. 1:**
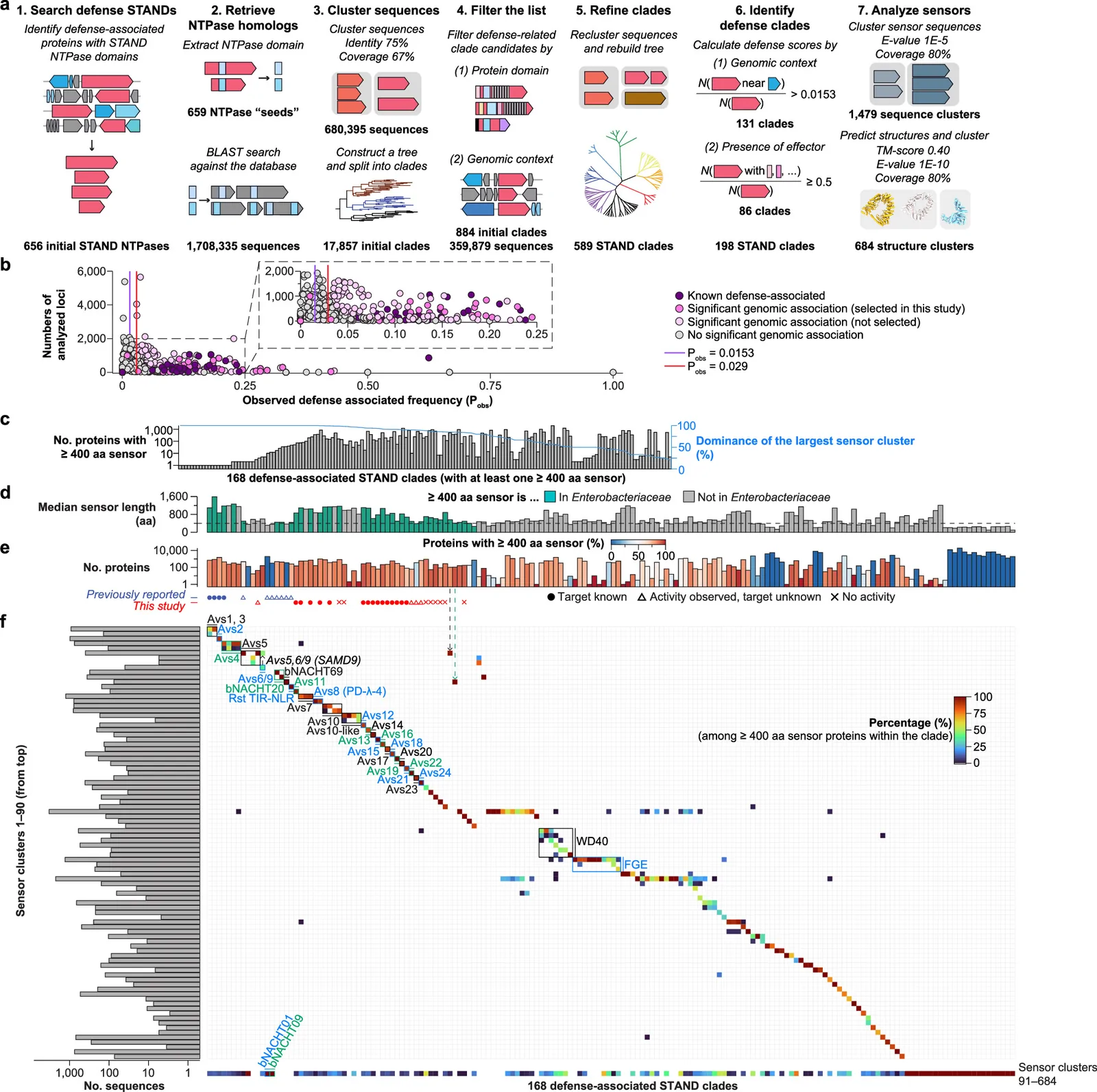
Identification of prokaryotic defence-associated STANDs and their sensors. (**a**) Schematic of the workflow to identify, analyze, and classify STAND NTPases. (**b**) Statistics of defense context enrichment for the 589 identified STAND NTPase clades, calculated using a one-sided binomial distribution (see Methods). (**c**) Number of proteins with ≥400 aa sensors per defense-associated STAND clade, alongside the percent dominance of the largest sensor cluster within each clade. (**d**) Median sensor length and (**e**) total protein count per selected STAND clade. (**f**) Number of proteins for each of the 90 selected structural clusters (left) and their distribution across STAND clades (right). STAND clades lacking sensors ≥400 aa were excluded, and percentages were calculated using sensors ≥400 aa. FGE, formylglycine-generating enzyme.

To investigate the diversity of the highly divergent defence-associated STANDs, we used a two-step, systematic approach (Fig. 1a and Extended Data Fig. 1a). We first identified a set of 656 ‘seed’ STAND proteins, including known immune-related STANDs^2,4,16–18^ and further candidates detected by their localization in genomic proximity to other defence-related genes^4,19,20^ (Supplementary Table 1). We then performed relaxed PSI-BLAST searches using the conserved NTPase domains of these seeds as queries and, after several rounds of curation (Methods), expanded the set to 359,879 diverse representatives (clustered at 75% sequence identity) distributed across 589 STAND clades (Fig. 1b, Supplementary Table 2 and Methods). These sequences included known animal and plant NLRs, although many of these were not included among the seeds, supporting the sensitivity of this approach.

We classified 198 of the 589 STAND clades as defence-associated on the basis of their proximity to known defence systems and/or the presence of a defence-related effector domain (Methods, Fig. 1a, Extended Data Fig. 1a,b and Supplementary Tables 3 and 4). Our analysis identified CRISPR-associated STANDs^21^ and prophage-encoded STANDs^22^, consistent with their established roles in anti-phage defence (Supplementary Fig. 2a,b). Defence-associated clades are interspersed with non-defence STAND clades, suggestive of many functional switches throughout the evolution of the STAND superfamily (Fig. 1b). Known NLRs from animals^23^, plants^24,25^ and bacteria^4,5,15–17^ were found in distinct clades, alongside non-immune STANDs such as transcriptional regulators^26–28^, the telomerase-associated protein TEP1 and the primary cilia protein NPHP3, consistent with earlier analyses^2,29^.

To further explore the diversity of C-terminal sensor domains of the STANDs, which are usually substantially larger than typical defence proteins (Supplementary Fig. 2c), we grouped defence-associated sensors of 400 or more amino acids into 684 clusters by sequence and structural similarity (Extended Data Fig. 1c–f and Supplementary Table 5). Most defence-associated clades were dominated by a single sensor type (Extended Data Fig. 1c). Analysis of the 90 most prevalent clusters revealed contrasting evolutionary patterns among the sensors: some were clade-specific (for example, Avs2), whereas others spanned many clades (for example, sensor structural cluster 39; Fig. 1c,d, Extended Data Fig. 1f and Supplementary Figs. 3–5). Most of the sensors were composed of tetratricopeptide repeat (TPR)-like α-helical repeats (Supplementary Figs. 3, 4a and 5), although we also identified other types, including WD40 β-propeller repeats and formylglycine-generating enzyme sulfatase-like domains (Fig. 1c,d and Supplementary Fig. 4b,c). This diversity mirrors the sensor repertoire of eukaryotic NLRs^3^.

We identified specific structural similarity between several bacterial sensors and animal proteins involved in immunity. The Avs5 sensor clustered with the SAMD9 family of animal antiviral restriction factors, consistent with recent observations^30^ (Supplementary Fig. 3a). We also detected bacterial orthologues of Apaf-1, the core component of the eukaryotic apoptosome^31^, all sharing structurally similar β-propeller sensors (Fig. 1d and Supplementary Fig. 4b). Several bacterial variants encode an N-terminal caspase-like protease in place of the eukaryotic CARD domain that recruits procaspase-9, suggesting a more direct effector mechanism than that in the animal counterparts. The closest Apaf-1 homologues were found in cyanobacteria, consistent with parallels between the defence systems in multicellular bacteria and eukaryotic immunity^32^.

## A shared sensor in Avs7 and Dsr1 detects phage MCPs

**Extended Data Fig. 2:**
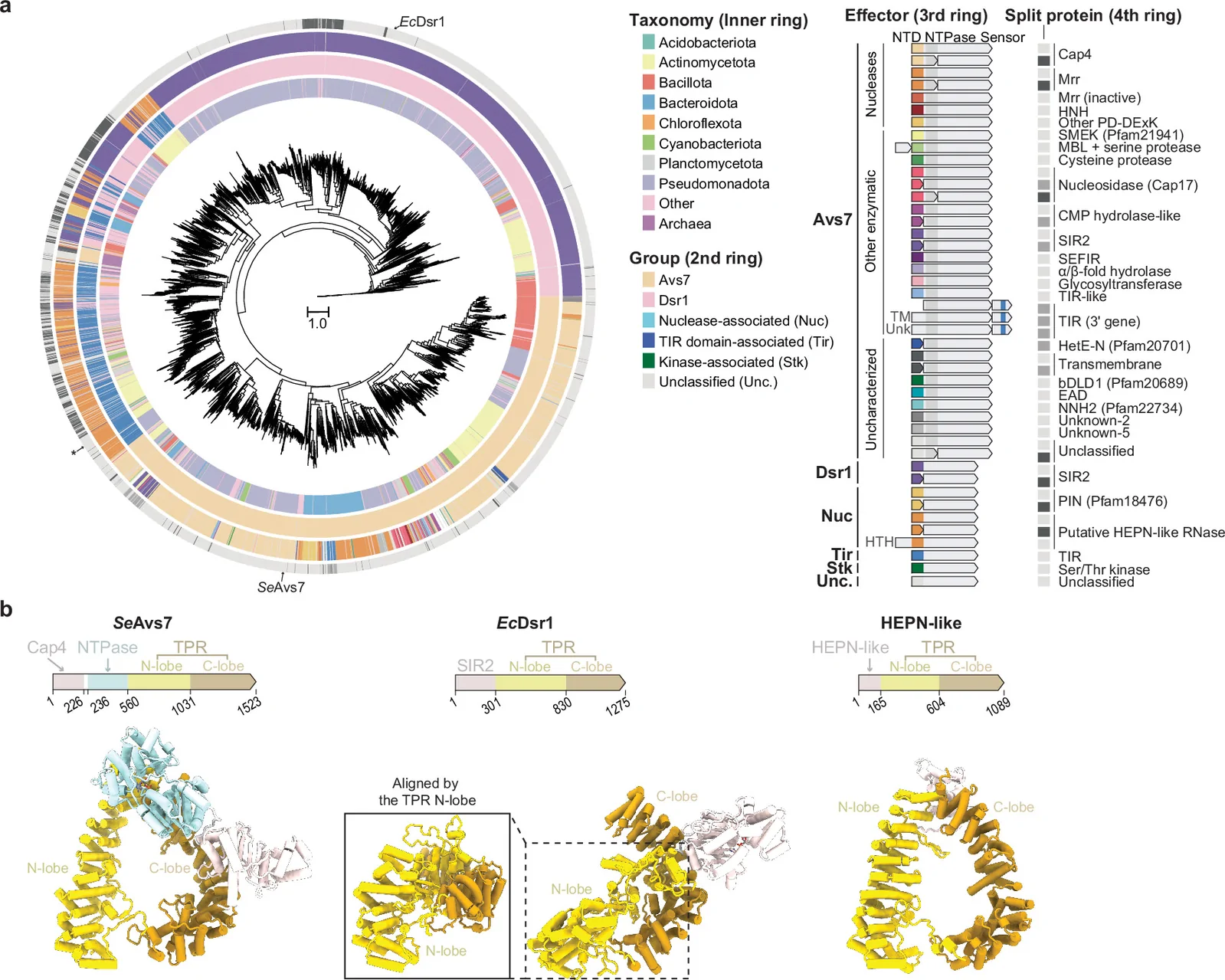
Avs7 and Dsr1 share a homologous sensor domain. (**a**) Maximum likelihood tree of the homologous sensor domains of Avs7, Dsr1, and other defense proteins (*n* = 4,969 representatives). The inner-most ring represents taxonomy, and the three outer rings denote the type and domain architecture of the respective proteins. The asterisk indicates a HEPN-like RNase-containing protein from *Streptomyces sp*. NBC_00271. Unknown-2 is also fused to several Avs2 homologs^5^. MBL, metallo-β-lactamase; EAD, effector-associated domain; TM, transmembrane; Unk, unknown. (**b**) AlphaFold3 models of *Se*Avs7, *Ec*Dsr1, and a HEPN-like domain-containing protein from *Streptomyces* sp. NBC_00271, colored by domain.

We first selected an uncharacterized STAND family, Avs7, given the consistent presence of its sensor domain across many STAND clades, its association with at least 20 distinct N-terminal effector domains and evidence of extensive horizontal gene transfer (Extended Data Fig. 1f, Supplementary Fig. 6 and Supplementary Table 6). Unexpectedly, the Avs7 sensor is shared by unrelated defence systems lacking the STAND NTPase domain, including defence-associated sirtuin 1 (Dsr1)^4^ (Extended Data Fig. 2 and Supplementary Table 8). AlphaFold3 (ref. ^33^) models of *Se*Avs7, *Escherichia coli* Dsr1 (*Ec*Dsr1) and a *Streptomyces* HEPN-containing protein revealed a conserved bilobed architecture formed by a sharply bent sensor, suggesting a common recognition mechanism (Extended Data Fig. 2b).

**Fig. 2:**
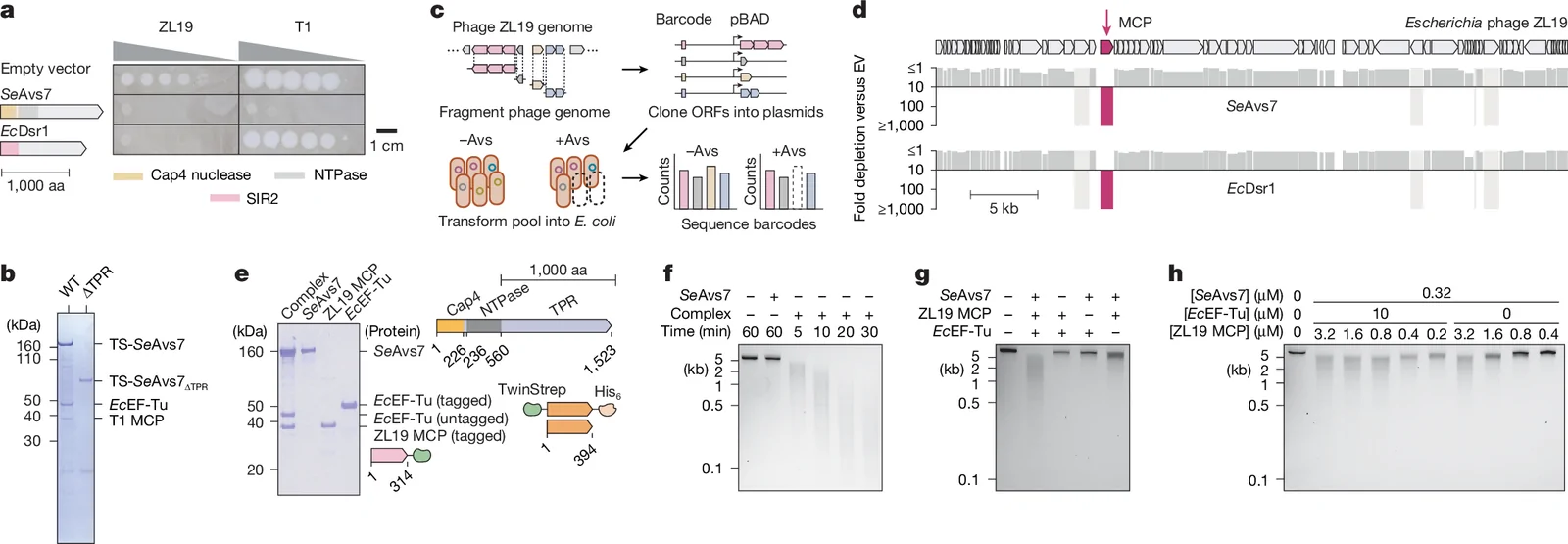
Homologous sensor domains in Avs7 and Dsr1 recognize phage MCPs. **a**, Plaque assays of *E. coli* expressing an empty vector, *Se*Avs7 or *Ec*Dsr1 against *Escherichia* phages ZL19 and T1. **b**, SDS–PAGE analysis of an *Se*Avs7 affinity pulldown. WT, wild-type; TS, TwinStrep tag; ΔTPR, Δ561–1523. **c**,**d**, Schematic of (**c**) and fold depletion from (**d**) a synthetic lethal genetic screen to identify phage triggers that induce *Se*Avs7-mediated and *Ec*Dsr1-mediated cell death. Candidate genes spanning the ZL19 genome were cloned downstream of an arabinose-inducible pBAD promoter with a unique 20-nt barcode. **e**, SDS–PAGE analysis of purified recombinant proteins and the oligomeric complex. **f**–**h**, Agarose gel analysis of in vitro nuclease activity assays with a linear dsDNA substrate: apo *Se*Avs7 versus the purified complex (**f**), the indicated combinations of *Se*Avs7, ZL19 MCP and *Ec*EF-Tu (**g**), and a titration of ZL19 MCP at a fixed concentration of *Se*Avs7, with or without *Ec*EF-Tu (**h**). See Supplementary Fig. 1 for the uncropped gel images. ORF, open reading frame; EV, empty vector. Source data

**Extended Data Fig. 3:**
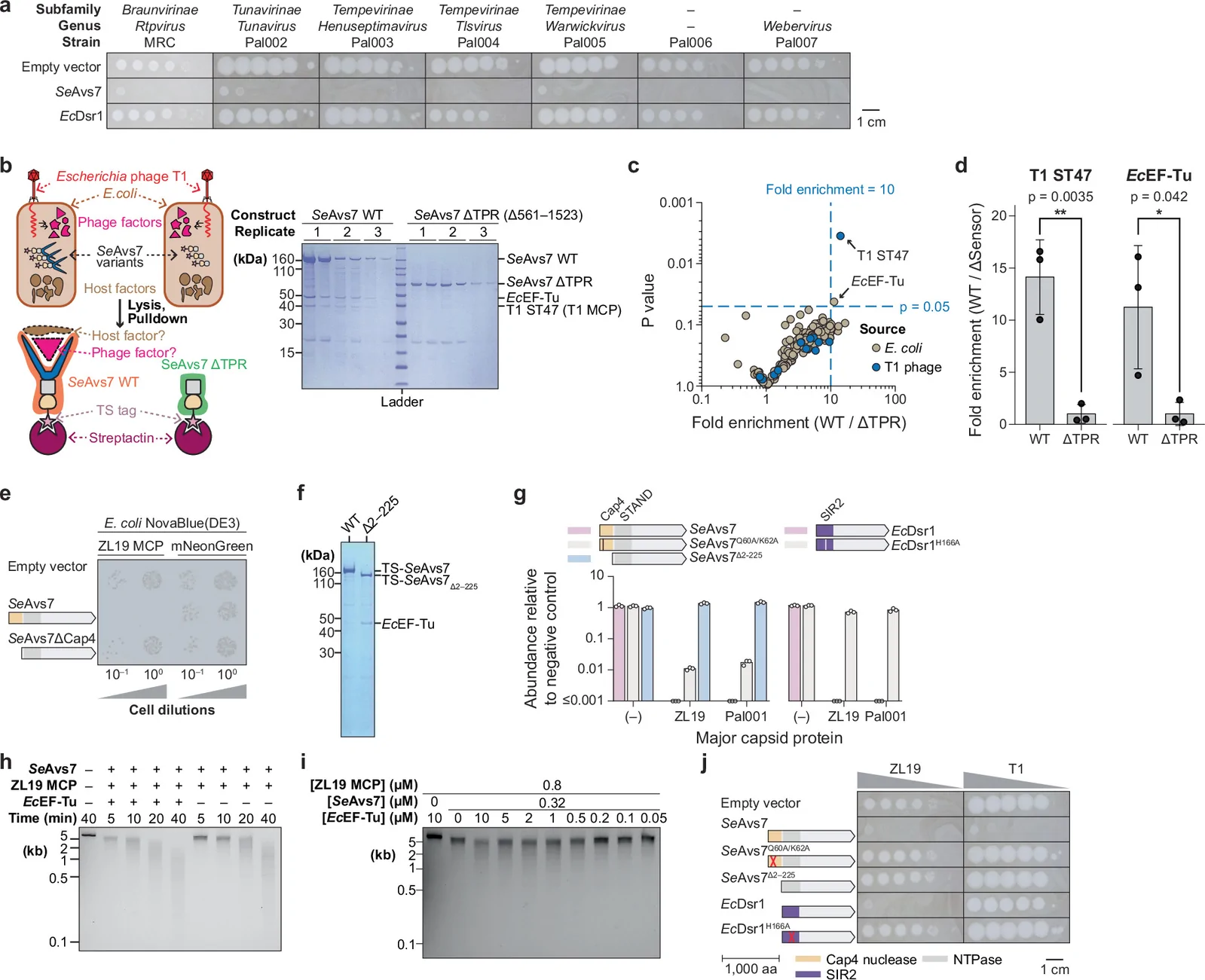
*Se*Avs7 and *Ec*Dsr1 detect phage major capsid proteins. (**a**) Plaque assays of *E. coli* expressing an empty vector, *Se*Avs7, or *Ec*Dsr1 against seven *Drexlerviridae* phages. (**b**) SDS-PAGE analysis of pulldown samples prepared for mass spectrometry, and (**c**) the corresponding volcano plot (axes in logarithmic scale). Normalized TMT signal-to-noise values from WT and ΔTPR pulldowns were compared using a two-sided, two-sample Student’s t-test (*n* = 3 replicates); *P*-values are not corrected for multiple comparisons. (**d**) Fold enrichment of the T1 major capsid protein (T1 ST47; left) and *E. coli* elongation factor Tu (*Ec*EF-Tu; right) co-purified with wild-type (WT) *Se*Avs7 compared to the *Se*Avs7 ΔSensor mutant. Bars represent the mean of the three independent biological replicates, and error bars indicate the standard deviation. Individual data points are shown. (**e**) Representative photographs of *E. coli* transformation assays. (**f**) SDS-PAGE analysis of Twin-Strep-tagged WT *Se*Avs7 and *Se*Avs7 ΔCap4 (*Se*Avs7^Δ2–225^). (**g**) Toxicity assays demonstrating that disruption of the *Se*Avs7 nuclease domain (left) or *Ec*Dsr1 NADase domain (right) abrogates cell death upon MCP co-expression. **h–i**, Agarose gel of in vitro nuclease activity assays using a linear dsDNA substrate. (**j**) Plaque assays of *E. coli* expressing the indicated *Se*Avs7 or *Ec*Dsr1 variants, challenged with *Escherichia* phages ZL19 and T1. For gel source data, see Supplementary Fig. 1. Source data

*Se*Avs7 conferred robust defence in *E. coli* against nine *Drexlerviridae* family coliphages, whereas *Ec*Dsr1 was active against only a few (Fig. 2a and Extended Data Fig. 3a). Proteomic profiling of tagged *Se*Avs7 isolated from T1-infected cells revealed significant enrichment of the T1 major capsid protein (MCP; T1 ST47) and host translation elongation factor Tu (*Ec*EF-Tu) bound to *Se*Avs7, compared with a sensor-deleted mutant (Fig. 2b, Extended Data Fig. 3b–d and Supplementary Table 9). A co-expression toxicity screen using a barcoded library of 73 genes from phage ZL19 identified the MCP as the unique activator of both *Se*Avs7-mediated and *Ec*Dsr1-mediated cell death (Fig. 2c,d and Supplementary Table 10), and direct co-expression of ZL19 MCP with *Se*Avs7 in *E. coli* caused pronounced cell death (Extended Data Fig. 3e–g). Together, these results establish MCP as the trigger for Avs7 sensors across distinct defence systems.

## The *Se*Avs7–MCP complex incorporates EF-Tu

To investigate the activation of *Se*Avs7, we recombinantly purified *Se*Avs7 and ZL19 MCP and incubated them in vitro (Supplementary Fig. 7a). Size-exclusion chromatography (SEC) demonstrated that, in the presence of ATP and Mg^2+^, these proteins formed a large multimeric complex (Fig. 2e and Supplementary Fig. 7b). This complex efficiently degraded double-stranded DNA (dsDNA) substrates, whereas *Se*Avs7 alone did not (Fig. 2f). *Ec*EF-Tu copurified with *Se*Avs7 even in the absence of phage infection and co-eluted with the *Se*Avs7–ZL19 MCP complex during SEC (Supplementary Fig. 7b). To rule out non-specific binding, we reconstituted the complex in vitro using individually purified *Se*Avs7, ZL19 MCP and *Ec*EF-Tu proteins (Fig. 2e and Supplementary Fig. 7c,d). SEC analysis confirmed that *Ec*EF-Tu was stably incorporated into the multimeric complex at a roughly 1:1:1 molar ratio, demonstrating its role as a core structural component (Supplementary Fig. 7e).

To clarify the role of each protein, we assessed nuclease activity across different combinations of the individually purified proteins (Fig. 2e and Supplementary Fig. 7c). DNA degradation strictly required both *Se*Avs7 and ZL19 MCP and was substantially enhanced by *Ec*EF-Tu (Fig. 2g). Although cleavage occurred without *Ec*EF-Tu after prolonged incubation, its presence notably increased the sensitivity of *Se*Avs7 to ZL19 MCP (Fig. 2h, Extended Data Fig. 3h,i and Supplementary Fig. 8). Given the high cellular concentration of EF-Tu (comprising 6–10% of the total *E. coli* proteome^34^) and its sequence conservation between *E. coli* and *Salmonella enterica* (a single amino acid difference), this enhancer role is probably conserved in the native host. The elimination of both defence and cellular toxicity of *Se*Avs7 by inactivation of the Cap4 nuclease indicates that the defence activity of *Se*Avs7 is not driven by EF-Tu sequestration (Extended Data Fig. 3e–g,j).

## *Se*Avs7 forms an asymmetric tetramer on MCP binding

**Fig. 3:**
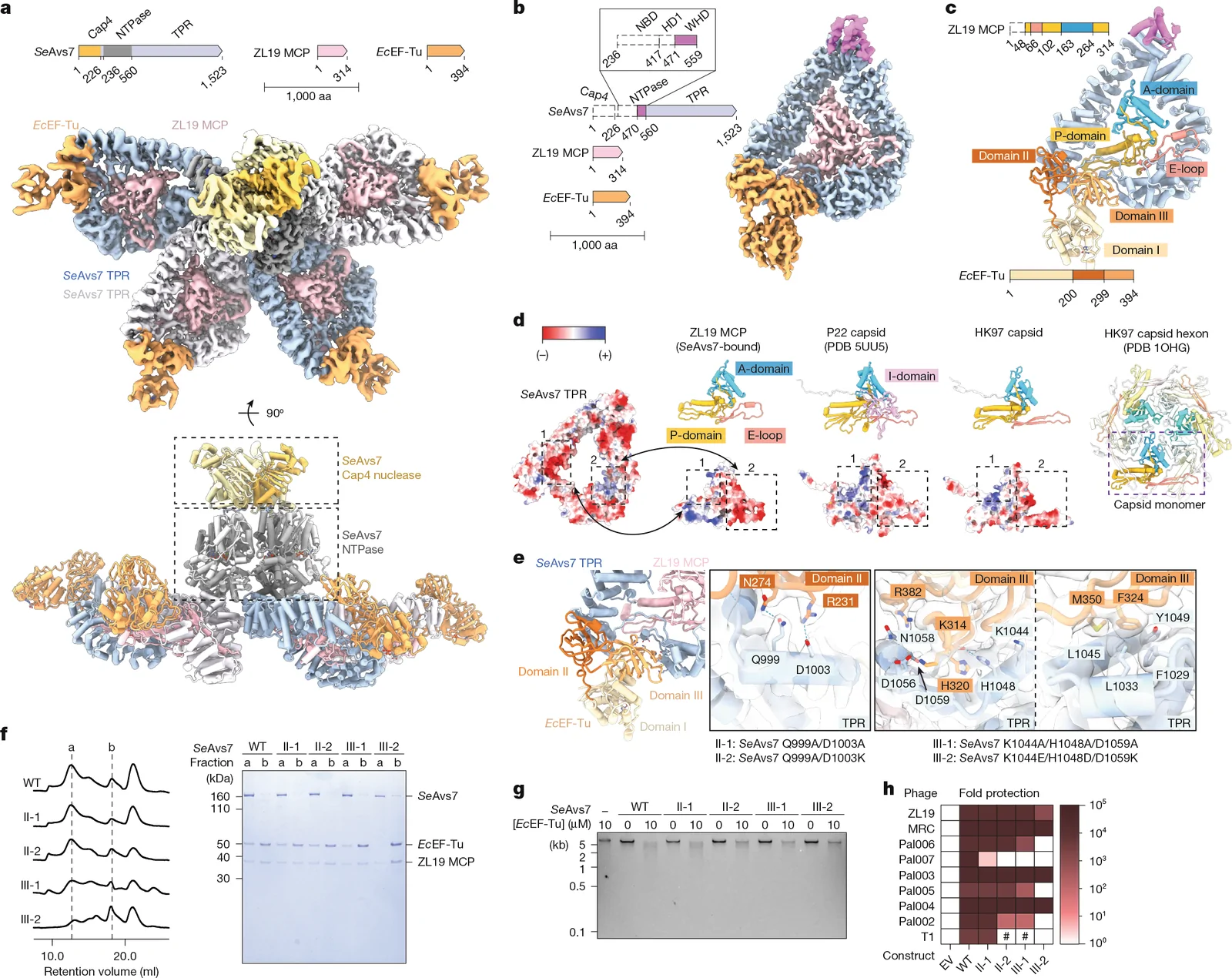
*Ec*EF-Tu enhances *Se*Avs7 complex assembly and the immune response. **a**, Composite map (top) and structure of the tetrameric *Se*Avs7–ZL19 MCP–*Ec*EF-Tu complex. **b**, Cryo-EM map of the monomeric complex. **c**, Structure of the monomeric *Se*Avs7–ZL19 MCP–*Ec*EF-Tu complex. **d**, Structural comparison of MCPs from diverse tailed phages, alongside open-book views of the electrostatic surface potentials of the MCPs and the *Se*Avs7 TPR domain. **e**, Interface between the *Se*Avs7 TPR domain and *Ec*EF-Tu domains II–III. Mutants: II-1, *Se*Avs7^Q999A/D1003A^; II-2, *Se*Avs7^Q999A/D1003K^; III-1, *Se*Avs7^K1044A/H1048A/D1059A^; III-2, *Se*Avs7^K1044E/H1048D/D1059K^. **f**, Size-exclusion chromatograms of the in vitro assembled complex and SDS–PAGE analysis of selected fractions. **g**, Agarose gel analysis of the dsDNA nuclease activity of the wild-type complex and mutants. **h**, Anti-phage activity of the wild-type and mutants against nine *Drexlerviridae* phages. The hash (#) indicates reduced plaque size without a change in the efficiency of plating. See Supplementary Fig. 1 for uncropped gel images. Source data

**Extended Data Fig. 4:**
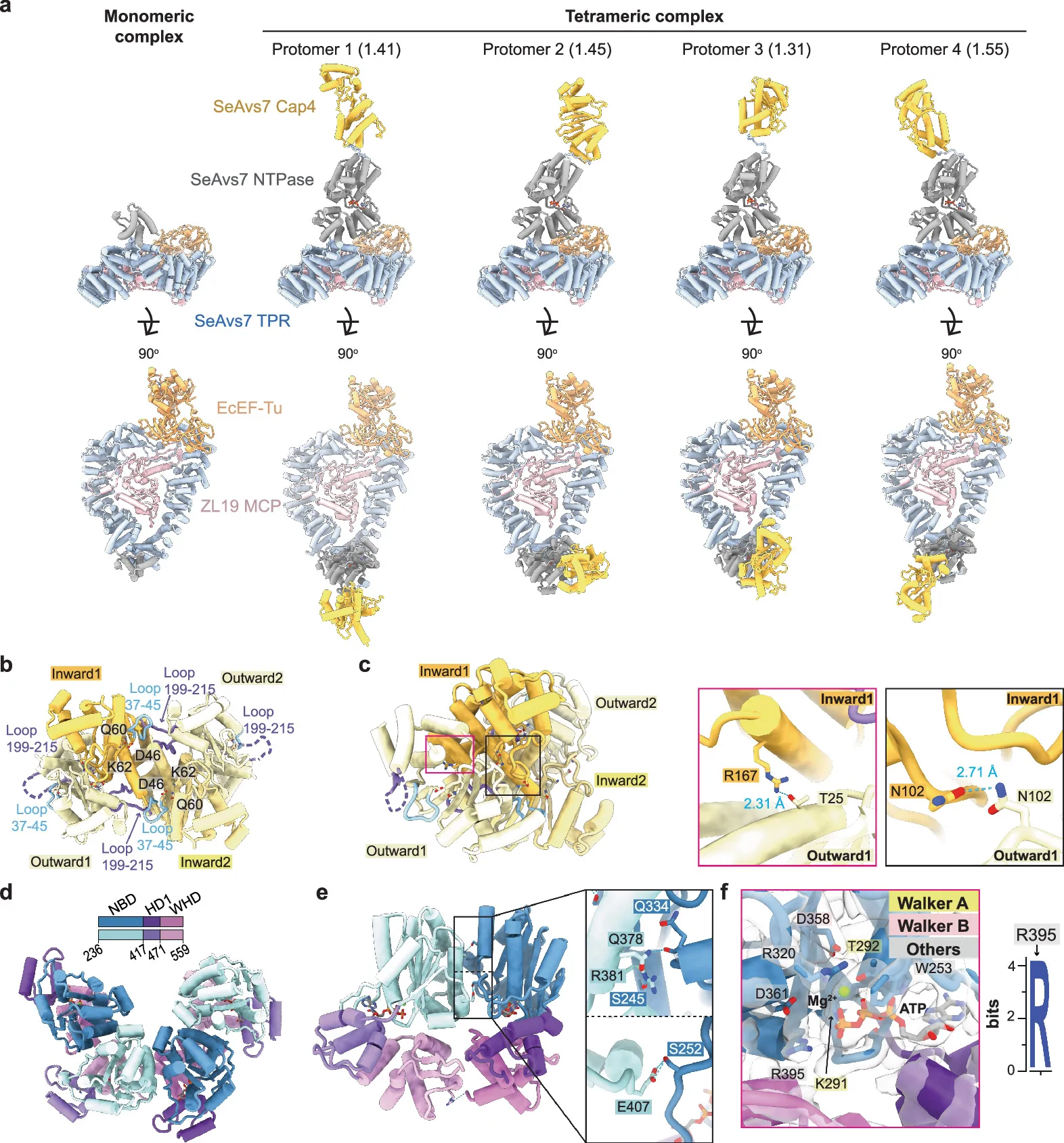
Assembly of the *Se*Avs7–ZL19 MCP–*Ec*EF-Tu tetrameric complex. (**a**) Structural alignment of the monomeric complex with the four protomers of the tetrameric complex. Root mean square deviation (RMSD) values relative to the monomeric complex are indicated in parentheses. (**b**) Structure of the *Se*Avs7 Cap4 nuclease tetramer. (**c**) Interactions between the inward-facing and outward-facing protomers. (**d**) Structure of the *Se*Avs7 NTPase tetramer. (**e**) Molecular interactions between the *Se*Avs7 NTPase domains. (**f**) ATP and Mg^2+^ bound in the active site of the NTPase domain, with the cryo-EM map displayed as a transparent surface. A sequence logo for *Se*Avs7 Arg395, generated from 1,757 Avs7 homologs, is also shown.

To understand how ZL19 MCP and *Ec*EF-Tu interact with *Se*Avs7, we determined the structures of the oligomeric complex by cryogenic electron microscopy (cryo-EM) (Supplementary Fig. 9 and Extended Data Table 1). Refinement revealed two distinct structures: a roughly 1-MDa butterfly-shaped asymmetric tetrameric complex at 3.43-Å resolution containing four copies each of *Se*Avs7, ZL19 MCP and *Ec*EF-Tu, and a smaller, roughly 250-kDa complex at 3.40-Å resolution with a single copy of each protein (Fig. 3a–c). The protomers of the tetramer are structurally similar except for the orientation of the *Se*Avs7 Cap4 nuclease domain (Extended Data Fig. 4a). In contrast to the tetramer, the smaller monomeric complex lacks density for the Cap4 nuclease and the N-terminal part of the NTPase, suggesting that these regions are flexible in the monomeric state but are stabilized on tetramerization (Fig. 3b,c). The remaining parts of the monomeric complex closely resemble a protomer of the tetramer (Extended Data Fig. 4a).

The Cap4 nuclease domains mediate tetramerization by forming a C2-symmetric arrangement with two inward-facing and two outward-facing protomers (Extended Data Fig. 4b). The inward-facing protomers contact each other through helix–helix interfaces, whereas the outward-facing protomers engage in close contacts with the inward ones, forming two hydrogen bonds and burying roughly 12% (roughly 1,300 Å^2^) of their solvent-accessible surface area (Extended Data Fig. 4c). By contrast, the Cap4 tetramer of *Se*Avs3 (ref. ^5^) adopts a distinct arrangement and buries only roughly 2% (roughly 280 Å^2^) of the surface area of its outward-facing Cap4 protomers (Supplementary Fig. 10a). AlphaFold3 modelling placed dsDNA in the *Se*Avs7 Cap4 PD-(D/E)xK active site (Supplementary Fig. 10b), suggesting that the inward-facing protomers are accessible for DNA binding and probably form the active nuclease. Finally, the Cap4 domain is regulated by a flexible loop (residues 41–60) in *Se*Avs3, whereas *Se*Avs7 uses a different extended loop (residues 199–215) for the regulation (Extended Data Fig. 4b).

The overall asymmetry of the *Se*Avs7 complex is established through close contacts between the NTPase domains of adjacent protomers (Extended Data Fig. 4d). Specifically, the nucleotide-binding (NBD) and winged-helix (WHD) subdomains of adjacent protomers interact across a roughly 1,100-Å^2^ interface stabilized by three hydrogen bonds, whereas helical domain 1 (HD1) sits outside this primary interface (Extended Data Fig. 4d,e). Each protomer coordinates ATP and Mg^2+^ through conserved residues (Thr291, Lys292, Asp358 and Arg395) (Extended Data Fig. 4f), consistent with the active conformations of other characterized STAND NTPase oligomers^5,25,31,35^. Notably, although the NTPase tetramer could potentially accommodate a fifth subunit (Supplementary Fig. 10c), the tetramer is sufficient to fully scaffold the assembled Cap4 nuclease, and pentamers were not observed experimentally. These findings indicate that the asymmetric tetramer represents the fully assembled immune complex rather than a partial intermediate.

**Extended Data Fig. 5:**
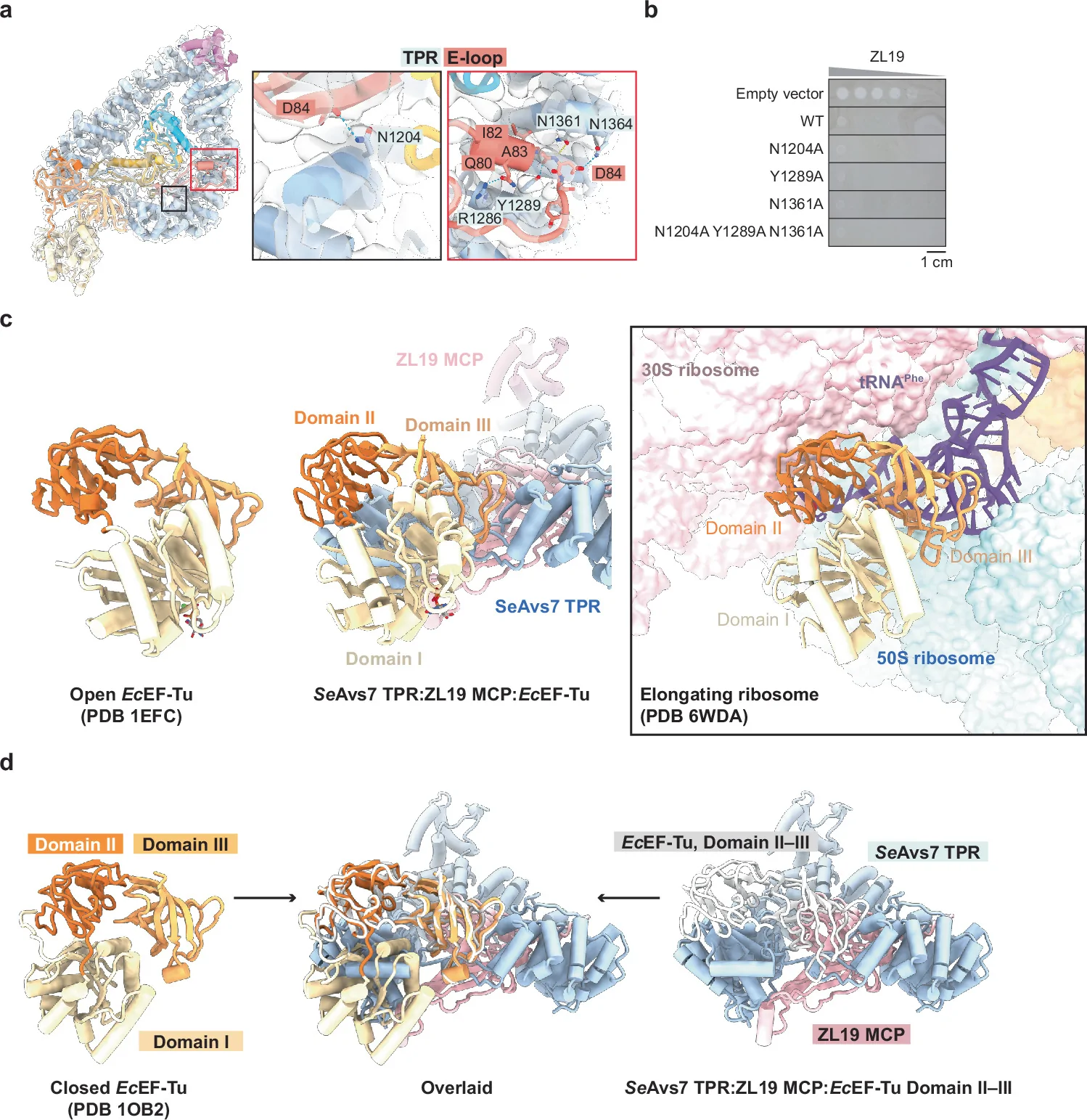
Interactions between the *Se*Avs7 sensor, ZL19 MCP, and *Ec*EF-Tu. (**a**) Molecular interactions between the *Se*Avs7 TPR domain and ZL19 MCP, overlaid with the cryo-EM map (transparent surface). (**b**) Plaque assays of *E. coli* expressing *Se*Avs7 mutants against *Escherichia* phage ZL19. (**c**) Comparison of the *Se*Avs7-bound EcEF-Tu structure (left) with a reference open conformation (PDB 1EFC)^38^. The inset highlights ribosome-bound *Ec*EF-Tu in the open conformation (PDB 6WDA)^37^. (**d**) Structures of closed *Ec*EF-Tu (PDB 1OB2; left) and *Ec*EF-Tu Domains II–III bound to *Se*Avs7 (right), together with a structural superimposition of the two (middle).

**Extended Data Fig. 6:**
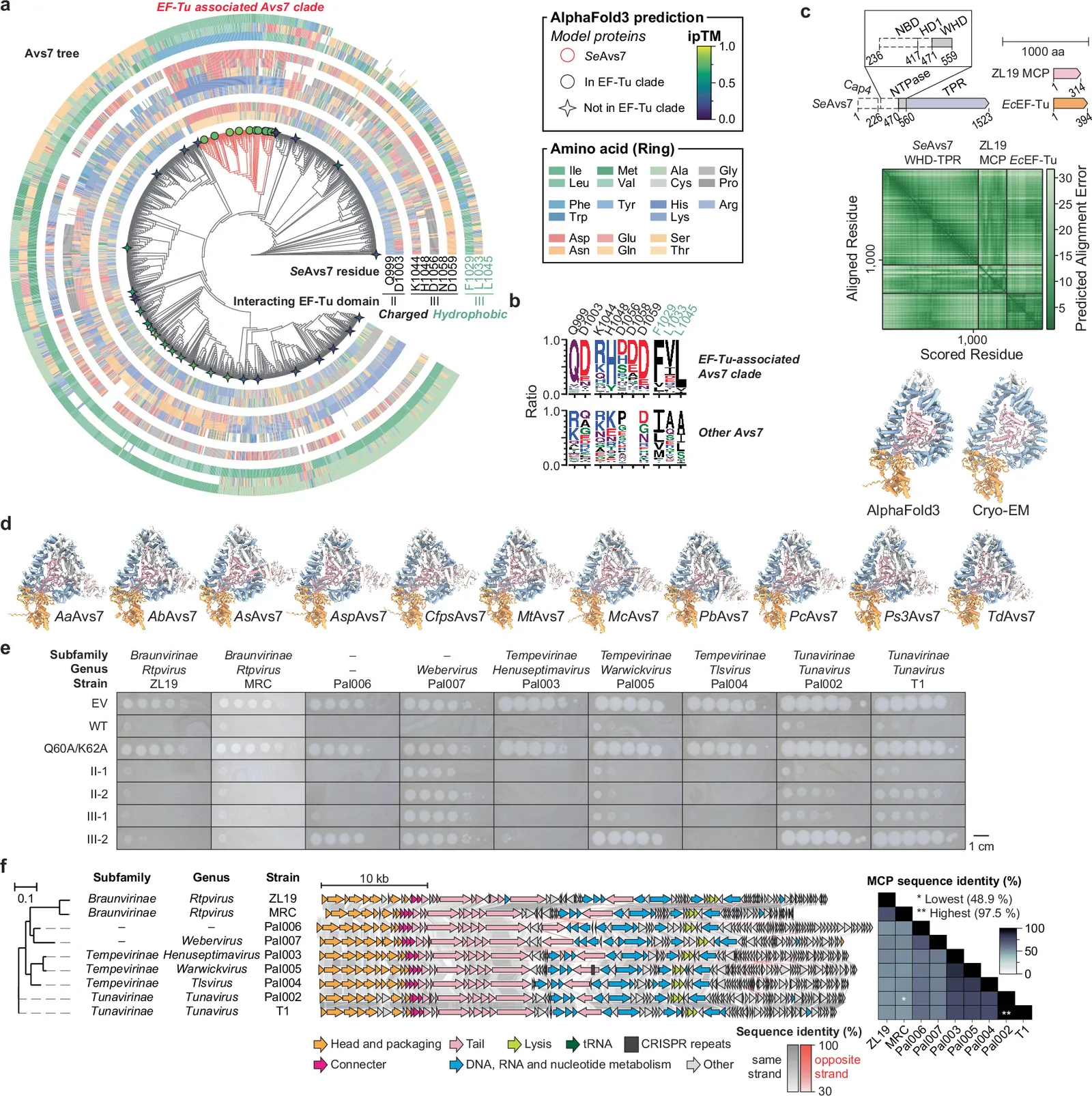
Conservation and functional validation of the Avs7–EF-Tu interaction. (**a**) Phylogenetic tree of 1,757 Avs7 homologs. Color strips represent amino acids at the *Se*Avs7–*Ec*EF-Tu interface. Proteins co-folded with their host EF-Tu and ZL19 MCP are annotated with circles or stars and colored by their ipTM scores. A clade comprising 192 Avs7 homologs likely to form a complex with EF-Tu is highlighted with red branches. (**b**) Sequence logos of *Se*Avs7 residues within the Avs7–EF-Tu interface, comparing the EF-Tu-associated clade (top) with non-associated clades (bottom). (**c**) AlphaFold3 model of the *Se*Avs7 WHD-TPR–ZL19 MCP–*Ec*EF-Tu complex (*Se*Avs7–*Ec*EF-Tu ipTM 0.85), alongside the predicted alignment error (Å). The cryo-EM structure is shown for comparison. (**d**) AlphaFold3 models of 11 Avs7–ZL19 MCP–EF-Tu complexes. Corresponding ipTM scores are provided in Supplementary Table 11. (**e**) Plaque assays of *E. coli* expressing *Se*Avs7 variants against nine *Drexlerviridae* family phages. (**f**) Genome architecture and phylogeny of the nine *Drexlerviridae* phages used in (**e**), and pairwise sequence identities of their respective MCPs. Genomes are shown starting with the 5′ end of the small terminase subunit gene. Arrows are color-coded by predicted function and linked by sequence identity.

The *Se*Avs7 TPR sensor adopts a bilobed structure that envelops the ZL19 MCP, burying 3,700 Å^2^ of its 15,800-Å^2^ solvent-accessible surface (Extended Data Fig. 5a). Binding is driven by shape and charge complementarity between the sensor and the HK97-fold of MCP, including the peripheral (P) domain, axial (A) domain and extended loop^36^ (E-loop; Fig. 3d). The sensor extensively engages with the conserved penton-forming and/or hexon-forming region of the A-domain, while making surface contacts with the variable P-domain and E-loop (Fig. 3d). AlphaFold3 predictions for other Avs7–MCP pairs suggest this broad interface is a conserved feature of Avs7 sensors (Extended Data Fig. 6a–d and Supplementary Fig. 10d). Although we identified five residues potentially involved in hydrogen bond network formation, mutating three of these (Asn1204, Tyr1289 and Asn1361) did not abolish defence against ZL19, suggesting that recognition depends on extensive structural complementarity rather than on specific contacts (Extended Data Fig. 5a,b).

## EF-Tu enhances *Se*Avs7 assembly and activity

While ZL19 MCP is enveloped by the TPR sensor of *Se*Avs7, *Ec*EF-Tu binds the outer surface of the flexible sensor region and adjacent helices through its transfer RNA-binding domains (domain II and domain III)^37^. This interaction buries roughly 1,450 Å^2^ of solvent-accessible surface and is also facilitated by shape complementarity (Fig. 3e). Within this complex, *Ec*EF-Tu adopts the open, inactive conformation typically bound to guanosine diphosphate (Extended Data Fig. 5c). Although the bound nucleotide could not be resolved, structural superposition indicates that the closed, guanosine triphosphate-bound active state would sterically clash with the TPR N-lobe^38^ (Extended Data Fig. 5d), suggesting that *Se*Avs7 specifically co-opts the inactive form of EF-Tu.

The *Se*Avs7–*Ec*EF-Tu interface consists of a network of contacting residues, including several that are conserved within one Avs7 clade (Extended Data Fig. 6a,b). Consistent with this observation, AlphaFold3 confidently predicted Avs7–MCP–EF-Tu complexes exclusively for members of this clade (Extended Data Fig. 6c,d and Supplementary Table 11). To test the functional importance of the interaction, we generated four *Se*Avs7 mutants in which conserved charged residues were targeted (Gln999, Asp1003, Lys1044, His1048, Asp1059; Fig. 3e). None of these mutants copurified with detectable amounts of *Ec*EF-Tu when expressed alone, indicating reduced affinity (Supplementary Fig. 10e).

On reconstitution of the multimeric complex, mutants II-1 (*Se*Avs7^Q999A/D1003A^) and II-2 (*Se*Avs7^Q999A/D1003K^) formed EF-Tu-containing complexes at levels comparable to the wild type; mutant III-1 (*Se*Avs7^K1044A/H1048A/D1059A^) formed less complex and mutant III-2 (*Se*Avs7^K1044E/H1048D/D1059K^) failed to incorporate a detectable amount of *Ec*EF-Tu (Fig. 3f). EF-Tu-mediated enhancement of nuclease activity directly paralleled this assembly efficiency, with wild-type and mutant II-1 strongly enhanced, and mutants II-2, III-1 and III-2 showing progressively weaker effects (Fig. 3g). Plaque assays against nine *Drexlerviridae* family phages (with pairwise sequence identities of MCPs ranging from 48.9% to 97.5%) further corroborated the functional link, showing progressive loss of defence from mutant II-1 to III-2 against five phages, mirroring the trends in complex formation efficiency and nuclease activity enhancement (Fig. 3h and Extended Data Fig. 6e,f). The loss of defence activity by the most disruptive mutant (III-2) did not correlate with either MCP sequence similarity or phage phylogeny, indicating the promiscuity and variable dispensability of the Avs7–EF-Tu interaction across the diversity of phages (Extended Data Fig. 6f). These findings establish *Ec*EF-Tu as a key partner of Avs7 that optimizes phage defence by promoting the formation of the complex between Avs7 and MCP, thereby enhancing nuclease activity.

## MCP binding relieves *Se*Avs7 autoinhibition

**Fig. 4:**
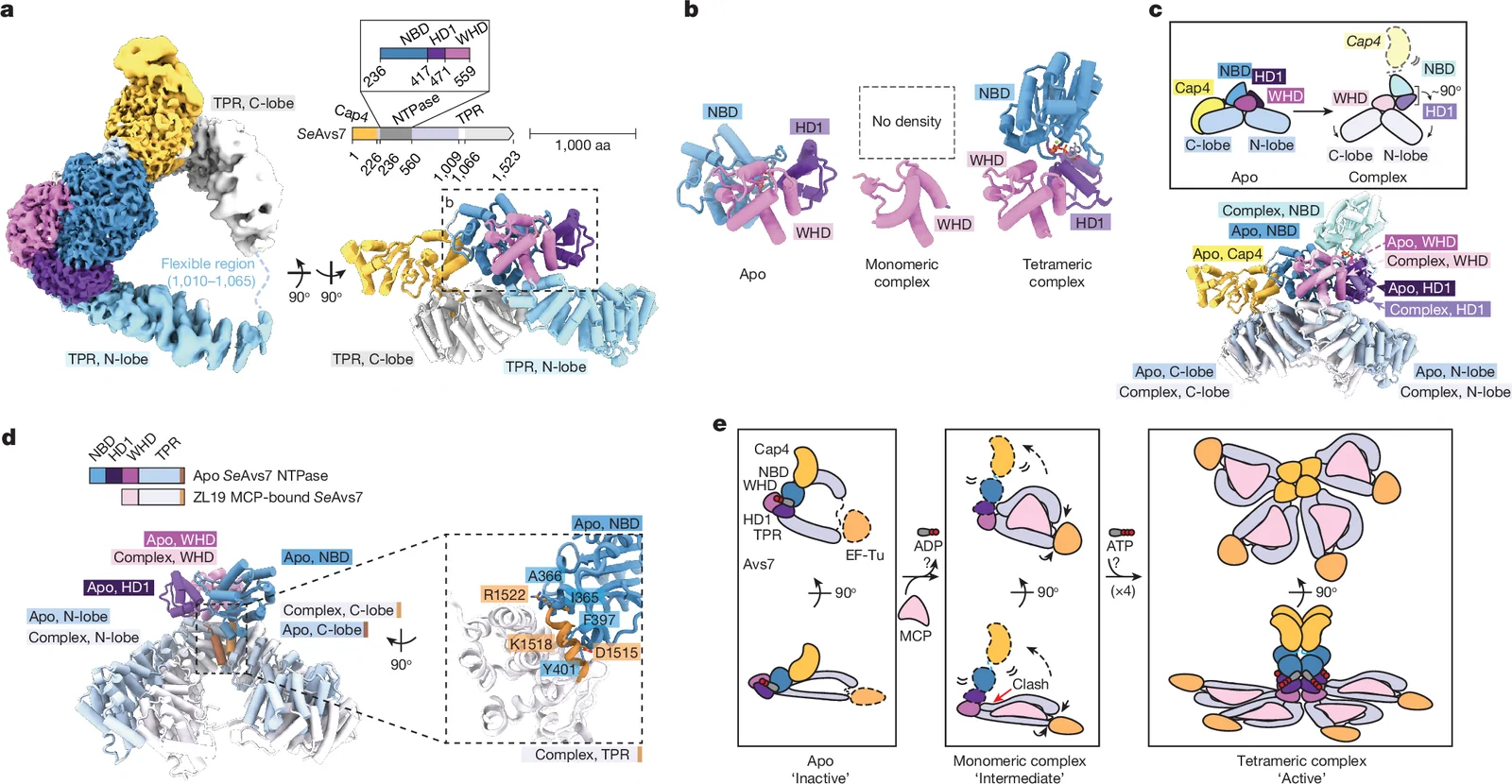
MCP binding induces large structural changes in *Se*Avs7. **a**, Cryo-EM map (left) and structure (right) of apo *Se*Avs7. **b**, Comparison of the NTPase domains in the apo, monomeric and tetrameric states. **c**, Overlay of apo *Se*Avs7 and a protomer of the tetrameric complex, aligned by the WHD. A schematic of the rearrangement is shown in the top inset. **d**, Alternative view of the WHD-aligned overlay in **c**, highlighting the steric clash between the C-terminal helix of MCP-bound *Se*Avs7 (orange) and the NBD of apo *Se*Avs7 (blue). **e**, Proposed mechanism of *Se*Avs7 activation during phage infection.

**Extended Data Fig. 7:**
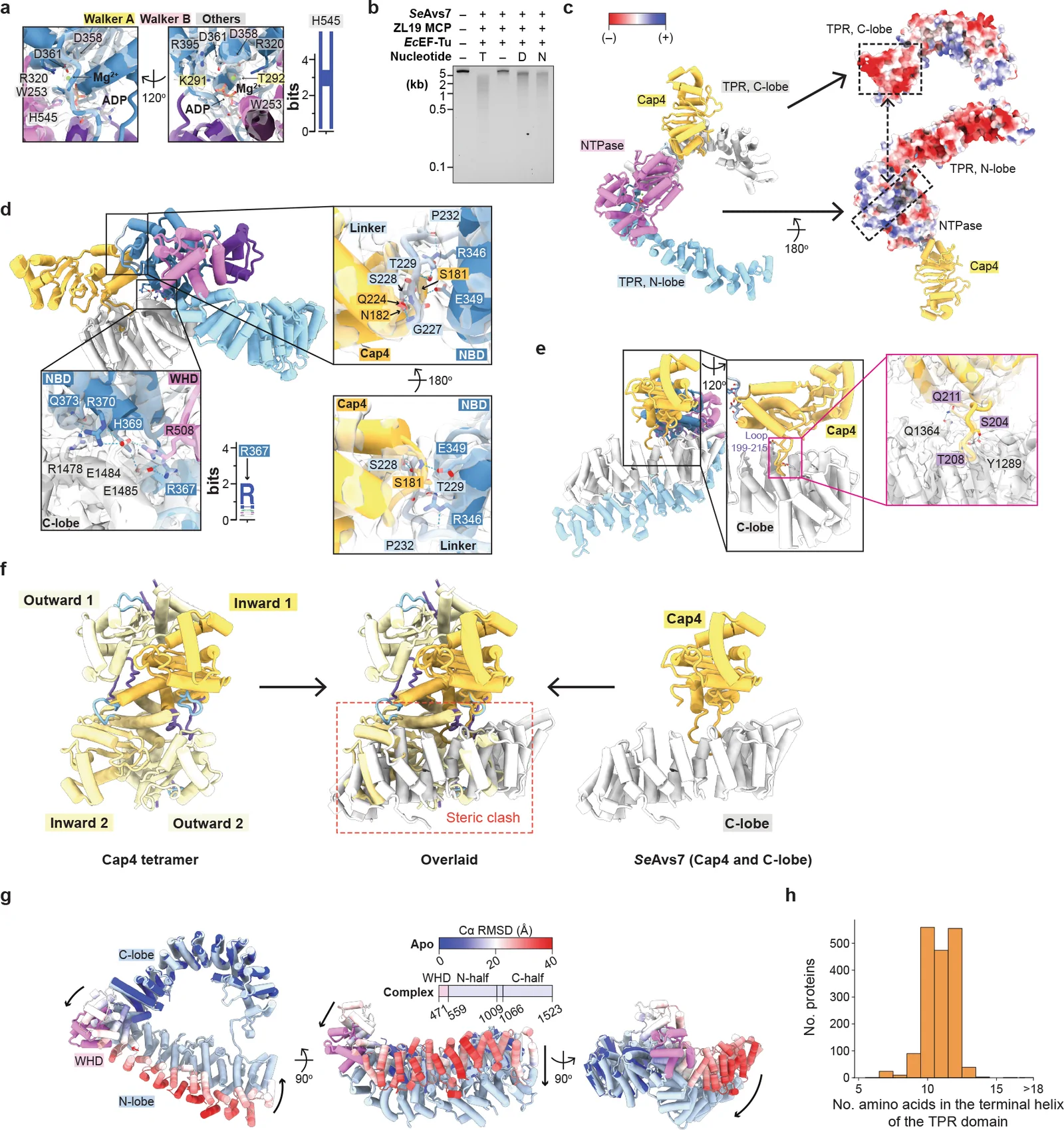
Apo *Se*Avs7 is inactivated by extensive interactions. (**a**) Detailed views of the nucleotide-binding pocket in the apo *Se*Avs7 NTPase domain, with the cryo-EM map displayed as a transparent surface. A sequence logo for *Se*Avs7 His545 is shown. (**b**) Nuclease activity assay in the presence of distinct nucleotides (T, ATP; D, ADP; N, AMP-PNP). For gel source data, see Supplementary Fig. 1. (**c**) Electrostatic surfaces of the interfaces between the NTPase domain and the N-lobe and C-lobe of the TPR domain. (**d**) Interactions within the NTPase–C-lobe and NTPase–Cap4 interfaces, with a sequence logo for *Se*Avs7 Arg367. (**e**) Molecular interactions between Cap4 and the C-lobe. (**f**) Structural overlay of the *Se*Avs7 Cap4 tetramer and auto-inhibited apo *Se*Avs7 (Cap4 and C-lobe). (**g**) Conformational transition of the *Se*Avs7 WHD and TPR domains upon binding of ZL19 MCP. Structures are colored by Cα RMSD values (apo *Se*Avs7 vs. monomeric complex) or by domain (monomeric complex). (**h**) Histogram showing the length distribution of the TPR domain terminal helix across 1,757 Avs7 homologs.

To understand the molecular basis of *Se*Avs7 activation, we determined the structure of apo *Se*Avs7 at 2.72-Å resolution (Fig. 4a, Supplementary Fig. 11 and Extended Data Table 1). Although *Ec*EF-Tu copurified with *Se*Avs7, no corresponding cryo-EM density was observed, suggesting weak association in the absence of MCP (Supplementary Fig. 11a). Compared with the assembled tetramer, the apo structure shows a roughly 90° rotation of the NBD and HD1 relative to the WHD, with none of these domains resolved in the monomeric intermediate (Fig. 4b). Notably, despite ATP being supplied during purification, ADP occupies the NTPase active site and stabilizes a closed conformation (Extended Data Fig. 7a). By contrast, the active tetramer exclusively contains ATP, suggesting ADP-to-ATP exchange during activation. Recognition of both nucleotides involves largely the same residues, except that an Arg395–ATP γ-phosphate contact replaces a His545–ADP β-phosphate contact (Extended Data Fig. 7a). Consistent with this observation, DNA cleavage was slower in the presence of ADP or the nonhydrolysable ATP analogue adenylyl-imidodiphosphate (AMP-PNP), and absent without a nucleotide, indicating that NBD promotes activation through a conformational change rather than through ATP hydrolysis (Extended Data Fig. 7b).

The apo state of *Se*Avs7 is maintained by extensive autoinhibitory interactions. The closed conformation of the NTPase is stabilized by its interaction with the C-lobe through charge complementarity and a network of hydrogen bonds around the partially conserved Arg367 (Extended Data Fig. 7c,d). The Cap4 nuclease domain is sequestered by extensive hydrogen bond contacts with the NBD and by engaging the C-lobe (Extended Data Fig. 7d,e). Superimposing the assembled Cap4 tetramer onto the apo structure reveals severe steric clashes with the apo C-lobe, indicating that tetramerization is incompatible with the apo state (Extended Data Fig. 7f).

Binding of ZL19 MCP triggers global conformational changes that relieve autoinhibition of *Se*Avs7. Superimposing the apo and tetramer structures by the WHD shows that MCP binding causes the TPR N and C lobes to clamp together, displacing them by roughly 30 Å relative to each other (Fig. 4c,d and Extended Data Fig. 7g). This movement leads to steric clashes between the MCP-bound C-lobe and the NTPase helices of the apo form, probably disrupting the interactions that stabilize the inactive state (Fig. 4d). These include a notable clash between the C-terminal helix of the C-lobe (residues 1511–1521) and two NBD helices (residues 360–367 and 396–401). The C-terminal helix is present across Avs7 homologues and has a conserved length (Extended Data Fig. 7h).

On the basis of these observations, we propose a stepwise mechanism for the activation and assembly of Avs7 complexes (Fig. 4e). In the absence of MCP, Avs7 remains in an auto-inhibited, ADP-bound resting state. On phage infection, the Avs7 sensor binds a newly synthesized MCP monomer, strengthened by EF-Tu, clamping the two lobes of the sensor. These conformational changes destabilize the ADP-bound state of the NTPase, probably promoting ADP release and increased flexibility of the N-terminal domains. Finally, ATP binding stabilizes the NTPase, driving tetramerization and enabling Cap4-mediated DNA cleavage to prevent phage replication.

## Thirteen Avs families sense core phage proteins

To investigate the mechanisms of diverse STAND families beyond Avs7, we cloned representative proteins from 30 more sensor structural clusters (Extended Data Fig. 1d–f, Supplementary Fig. 3b and Supplementary Table 6), focusing on *Enterobacteriaceae* homologues, when possible, to facilitate experimental reconstitution in *E. coli*. To identify triggers, we conducted a genetic screen with a barcoded plasmid library of 600 genes from 36 diverse phages and viruses, including single-stranded RNA, single-stranded DNA (ssDNA) and tailed and tail-less dsDNA phages (Supplementary Table 10). We transformed the library into STAND-expressing *E. coli* and deep-sequenced the barcodes to quantify cell death triggered by individual phage genes.

**Fig. 5:**
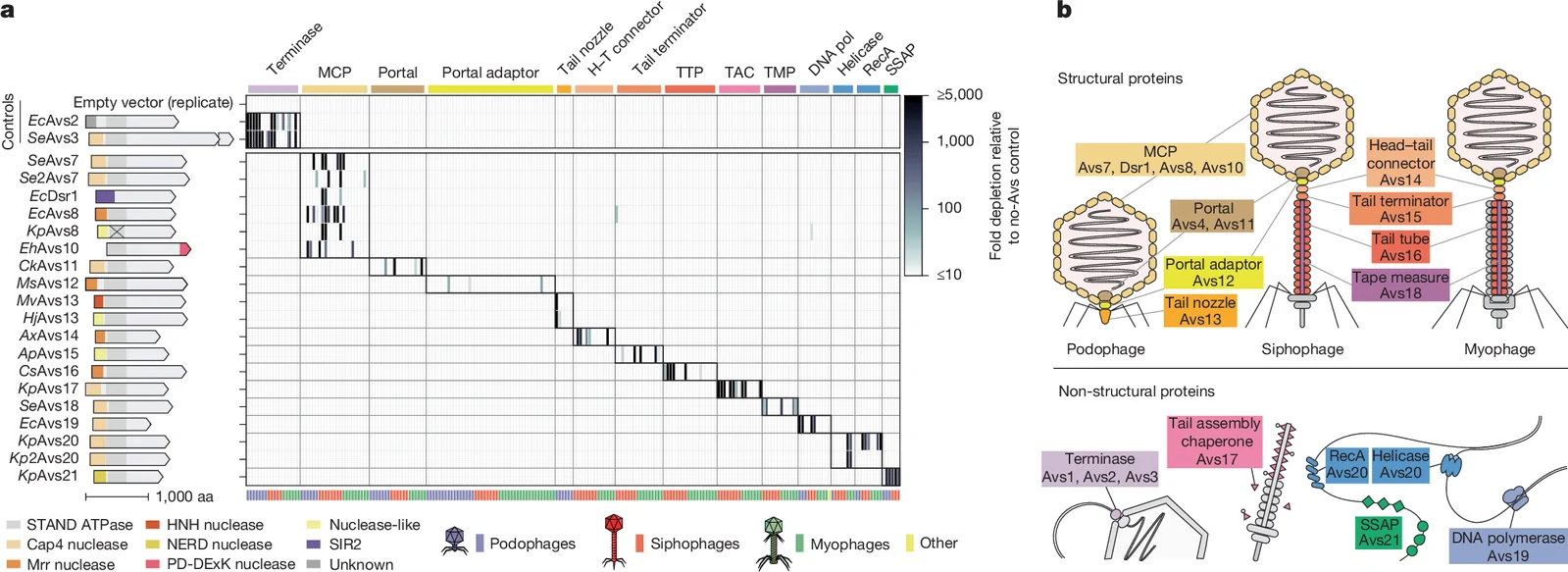
Thirteen additional Avs families are activated by core phage structural and replicative proteins. **a**, Heatmap of Avs-mediated toxicity induced by diverse cognate target proteins. **b**, Functional roles of phage proteins recognized by Avs families. *Ec*, *Escherichia coli*; *Se*, *Salmonella enterica*; *Kp*, *Klebsiella pneumoniae*; *Eh*, *Enterobacter hormaechei*; *Ck*, *Citrobacter koseri*; *Ms*, *Moraxella* sp.; *Mv*, *Myxococcus virescens*; *Hj*, *Halomonas jincaotanensis*; *Ax*, *Alloalcanivorax xenomutans*; *Ap*, *Acinetobacter pittii*; *Cs*, *Cereibacter sphaeroides*; H–T connector, head–tail connector; TTP, tail tube protein; TAC, tail assembly chaperone; TMP, tape measure protein. Source data

**Extended Data Fig. 8:**
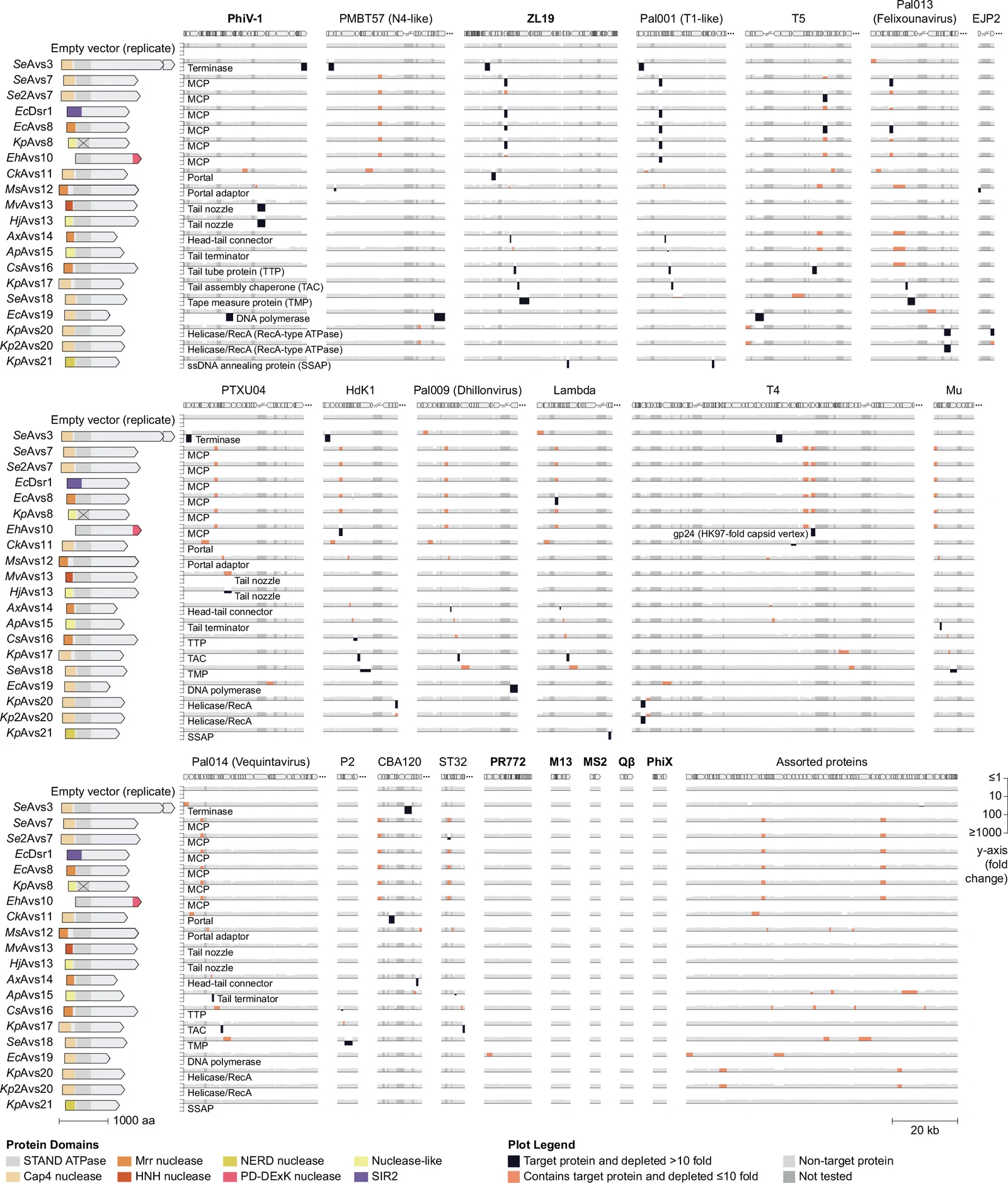
Avs families are activated by specific proteins across phage genomes. Toxicity of Avs genes co-expressed with 600 phage genes in *E. coli*, quantified by barcode abundance following deep sequencing. Bold names indicate phages with complete genomes represented in the library, excluding the dark gray genes due to basal toxicity in *E. coli*. ZL19 data for *Se*Avs7 and *Ec*Dsr1 are replotted from Fig. 2. MCP, major capsid protein. Source data

Thirteen STAND families, which we denote Avs8 and Avs10–21 (because Avs6 and Avs9 now refer to other SAMD9-like families^30^), strongly induced cell death in response to specific phage proteins (Extended Data Fig. 8 and Supplementary Fig. 12). Notably, the identified targets encompassed most of the core structural and replicative proteins of tailed phages^39^ (Fig. 5). Avs8 (PD-λ-4)^14^ and Avs10 (Erebus/Hypnos/bNACHT64)^16,17^ sensed MCPs, consistent with recent findings for Avs8 (ref. ^40^), whereas Avs12–18 recognized seven distinct structural proteins: portal adaptor, tail nozzle, head–tail connector, tail terminator, tail tube protein, tail assembly chaperone and tape measure protein, respectively (Extended Data Fig. 8). By contrast, Avs19–21 sensed replicative proteins: family A DNA polymerases, the RecA-type ATPase domain in both the primase-helicase and RecA and ssDNA annealing protein (SSAP), respectively (Extended Data Fig. 8). Avs11 recognized the portal protein despite lacking sequence and structural similarity to the previously characterized portal-sensing Avs4 (Extended Data Fig. 8 and Supplementary Fig. 3). In all cases, mutating or deleting the effector domains abolished cell death (Supplementary Fig. 13).

**Extended Data Fig. 9:**
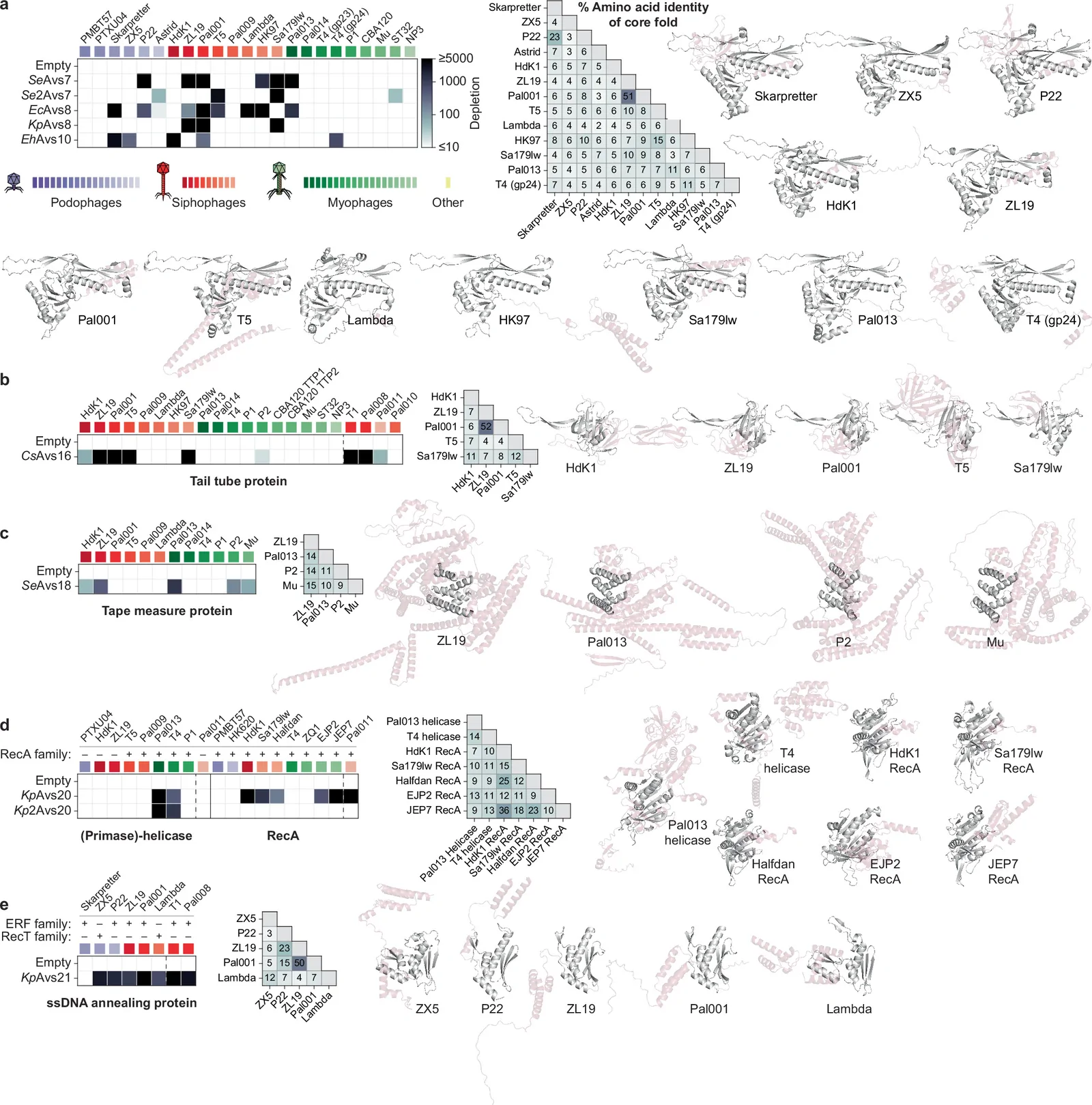
Avs pattern recognition of conserved core structural folds. Toxicity quantification and structural comparisons of AlphaFold3 models for (**a**) major capsid protein, (**b**) tail tube protein, (**c**) tape measure protein, (**d**) primase-helicase and RecA, and (**e**) ssDNA annealing protein. Toxicity data are replotted from Fig. 5a and Supplementary Fig. 14. Pairwise amino acid identities between representative core folds are shown; corresponding AlphaFold3 structures highlight these folds in dark gray. Amino acid identities below ~5% are considered random. NP3, *Pseudomonas* phage NP3.

To evaluate the specificity of the identified interactions, we assembled a panel of 218 target homologues, combining hits from the genetic screen with 60 newly cloned additions to ensure at least 6 diverse homologues per target protein (Supplementary Table 12). We quantified all 4,360 pairwise interactions between this panel and 18 Avs representatives by means of deep sequencing, and selectively tested an extra 27 genes (Supplementary Fig. 14 and Supplementary Table 13). Consistent with the initial screen, each Avs was triggered by homologous proteins from different phages, with minimal cross-reactivity against non-cognate targets (Fig. 5a). Within each target family, Avs proteins recognized a broad range of homologues, in many cases with little to no sequence similarity, but sharing the same core fold (Extended Data Fig. 9 and Supplementary Fig. 15). For instance, Avs21 could detect both ERF-family and RecT-family SSAPs, which have virtually no sequence similarity^41^. The conservation of Avs target proteins mirrors the overall synteny of structural genes across phage genomes (Supplementary Fig. 16).

**Extended Data Fig. 10:**
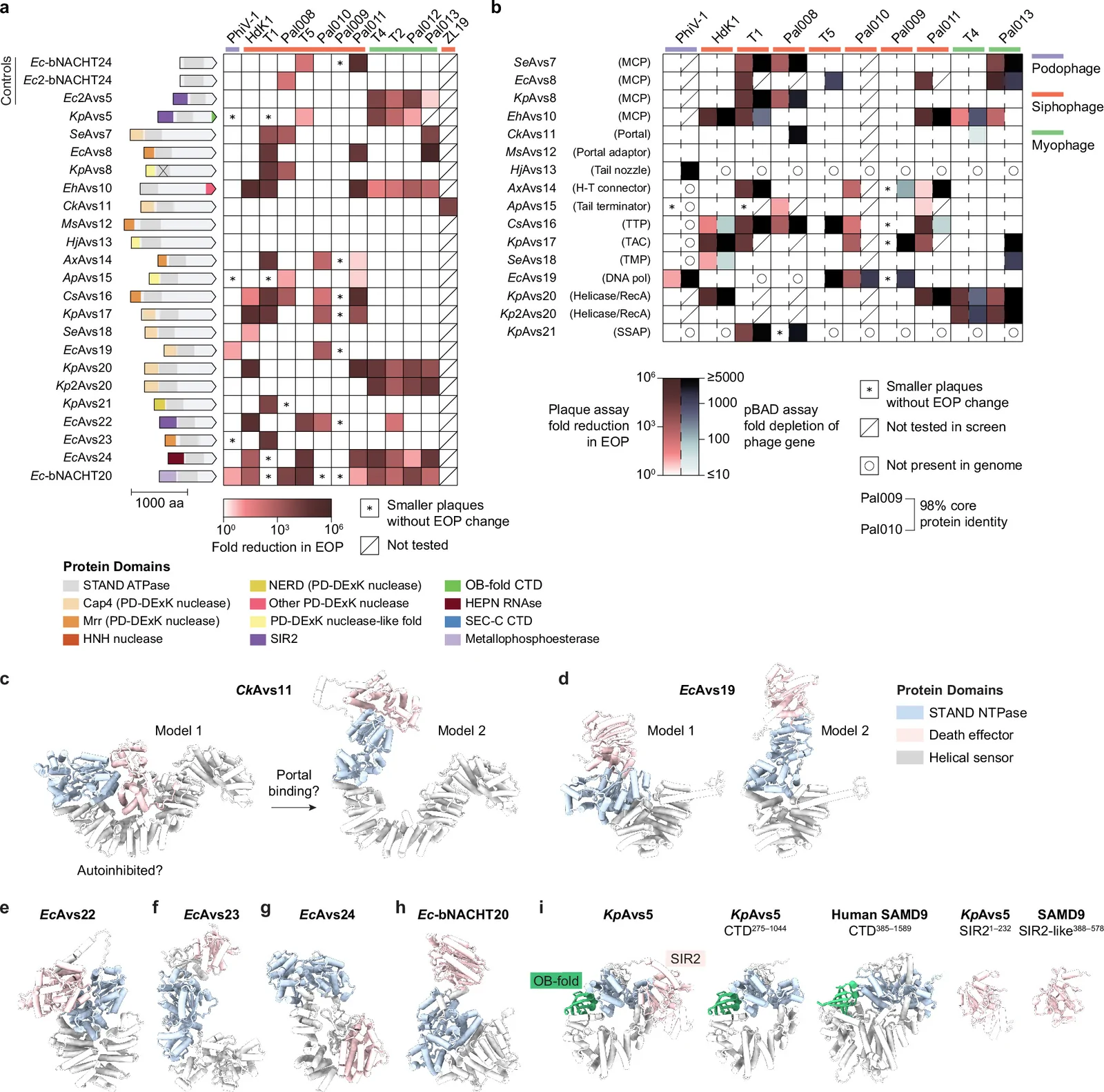
Anti-phage defense activity of Avs proteins. (**a**) Efficiency of plating (EOP) of a panel of phages against Avs proteins in *E. coli*. Raw images are shown in Supplementary Fig. 17. (**b**) Comparison of phage defense (EOP fold reduction) with co-expression toxicity for each Avs protein and its respective trigger. (**c–h**) AlphaFold2/3 models of (**c**) *Ck*Avs11, (**d**) *Ec*Avs19, (**e**) *Ec*Avs22, (**f**) *Ec*Avs23, (**g**) *Ec*Avs24, and (**h**) *Ec*-bNACHT20. (**i**) Structural comparison of *Kp*Avs5 and human SAMD9.

Supporting these findings, most tested Avs families mediated anti-phage defence in *E. coli* against at least one of 12 tested coliphages. Specificities generally aligned with their identified genetic triggers, with a few exceptions (Extended Data Fig. 10a,b and Supplementary Fig. 17). Anti-phage activity was also detected for four more sensor structural clusters (Avs22–24 and bNACHT20) for which no phage-encoded activators were identified in our screen (Extended Data Fig. 10a and Supplementary Fig. 17). Further investigation is needed to determine how these Avs families sense phage infection.

## Avs proteins recognize core phage structural patterns

**Fig. 6:**
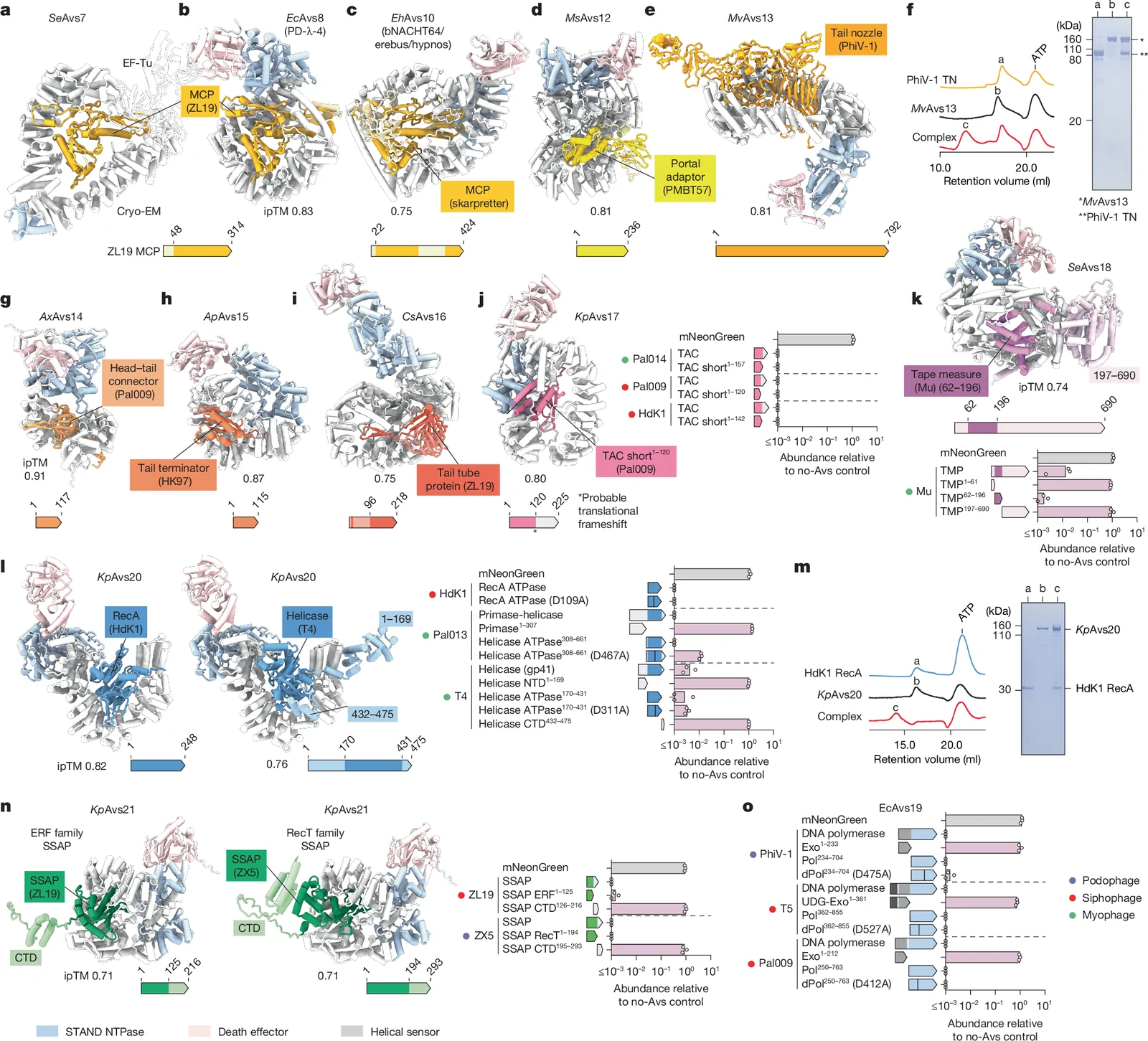
Avs proteins recognize core structural patterns through shape complementarity across extended binding interfaces. **a**–**e**,**g**–**l**,**n**, AlphaFold2/3 models of Avs proteins binding the MCP (*Se*Avs7, **a**; *Ec*Avs8, **b**; *Eh*Avs10, **c**), portal adaptor (**d**), tail nozzle (**e**), head–tail connector (**g**), tail terminator (**h**), tail tube protein (**i**), tail assembly chaperone (**j**), tape measure protein (**k**), helicase/RecA-fold ATPase (**l**) and SSAPs (**n**), accompanied by quantification of Avs activation by truncated or catalytically inactivated target proteins. Bars represent the mean of three barcode replicates. **f**,**m**, Size-exclusion chromatograms and SDS–PAGE analysis of *Mv*Avs13–PhiV-1 tail nozzle (**f**) and *Kp*Avs20–HdK1 RecA (**m**) complexes are shown. See Supplementary Fig. 1 for uncropped gel images. **o**, *Ec*Avs19 activation by truncated or catalytically inactivated family A DNA polymerases. TN, tail nozzle; NTD, N-terminal domain; CTD, C-terminal domain; UDG, uracil-DNA glycosylase. ipTM values refer to protein–protein pairs only and exclude interactions with ligands (for example, ATP and Mg^2+^). Source data

**Extended Data Fig. 11:**
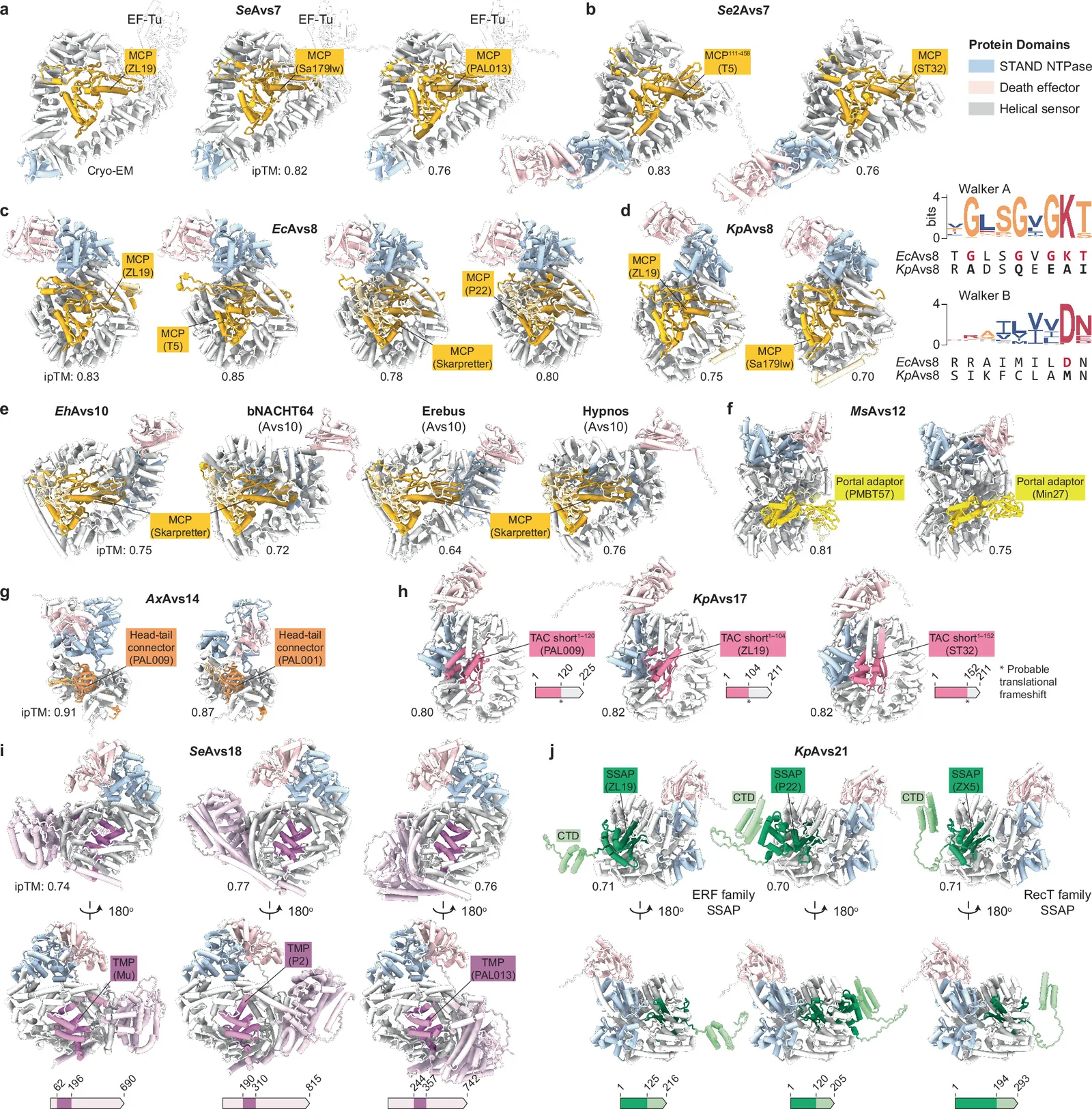
Additional structural models of Avs pattern recognition of phage proteins. AlphaFold2/3 models of (**a**) *Se*Avs7, (**b**) *Se*2Avs7, (**c**) *Ec*Avs8, (**d**) *Kp*Avs8, (**e**) Avs10 homologs, (**f**) *Ms*Avs12, (**g**) *Ax*Avs14, (**h**) *Kp*Avs17, (**i**) *Se*Avs18, and (**j**) *Kp*Avs21 binding their respective target proteins. The ATPase Walker A and Walker B motifs of *Ec*Avs8 and *Kp*Avs8 are shown in (d); the degenerate motifs in *Kp*Avs8 suggest a lack of ATPase activity.

To determine how Avs8 and Avs10–21 engage their targets, we generated AlphaFold3 or AlphaFold-multimer models, obtaining confident predictions (interface predicted template modelling score (ipTM) 0.71–0.91) for 11 of the 13 complexes (Fig. 6, Extended Data Fig. 11 and Supplementary Fig. 18). In each case, the Avs TPR-like sensor bound a single copy of the target protein through an extensive, shape-complementary interface. In vitro reconstitution of two complexes (*Kp*Avs20–HdK1 RecA and *Mv*Avs13–PhiV-1 tail nozzle) biochemically confirmed direct binding and subsequent Avs oligomerization (Fig. 6f,m).

The Avs sensors consistently recognized the conserved core folds of their targets, leaving variable extensions unbound (Extended Data Figs. 9 and 11 and Supplementary Fig. 15). For example, despite their different sensor architectures, Avs7, Avs8 and Avs10 all bind the penton-forming and/or hexon-forming interface of the MCP A-domain (Fig. 6a–c). Avs12–15 envelop the globular folds of their single-domain targets^42^ (Fig. 6d,e,g,h). Avs16 forms extensive contacts with the conserved β-hairpin of the tail tube protein, which constitutes the interface between stacked hexameric rings of the tail tube (Fig. 6i). Avs17 binds the N-terminal core of the TAC, a region shared by both long and short isoforms generated through programmed translational frameshifting^43^ (Fig. 6j). For *Se*Avs18, models indicate binding to a central roughly 130-residue α-helical segment of tape measure proteins, which PROMALS3D (ref. ^44^) independently identified as the most conserved region. This segment was necessary and sufficient to activate cell death (Fig. 6k). Core fold recognition extended to replicative proteins: Avs20 binds the RecA-type ATPase domain that is present in both primase-helicase and RecA^45^, independent of ATPase catalytic activity (Fig. 6l), and Avs21 recognizes the N-terminal ssDNA-binding domain of both ERF-family and RecT-family SSAPs (Fig. 6n).

Although high-confidence models could not be generated for *Ck*Avs11–portal and *Ec*Avs19–DNA polymerase complexes, predictions of apo Avs proteins suggest autoinhibition that might be released by target binding (Extended Data Fig. 10c,d). The core polymerase domain was necessary and sufficient to activate *Ec*Avs19, independent of the polymerase catalytic aspartate (Fig. 6o).

Altogether, our structural modelling and mutational analyses demonstrate that Avs families recognize the conserved core structures of highly divergent target proteins (Fig. 6, Extended Data Fig. 11 and Supplementary Fig. 18). This recognition appears to target monomers, as structural modelling showed major steric clashes with their oligomerized or substrate-bound forms (Supplementary Fig. 19).

## Discussion

Here we present a systematic study of the diversity and activation of STAND defence systems across prokaryotes. Together with the previously characterized terminase-sensing Avs1–3 (ref. ^5^), our findings demonstrate that dedicated Avs families monitor most of the conserved core proteins of tailed phages. At least 12 of the 14 investigated families engage their targets through broad shape-complementary interfaces that envelop the target core, a mechanism that distinguishes Avs sensors from most other known protein-sensing defence systems. For instance, three prevalent families (Avs7, Avs8 and Avs10) wrap around the HK97-fold MCP, contrasting systems such as Lit protease^46,47^ and CapRel^48^, which detect MCP through short sequence motifs.

Of the 13 identified targets, 5—tail nozzle, portal adaptor, head–tail connector, tail terminator and tape measure protein—have not, to our knowledge, been previously known to trigger defence. For three replicative proteins—primase-helicase, RecA and SSAP—phage escape mutations have been previously identified^49–52^, but the mechanisms of sensing remained unknown and could be indirect. For instance, the Hachiman/AbpAB defence system detects genome integrity rather than replicative proteins themselves^53^. Altogether, our findings substantially expand the repertoire of phage proteins that directly activate prokaryotic defence systems.

Our structural and biochemical analyses of *Se*Avs7 reveal a previously unknown mechanism of host-factor repurposing in anti-phage defence. The structural role of EF-Tu in the Avs7 complex contrasts with its involvement in the functioning of other defence systems such as Lit protease^46,54^ and type IV Thoeris^55^ systems, where EF-Tu is cleaved to arrest translation. Instead, this mechanism is reminiscent of the ribosomal protein S15 in the type V-K CRISPR-associated transposon complex^56^, which facilitates DNA mobilization. Another relevant case of EF-Tu repurposing is its recruitment (along with EF-Ts) as an accessory subunit by the RNA-dependent RNA polymerase of *Leviviridae* family phages^57^. By co-opting highly abundant and conserved host proteins, both defence systems and mobile elements appear to achieve a reduction in their own protein investment without substantially perturbing the host proteome^58^. These observations parallel those for several eukaryotic NLRs that bind host factors, undergoing spatial or conformational changes on infection^24,59^. Alternatively or in addition, given that T4 MCP binds EF-Tu^47^, Avs7 might have evolved to exploit a pre-existing interaction between certain MCPs and EF-Tu, a mechanism that parallels sensing of DnaJ (a host chaperone) perturbation by bNACHT25 (ref. ^8^).

Our findings do not exclude the existence of other, noncanonical activating mechanisms for the Avs families described here. Indeed, alternative activators have been identified for CapRel^60^ and Avs2 (ref. ^6^). Avs activation also might require more host factors beyond their phage target, as exemplified by EF-Tu recruitment by Avs7 and DnaJ perturbation by bNACHT25 (ref. ^8^). Further studies are required to elucidate the full repertoire of these activation mechanisms. Nonetheless, the conservation and broad structural complementarity of the identified targets with the Avs sensor domains suggest that these interfaces are favoured during the host–virus arms race, enabling sensing of a broad range of phages while avoiding autoimmunity.

Beyond the 13 targets identified here, the full sensory repertoire of prokaryotic STANDs remains an open question. More STAND families might detect other essential phage components that are more variable or lineage-specific, such as the small terminase subunit, tail sheath, minor tail proteins and baseplate hub, or other uncharacterized proteins^6,7,9^. Furthermore, it remains a possibility that some sense molecules other than proteins. Our phylogenetic analysis and structural classification of the STANDs provide a resource for future investigation.

In addition to their fundamental role in phage defence, the mechanism of Avs sensors offers insights for therapeutic innovation. The discovery that bacterial defence systems actively monitor conserved, essential phage structures exposes potential vulnerabilities that could be exploited for off-the-shelf therapeutic phage design. Conversely, the finding that small phage proteins can trigger Avs-mediated cell death suggests the possibility of developing new antimicrobials that artificially activate these pathways in antibiotic-resistant pathogens. Altogether, our findings reveal that structure-based pattern recognition of the core phage proteome is a main defence strategy in prokaryotes, highlighting sophisticated adaptations in the host–virus arms race.

## Methods

### Bioinformatics analysis of STAND NTPases

To systematically identify defence-related STAND NTPases in prokaryotes, we curated an initial set of 656 diverse STAND ‘seed’ sequences (Supplementary Table 1). This set included representatives of all previously predicted prokaryotic defence-related STANDs^2,4,16–18^, covering both NACHT and other clades. We also included extra sequences from GenBank identified through BLAST searches using the known STANDs and the defence-associated effector proteins Cap4, Mrr, Trypco2 and metallo-β-lactamase^5^.

The 659 NTP-binding domains within the 656 seed sequences were clustered, aligned and used as queries in PSI-BLAST^61^ searches against the National Center for Biotechnology Information (NCBI) clustered_nr database (non-redundant proteins clustered at 90% identity and 90% coverage) to identify a preliminary set of homologues. Footprints of these searches were then aligned with the corresponding queries and used in a second round of PSI-BLAST searches against the same database, resulting in the identification of 1,750,195 NTP-binding domains from 1,708,335 proteins. These NTP-binding domains, originating from both prokaryotes and eukaryotes, were clustered using MMSeqs2 (ref. ^62^) at 75% identity and 67% coverage, yielding 680,395 clusters.

Representatives of these 680,395 clusters (one from each cluster) were selected and further clustered using MMSeqs2 at 33% identity and 67% coverage, producing 37,834 clusters. Sequences within each new cluster were aligned using MUSCLE5 (ref. ^63^) and the resulting 37,834 consensus sequences^64^ were aligned using FAMSA^65^. Each character of the aligned consensus sequence was then expanded into a corresponding column of the original cluster multiple sequence alignment (MSA), generating a pseudo-MSA of 680,395 representative NTP-binding domain sequences.

A preliminary approximate maximum likelihood tree was reconstructed from these sequences using FastTree^66^ with gamma-distributed site rates and the WAG evolutionary model. This tree was cut at a depth of 1.5 from the tips into 17,857 clades. The domain content of the 680,395 sequences across these clades were annotated using PSI-BLAST against the NCBI CD database^67^; clades containing potential defence-related NTPases were identified by the presence of likely sensor and effector domains. This procedure identified 884 potential defence-related clades, totalling 359,879 leaves corresponding to NTP-binding domain sequences. The discarded clades mainly consisted of homologous but distantly related P-loop domains identified by PSI-BLAST, such as ABC transporters, adenylate kinases and Clp proteases, or were singleton outliers.

This subset of 359,879 leaves was further clustered using MMSeqs2 at 25% identity and 67% coverage, resulting in 2,488 clusters. Each cluster was aligned using MUSCLE5, and the 2,488 consensus sequences were aligned using FAMSA. Each character of the aligned consensus sequence was expanded into a corresponding column of the original cluster MSA, resulting in a pseudo-MSA of 359,879 representative NTP-binding domain sequences. A final approximate maximum likelihood tree was reconstructed from these sequences using FastTree with gamma-distributed site rates and the WAG evolutionary model. The tree was cut at a depth of 2.0 from the tips, yielding 589 final STAND clades (Supplementary Table 2). The tree was visualized using ggtree v.3.10.1 in R^68^.

Each of the 589 STAND clades was classified as defence-associated if either or both of the following criteria were met: the presence of defence effector domains within the protein sequences or statistical enrichment of genomic context adjacent to known defence systems.

- The presence of effector domains was assessed using the following criteria: clades in which at least 50% of the representative sequences contained previously annotated effector domains (detected by PSI-BLAST using Conserved Domain Database profiles; Supplementary Table 3) were classified as high-confidence defence clades (86 clades), and those with 10–50% of sequences containing these domains were considered low-confidence defence clades (86 clades).
- To assess the genomic context of each STAND clade, we used DefenseFinder^69^ to identify known anti-phage defence genes encoded within 5 kb of representative STAND sequences. For each clade, to determine whether the observed proximity to defence genes was significantly greater than expected by chance, we calculated a *P* value using the one-sided binomial distribution. The null probability was set at 0.0153, which reflects the chance that a randomly selected non-defence gene would be located within 5 kb of a defence system, based on our calculated DefenseFinder statistics. Clades with a *P* value less than or equal to 0.0001 were classified as high-confidence defence-associated clades, resulting in 131 such clades (Supplementary Fig. 1b).

This combined classification scheme yielded 198 high-confidence defence-associated STAND clades (Supplementary Table 4). To assess sensor diversity, sequence clusters within these clades (clustered at 75% identity and 67% coverage; Fig. 1a, step 3) were expanded, resulting in 178,641 proteins. From these, C-terminal regions following the NTPase domains were extracted, and sequences shorter than 400 amino acids were excluded, yielding 34,990 sequences. These sequences were then clustered using MMseqs2 (ref. ^62^) at 80% coverage and an *E* value threshold of 10^−5^, resulting in 1,479 sequence clusters.

For each sequence cluster, a representative AlphaFold structure was either retrieved from the AlphaFold database (https://alphafold.ebi.ac.uk/)^70,71^ or predicted using AlphaFold3 (ref. ^33^), using the full protein sequence along with one ADP molecule and one Mg^2+^ ion. Seventeen eukaryotic clusters were excluded, and five clusters did not yield structural models. From the remaining 1,457 representative structures, the C-terminal regions following the NTPase domains were extracted and clustered using Foldseek^72^ at 80% coverage, an *E* value of 10^−10^ and a template modelling (TM) score threshold of 0.40, resulting in 684 structural clusters (Supplementary Table 5). For visualization, 90 sensor structural clusters were selected, on the basis of (1) each cluster containing at least five members and (2) at least 25% of cluster members belonging to defence-associated STAND clades.

### Prophage prediction

Prophage regions were detected using PHASTEST^73^.

### Bioinformatics analysis of Avs7 and Dsr1

Avs7 homologues (Supplementary Table 7) were identified using PSI-BLAST searches against the NCBI non-redundant protein sequence database in July 2024. After filtering out truncated sequences and non-Avs7 hits, a final set of 3,272 verified Avs7 sequences was obtained. These sequences were then clustered at 90% identity using MMseqs2 (ref. ^62^), resulting in 1,757 clusters, and 1 representative from each cluster was selected for subsequent analysis. An MSA of these representatives, excluding their variable N-terminal domains, was generated using MAFFT v.7.520 (ref. ^74^) with global pairwise alignment (–maxiterate 1000–globalpair). The alignment was trimmed with trimAl 1.2 (ref. ^75^) using a gap threshold of 0.25 (-gt 0.25). Phylogenetic trees were constructed from the trimmed MSAs using IQ-TREE v.1.6.12 (ref. ^76^) with the LG + G4 model and 2000 ultrafast bootstrap replicates (parameters -nstop 500 -safe -nt 4 -bb 2000 -m LG + G4). N-terminal effectors were annotated based on HHpred^77^ predictions, and taxonomic classifications were retrieved from the NCBI taxonomy database. The tree was visualized using iTOL^78^.

To identify more defence proteins that share the Avs7 sensor domain, PSI-BLAST searches were performed against the NCBI non-redundant protein sequence database downloaded in February 2024. The sensors from two Avs7 representatives (OJX47172.1 and WP_079981830.1) were used as queries for ten and nine iterations, respectively, with a maximum *E* value threshold of 0.001 and a minimum sequence coverage of 70%. These searches yielded an initial list of 10,249 proteins. Given the high number of Dsr1 homologues in this list, we conducted an extra PSI-BLAST search using the sensor domain of *Ec*Dsr1 (WP_029488749.1) for 16 iterations, which added 45 unique proteins.

A round of curation to remove partial proteins and false positives yielded 9,097 proteins containing an Avs7 sensor domain (Supplementary Table 8). These sensor domains were extracted and clustered at 95% sequence identity and 98% coverage using MMseqs2 (ref. ^62^). An MSA of 4,969 representative sequences was generated using MAFFT v.7.525 (ref. ^74^) with global pairwise alignment. The alignment was trimmed by trimAl v.1.5 (ref. ^75^) with a gap threshold of 0.25. A phylogenetic tree was constructed from the trimmed alignment using IQ-TREE v.2.3.6 (ref. ^79^) with the LG + G4 model and 2,000 ultrafast bootstrap replicates. Protein domains were predicted using HHpred^77^, and for split proteins, adjacent sequences were also analysed. Taxonomic classifications were retrieved from the NCBI taxonomy database, and the tree was visualized using the R package ggtree v.3.10.1 (ref. ^68^).

### Cloning

Genes were chemically synthesized (Twist Bioscience or Integrated DNA Technologies) or amplified by polymerase chain reactions from genomic DNA using Q5 (New England Biolabs) or Phusion Flash (ThermoFisher) polymerases. Plasmids were constructed with Gibson assembly and transformed into *E. coli* strains Stbl3 or Mach1 (ThermoFisher). *E. coli* colonies were grown in 1.8 ml of Terrific Broth overnight at 37 °C with 100 μg ml^−1^ carbenicillin or 25 μg ml^−1^ chloramphenicol, as appropriate. Plasmids were isolated using Qiagen miniprep buffers and 96-well DNA and RNA binding plates (Epoch Life Science). The full sequences of all plasmids were verified with Tn5 tagmentation and Illumina sequencing, as previously described in refs. ^4,80^.

STAND NTPases and mutants were cloned into a low-copy pACYC184-like plasmid backbone with a chloramphenicol resistance gene (Supplementary Table 6). For genes from *Enterobacteriaceae*, the native promoter and native DNA sequence were retained in most cases (66 systems), with the exception that the lac promoter was used if the gene was separated by fewer than 50 base pairs (bp) from the preceding open reading frame in the genome, or if the promoter could not be synthesized (4 systems). Genes outside *Enterobacteriaceae* (40 systems) were codon optimized for expression in *E. coli* K-12 and cloned after a lac promoter.

Phage genes and mutants were cloned downstream of an arabinose-inducible pBAD promoter into a pBR322-like plasmid backbone with an ampicillin resistance gene and a 20 bp barcode sequence unique to each clone. One to three clones for each construct, each with a distinct barcode, were retained for inclusion in the phage gene library (Supplementary Tables 10 and 13).

### Phage samples

Phages T4, T5, PhiV-1, P1, Lambda, M13, MS2, Qβ, PhiX and PR772 were obtained from the American Type Culture Collection (ATCC). Phages HdK1, PTXU04 and PMBT57 were obtained from the Leibniz Institute DSMZ. Phages Pal001–014 were isolated from wastewater treatment samples from Palo Alto, California, USA. Wastewater samples were passed through a 0.22-μm syringe filter (Cytiva Whatman), 10^2^-fold, 10^3^-fold or 10^4^-fold diluted in water, and plated on top agar containing *E. coli* K-12 strains ATCC 25404 or BW25113 Δ*iscR* (ref. ^81^), similar to phage plaque assays. After overnight incubation at 37 °C, phages from individual plaques were isolated from the top agar.

Phages used in plaque assays were amplified by incubation at 37 °C with *E. coli* K-12 (ATCC 25404) or *E. coli* BW25113 in Terrific Broth at log phase (OD_600_ of roughly 0.2–0.5), without shaking, for up to 12 h or until lysis. Lysates were passed through a 0.22-μm syringe filter, and the filtrate was stored at 4 °C for subsequent use.

### Phage plaque assays

*E. coli* BW25113 was used in phage plaque assays in Figs. 2a and 3h and Extended Data Figs. 3a,j, 5b and 6e. *E. coli* K-12 (ATCC 25404) was used in phage plaque assays in Extended Data Fig. 10a,b and Supplementary Fig. 17, except for those related to Avs21, for which *E. coli* Stbl3 was used instead owing to the toxicity of Avs21 expression in *E. coli* K-12 (ATCC 25404). *E. coli* cultures were grown to saturation in Terrific Broth at 37 °C with orbital shaking at roughly 220 rpm. To 50 ml of top agar (10 g l^−1^ tryptone, 5 g l^−1^ yeast extract, 10 g l^−1^ NaCl, 7 g l^−1^ agar) was added chloramphenicol (to a final concentration 25 µg ml^−1^) and 1 ml of *E. coli* culture, and the mixture was poured into an empty 15-cm Petri dish and left at room temperature for 10–30 min to solidify. Tenfold serial dilutions of phage in water were then spotted on each plate, with 3 µl per spot. Plates were incubated at 37 °C for roughly 20 h and photographed with a Nikon D7500 DSLR camera in a dark room above a white backlight. Images were scaled, rotated, cropped and converted to grayscale using ImageJ.

### *Se*Avs7 affinity purification

Plasmids encoding N-terminal TwinStrep-tagged *Se*Avs7 or *Se*Avs7_ΔTPR_ (Δ561–1523) under an araBAD promoter were transformed into *E. coli* K-12 (ATCC 25404) cells. A single colony was inoculated in 20 ml of Terrific Broth with carbenicillin (100 μg ml^−1^) and grown at 37 °C for 16 h. The overnight culture was diluted 1:100 into 2 l of fresh Terrific Broth containing carbenicillin (100 μg ml^−1^) and arabinose (0.2% w/v), and incubated at 37 °C with vigorous shaking for 1.5 h. When OD_600_ reached 0.5–0.8, *Escherichia* phage T1 was added at a multiplicity of infection of 2 and cultures were incubated at 37 °C for an extra 30 min without shaking. Cells were collected by centrifugation (4,000 rpm, 10 min, 4 °C).

Cell pellets were resuspended in 5 ml of lysis buffer (50 mM Tris-HCl pH 7.5, 0.5 M NaCl, 5% glycerol, 1 mM ATP, 5 mM MgCl_2_, 1 mM dithiothreitol (DTT)) and lysed by sonication. Insoluble fractions were removed by centrifugation (20,000*g*, 20 min, 4 °C). Clarified lysate was mixed with 100 μl of Strep-Tactin SuperFlow Plus resin (Qiagen), and the resin was washed four times with 1 ml of lysis buffer. Bound proteins were eluted three times with 100 μl elution buffer (0.2 M EPPS pH 8.5, 5 mM desthiobiotin). Eluates were analysed by SDS–PAGE and mass spectrometry (see sections ‘Proteomics sample preparation’, ‘LC–MS analysis’ and ‘Proteomics quantification’).

### Proteomics sample preparation

Pulldown eluates were digested with Lys-C and trypsin (10 ng µl^−1^ each) in 200 mM EPPS pH 8.5 at 37 °C overnight with shaking at 1,200 rpm. Neat acetonitrile was added to each digest to a final concentration of 30% (v/v), and peptides were labelled with 62.5 µg of TMTpro-16 reagent^82^ for 1 h at 25 °C with shaking at 900 rpm. Twenty percent of each tandem mass tag (TMT)-labelled sample was pooled, acidified and diluted with 1% formic acid to pH below 3 and a final acetonitrile concentration less than 5%. The combined sample was desalted with StageTips, dried in a SpeedVac and reconstituted in 5% acetonitrile:5% formic acid.

### LC–MS analysis

A total of 1.3 μg of peptides was analysed by liquid chromatography–mass spectrometry (LC–MS). Peptides were separated on an Aurora Ultimate column 25 cm × 75 μm with 1.5 μm medium and analysed on an Orbitrap Ascend Tribrid mass spectrometer (ThermoFisher) coupled to a Vanquish Neo ultrahigh-performance liquid chromatography apparatus (ThermoFisher) using a 55-min gradient (5–35.6% acetonitrile, 0.125% formic acid) at a 400 nl min^−1^ flow rate. Data-dependent acquisition was used with a mass range of *m/z* 400–1,600. MS1 resolution was set at 120,000, with 50 ms of maximum injection time and a 50% normalized automatic gain control target. The top 10 most abundant species with charge states 2–6 were subjected to fragmentation. MS2 was performed with standard automatic gain control, 35% normalized collisional energy, a 120-ms maximum ion injection time and a 30-s dynamic exclusion window. Fragment ions were further analysed using multi-notch synchronous precursor selection-MS3 (ref. ^83^) with 45% normalized collisional energy to quantify TMT reporter ions.

### Database searching

Database searching was conducted with the Comet algorithm^84^ on Masspike, an in-house search engine described previously in refs. ^84,85^. Searches used a concatenated target–decoy database comprising proteins from *E. coli* ATCC 25404, *Escherichia* phage T1, the *Se*Avs7 expression plasmid, common contaminants (for example, keratins, trypsin) and their reversed sequences. Parameters were 25 ppm precursor mass tolerance, 1.0 Da fragment mass tolerance, fully tryptic specificity (K/R) with up to three missed cleavages and variable Met oxidation (+15.9949 Da). For TMT experiments, TMTpro (+304.2071 Da) on Lys residues and peptide N termini was set as a static modification. Peptide-spectrum matches were filtered by a target–decoy strategy^85–87^ to achieve peptide-level false discovery rate less than 1%, considering XCorr, ΔCn, missed cleavages, peptide length, charge state and precursor mass accuracy. Short peptides (fewer than seven amino acids) were discarded. Proteins were assembled from identified peptides and controlled to protein-level false discovery rate below 1%, and proteins supported by fewer than four unique peptides were discarded.

### Proteomics quantification

For TMT-based quantification, peptide abundance was measured by TMT reporter ions. Reporter intensities were extracted using a 0.003-Th window centred on the theoretical *m/z*, selecting the most intense peak. Isotopic impurities were corrected based on the manufacturer’s specifications, and TMT signal-to-noise values were used for quantification. Peptides were discarded if the summed signal-to-noise across 6 channels of each TMT plex was less than 60 or if isolation specificity was less than 50%. Protein abundances were computed by summing peptide signal-to-noise values. Protein quantification was normalized to ensure equal *Se*Avs7 protein loading across all TMT channels. *Se*Avs7 abundance was quantified using peptides mapping to the N-terminal 596 amino acids, including the TwinStrep tag.

### Phage genome sequencing

The genomes of all phages isolated from the City of Palo Alto were sequenced using Tn5 tagmentation and paired-end Illumina sequencing, as previously described in ref. ^4^. Adapter sequences were trimmed from sequencing reads using CutAdapt with parameters–trim-n -q 20 -m 20 -a CTGTCTCTTATACACATCT -A CTGTCTCTTATACACATCT. Trimmed reads were assembled into contigs with SPAdes v.4.2.0 (ref. ^88^) using the –careful option or unicycler v.0.5.1 (ref. ^89^) with the default option. Genes were annotated by Prokka^90^, Pharokka^91^, Phold^92^ or by comparison with a reference genome of a highly similar phage in GenBank. Isolated phages (Pal001–014) were classified according to the rules of the International Committee on the Taxonomy of Viruses (ICTV)^93,94^. The identities of core structural and replicative genes were confirmed on the basis of HHpred^77^ predictions. The phage phylogeny in Extended Data Fig. 6f was generated by concatenating alignments of the large terminases and MCPs. Phage genomes in the same figure were visualized using pyGenomeViz.

### Phage gene library construction

To construct each library, barcoded plasmids containing phage genes were pooled along with 8–12 uniquely barcoded mNeonGreen clones as internal normalization controls. The amount of each plasmid in the library was adjusted to account for variations in *E. coli* growth rates due to phage gene expression, to ensure balanced plasmid levels across the library after growth in *E. coli*. To determine these adjustments, an initial pilot pool of plasmids, mixed in equal volumes, was transformed into *E. coli* NovaBlue(DE3) (Millipore Sigma) containing an empty pACYC184-like vector. These cells were grown for 12 h at 37 °C on minimal salt Luria-Bertani (LB) agar (10 g l^−1^ tryptone, 5 g l^−1^ yeast extract, 0.5 g l^−1^ NaCl, 7 g l^−1^ agar) containing 100 µg ml^−1^ carbenicillin, 25 µg ml^−1^ chloramphenicol and 0.005% arabinose. The relative barcode abundances from plasmids isolated from surviving colonies were quantified by deep sequencing. On the basis of these abundances, the volume of each plasmid added to the final library was adjusted accordingly.

### Competent cell production

*E. coli* strains were cultured in ZymoBroth with 25 µg ml^−1^ chloramphenicol and made competent using a Mix and Go Transformation Kit (Zymo) according to the manufacturer’s recommended protocol.

### Co-expression toxicity screens

Co-expression screens for all Avs systems were performed in *E. coli* NovaBlue(DE3), with the exception of Avs21, which was performed in *E. coli* Stbl3 instead due to its toxicity in *E. coli* NovaBlue(DE3). *E. coli* strains containing Avs plasmids were made competent and transformed with 50 ng of the phage gene library, followed by 30 min of incubation on ice, 1 min of heat shock at 42 °C and 1 h of outgrowth at 37 °C in super optimal broth with catabolite repression. Transformed cells were grown on LB agar plates containing 25 μg ml^−1^ chloramphenicol, 100 μg ml^−1^ carbenicillin and 0.01% arabinose. Cells expressing Avs2, Avs12, Avs18 and Avs21 were grown on LB agar Lennox (10 g l^−1^ tryptone, 5 g l^−1^ yeast extract, 5 g l^−1^ NaCl, 7 g l^−1^ agar), while cells expressing other Avs families were grown on LB agar with minimal salt (10 g l^−1^ tryptone, 5 g l^−1^ yeast extract, 0.5 g l^−1^ NaCl, 7 g l^−1^ agar). For each sample, a corresponding empty vector control was grown in parallel using the same strain and medium. Plates were incubated for 12 h at 37 °C, and plasmids from surviving colonies were isolated by miniprep (Qiagen). Barcodes were amplified using Q5 polymerase over eight cycles of PCR with primers containing Illumina sequencing adaptors and sample-specific 8-nt indexes. Purified amplicons were sequenced on a NextSeq 550 (Illumina) with 20 cycles for Read 1. Barcode counts for each plasmid were determined from demultiplexed FASTQ files, requiring a perfect 20-bp match.

For each sample, barcode counts were normalized by dividing them by the average of the mNeonGreen counts within that sample. For each barcode, synthetic toxicity was then quantified as *q* = (normalized count in Avs sample)/(normalized count in the empty vector (no-Avs) control). For phage genes represented by many barcodes, *q* values were averaged across all barcodes for that gene to obtain a single *q* value. Fold depletion was calculated as 1/*q*.

### Anion exchange chromatography

Anion exchange chromatography was performed using an ÄKTA Explorer (Cytiva) equipped with a HiTrap Q HP column (Cytiva). A linear NaCl gradient (0–1 M) in presence of 20 mM Tris-HCl pH 7.5 was applied to purify proteins. Fractions were monitored by ultraviolet (UV) absorbance at 214 nm, 260 nm and 280 nm, and by SDS–PAGE. Desired fractions were concentrated using Amicon Ultra centrifugal filters (Millipore).

### SEC

SEC was performed using an ÄKTA Explorer (Cytiva) equipped with a Superose 6 10/300 GL column (Cytiva). The column was pre-equilibrated with either an ATP-containing SEC buffer (20 mM Tris-HCl pH 7.5, 0.2 M NaCl, 0.1 mM ATP and 2 mM MgCl_2_) or an ATP-free SEC buffer (20 mM Tris-HCl pH 7.5, 0.2 M NaCl). Fractions were monitored by UV absorbance at 214 nm, 260 nm and 280 nm and SDS–PAGE. Desired fractions were concentrated using Amicon Ultra centrifugal filters (Millipore).

### Protein purification

Plasmids encoding ZL19 MCP-TwinStrep (TS), TS-SUMO-*Se*Avs7 variants, TS-*Ec*EF-Tu-His_6_, TS-SUMO-*Mv*Avs13, TS-SUMO-PhiV-1 tail nozzle, TS-SUMO-*Kp*Avs20 or TS-SUMO-HdK1 RecA were transformed into *E. coli* Rosetta (DE3) (Fisher Scientific) or BL21 (DE3) (New England Biolabs). Transformed cells were plated on LB agar and incubated overnight at 37 °C. Single colonies were inoculated into Terrific Broth with 100 μg ml^−1^ carbenicillin and grown overnight at 37 °C. Starter cultures were diluted 1:100 into fresh Terrific Broth and grown at 37 °C to an OD_600_ of 0.8. Protein expression was induced with 0.05–0.1 mM isopropyl-d-1-thiogalactopyranoside and cultures were incubated at 22 °C for 16 h with roughly 220 rpm orbital shaking. Cells were collected by centrifugation and pellets were stored at −80 °C.

To purify proteins other than *Ec*EF-Tu, pellets were resuspended in lysis buffer (50 mM Tris-HCl pH 7.5, 500 mM NaCl, 5% glycerol (v/v), 1 mM DTT, 1 mM ATP, 5 mM MgCl_2_, 1× cOmplete Protease Inhibitor (Sigma Aldrich)) and lysed using an LM20 Microfluidizer (Microfluidics) at 28,000 PSI. Lysates were clarified by centrifugation at 32,000*g* for 30 min at 4 °C, and the supernatant was incubated with Strep-Tactin SuperFlow Plus resin for 1 h at 4 °C. Resin-bound proteins were washed three times with the lysis buffer on a Poly-Prep chromatography column (Bio-Rad). ZL19 MCP was eluted with a buffer containing 20 mM Tris-HCl pH 7.5, 5 mM desthiobiotin. For all other proteins, bdSENP1 protease was added for on-resin cleavage and incubated overnight at 4 °C, and proteins were eluted with 20 mM Tris-HCl pH 7.5. Proteins were further purified by anion exchange chromatography, and buffers were exchanged to 20 mM Tris-HCl pH 7.5, 0.2 M NaCl using Amicon Ultra centrifugal filters (Millipore). For nuclease activity assays, proteins were also polished by SEC in ATP-free SEC buffer.

For TS-*Ec*EF-Tu–His_6_, pellets were resuspended in His-lysis buffer (50 mM Tris-HCl pH 7.5, 0.3 M NaCl, 40 mM imidazole, 5% glycerol (v/v), 1× cOmplete protease inhibitor) and lysed by microfluidizer. Clarified lysate was incubated with Ni-NTA SuperFlow resin (Qiagen), and resin-bound protein was washed three times with His-lysis buffer and eluted with His-elution buffer (50 mM Tris-HCl pH 7.5, 0.1 M NaCl, 500 mM imidazole, 5% glycerol). The eluates were incubated with Strep-Tactin SuperFlow Plus resin, which was then washed three times with lysis buffer and eluted with 5 mM desthiobiotin. The protein was further purified by anion exchange chromatography. Final polishing was performed by SEC using a HiLoad 16/600 Superdex 200 pg column (Cytiva) pre-equilibrated with ATP-free SEC buffer. Protein concentration was determined by UV absorbance at 280 nm.

### In vitro DNA cleavage assay

*Se*Avs7 (0 or 320 nM), ZL19 MCP-TS (1.6 μM or as specified) and TS-*Ec*EF-Tu–His_6_ (10 μM or as specified) were incubated in a reaction buffer containing 20 mM Tris-HCl (pH 7.5), 0.2 M NaCl, 1 mM ATP (or ADP or AMP-PNP as specified) and 5 mM MgCl_2_ for 15 min at room temperature. DNA was added at a final concentration of 5 ng μl^−1^, and the reaction mixture was incubated at room temperature for 15 min or for the indicated times. DNA products were purified using QIAquick PCR purification columns (Qiagen) and analysed by gel electrophoresis on an E-Gel EX 2% agarose gel (ThermoFisher). Gel images were acquired using a Bio-Rad VersaDoc imaging system, and the signal intensity for each lane was quantified with the ImageJ Plot Profile tool.

### Complex formation and purification

For the *Se*Avs7 variant–ZL19 MCP complex, *Se*Avs7 variant (5 μM; 1 eq.) was incubated with ZL19 MCP (10 μM; 2 eq.) and *Ec*EF-Tu (0 or 10 μM; 0 eq. or 2 eq.) in a buffer containing 20 mM Tris-HCl (pH 7.5), 0.2 M NaCl, 1 mM ATP, 5 mM MgCl_2_ and 1 mM DTT for 1 h at room temperature. For the other Avs–phage trigger pairs, Avs protein (10 μM; 1 eq.) was incubated with the cognate phage protein (10 μM; 2 eq.) in the same buffer for 30 min at room temperature. Complexes were purified by SEC using an ATP-containing SEC buffer.

### Cryo-EM sample preparation and data preparation

To assemble the *Se*Avs7 complex, Strep-Tactin-purified *Se*Avs7 (5 µM) was incubated with Strep-Tactin-purified ZL19 MCP-TS (10 µM) in 50 mM Tris-HCl (pH 7.5), 0.5 M NaCl, 1 mM ATP, 5 mM MgCl_2_, 1 mM DTT and 5% glycerol (v/v) for 1 h at room temperature. The resulting complex was purified by SEC using a Superose 6 10/300 Increase GL column equilibrated with SEC buffer containing 20 mM Tris-HCl (pH 7.5), 0.2 M NaCl, 0.1 mM ATP and 2 mM MgCl_2_. For apo *Se*Avs7, Strep-Tactin-purified *Se*Avs7 was directly separated by SEC under the same conditions. The ATP-free SEC buffer was also used for comparison in Supplementary Fig. 6b.

To prepare cryo-EM grids, 3 μl of *Ec*EF-Tu-bound apo *Se*Avs7 (5 mg ml^−1^) or the *Se*Avs7–ZL19 MCP–*Ec*EF-Tu complex (3 mg ml^−1^) was applied to freshly glow-discharged 300-mesh Quantifoil R1.2/1.3 holey carbon grids. The grids were vitrified in liquid ethane using a Vitrobot Mark IV (ThermoFisher) at 4 °C and 100% humidity, with blotting conditions set to a wait time of 5 s, a blot time of 3 s and a blot force of 3. Cryo-EM data were collected on a 300 kV Titan Krios (S2C2 at Stanford or cEMc at Stanford) equipped with a Falcon 4 detector (ThermoFisher) using EPU software in counting mode. For apo *Se*Avs7, images were acquired at a nominal magnification of ×130,000, corresponding to a physical pixel size of 0.946 Å. For the *Se*Avs7–ZL19 MCP–*Ec*EF-Tu complex, images were collected at the same magnification, with a pixel size of 0.95 Å. A total of 40 frames per movie stack were recorded, each with a total electron dose of 50 electrons per Å^2^.

### Cryo-EM data processing

For the *Se*Avs7–ZL19 MCP–*Ec*EF-Tu complex, 5,805 videos were processed using cryoSPARC live v.4.4 (ref. ^95^), including Patch motion correction, Patch contrast transfer function (CTF) estimation, template picking and particle extraction with a box of 120 pixels and 4 × 4 binning. A total of 5,704 images with a CTF resolution better than 8 Å were selected, resulting in 3,769,341 particles. After 2 rounds of two-dimensional (2D) classification, 487,281 particles were selected for ab initio reconstruction to generate initial models. Following 2 rounds of heterogeneous refinement, 312,068 particles (in two groups containing 205,105 and 106,963 particles) were re-extracted with a box size of 480 pixels. The 205,105 particles in the first group were subjected to 1 more round of heterogeneous refinement, after which 78,090 particles were selected for non-uniform refinement, yielding a 3.43 Å resolution map of the tetrameric complex (Map A). To improve the resolution of the four *Se*Avs7 TPR–ZL19 MCP–*Ec*EF-Tu interfaces, local refinement was performed individually, yielding improved resolutions of 4.01 Å (Map B), 3.78 Å (Map C), 3.77 Å (Map D) and 3.70 Å (Map E), respectively. A composite map (Map F) was generated by combining Maps A–E in Phenix. As *Se*Avs7 TPR–ZL19 MCP–*Ec*EF-Tu domains were structurally similar, all particles were aligned using the volume alignment tool, resulting in 312,068 particles subjected to further three-dimensional (3D) classification, yielding 59,867 particles. These domains were consistent with those of the *Se*Avs7–ZL19 MCP–*Ec*EF-Tu monomeric complex from the second particle group (with 106,963 particles). Therefore, these particles were combined with the second particle group, resulting in a total of 166,830 particles. These underwent further 3D classification and local refinement, producing a final 3.40 Å map (Map G). Maps A–E were sharpened by Local resolution filter, and local resolution was estimated using BlocRes.

For the apo *Se*Avs7 dataset, 1,209 videos were initially motion corrected using MotionCor2 (ref. ^96^). The dose-weighted images were imported into cryoSPARC v.4.4 (ref. ^95^), in which the CTF was estimated using Patch CTF estimation. Template-based particle picking identified 800,233 particles with a box size of 80 pixels and 4 × 4 binning. After one round of 2D classification, 304,831 particles were selected for ab initio reconstruction, yielding three initial models for subsequent heterogeneous refinement. The entire set of 800,233 particles was subjected to 2 rounds of heterogeneous refinement, resulting in 207,066 particles. This subset was re-extracted with a box of 320 pixels and further refined using non-uniform refinement, producing a final map at 2.72 Å resolution. Local resolution was estimated using BlocRes, and the map was sharpened using Local resolution filter.

### Model building and refinement

Initial models for all structures were generated using AlphaFold3 predictions and subsequently fitted into the cryo-EM map using Chimera. Iterative manual model building was performed using Coot^97^. The adjusted models were then refined using Phenix^98^ to optimize geometry and stereochemistry. In addition, refinement was performed using e2gmm_model_refine.py^99^ in EMAN2 (ref. ^100^) to achieve improved Protein Data Bank (PDB) validation scores. Model quality was assessed by MolProbity. Structures were visualized using PyMOL (Schrödinger) and ChimeraX^101^.

### Structural analyses

Structural models for proteins and complexes were generated with AlphaFold3 or with AlphaFold multimer in ColabFold^102^, requiring a minimum interface predicted template modelling (ipTM) score of 0.7 for Avs complexes. Structures were visualized with PyMOL (Schrödinger) or ChimeraX v.1.8 (UCSF). Structure-guided alignments of homologous phage target proteins were obtained with PROMALS3D^44^, using AlphaFold3 models as inputs. Alignable regions, corresponding to the core folds of the target proteins, were used to determine pairwise sequence identity.

To assess the generalizability of Avs7–MCP–EF-Tu complex formation across Avs7 homologues, structures were predicted as a five-component complex (one each of XxAvs7, XxEF-Tu, ZL19 MCP, ATP and Mg^2+^, in which Xx represents distinct bacterial species), with AlphaFold3. Structures were clustered along with the cryo-EM structure of the *Se*Avs7–ZL19 MCP–*Ec*EF-Tu monomeric complex using Foldseek multimer-cluster^103^ at 60% coverage and a TM score of 0.70 (Supplementary Table 11). Structures that clustered with the cryo-EM reference are shown in Extended Data Fig. 6.

### Statistics and reproducibility

Each assay was repeated two or three times with similar results. No statistical methods were used to predetermine sample size.

### Reporting summary

Further information on research design is available in the Nature Portfolio Reporting Summary linked to this article.

## Online content

Any methods, additional references, Nature Portfolio reporting summaries, source data, extended data, supplementary information, acknowledgements, peer review information; details of author contributions and competing interests; and statements of data and code availability are available at https://doi.org/10.1038/s41586-026-10852-6.

## Supplementary information

Supplementary Information Supplementary Figs. 1–19.

Reporting Summary

Supplementary Tables Supplementary Tables 1–13.

Supplementary Data Supplementary Data 1. MSA of the 359,879 representative STANDs across 589 clades. Supplementary Data 2. Phylogenetic tree of the 359,879 representative STANDs across 589 clades. Supplementary Data 3. AlphaFold models of Avs proteins and their corresponding phage triggers.

Supplementary Data Source data for Supplementary Figs. 7, 8, 13 and 14.

## Source data

Source Data Fig. 2 and Extended Data Fig. 8

Source Data Fig. 3

Source Data Fig. 5

Source Data Fig. 6

Source Data Extended Data Fig. 3

## Figures and Tables

**Extended Data Table 1:**
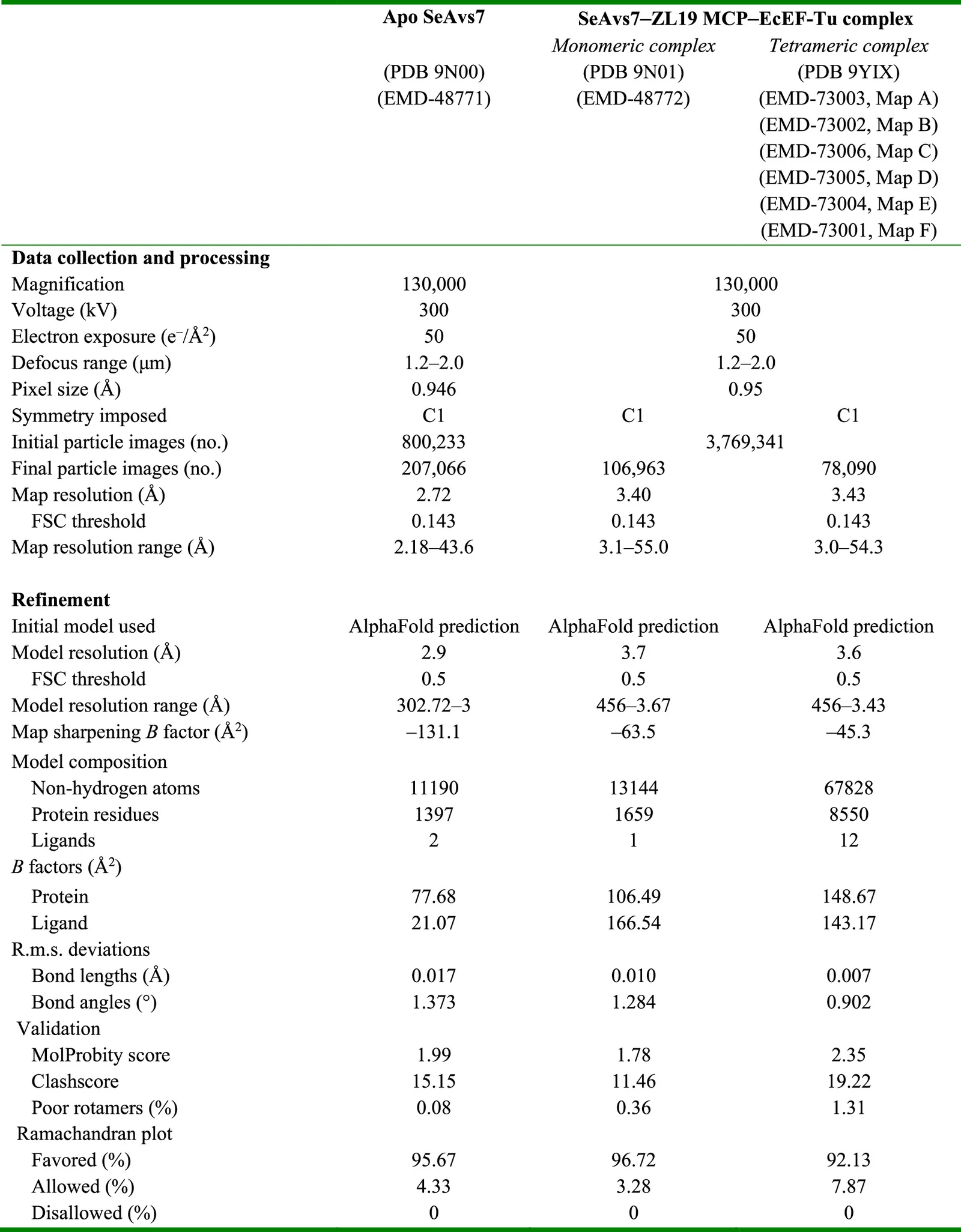
Cryo-EM data collection, refinement, and validation statistics

## Data Availability

The genome sequences of phages Pal001–013 have been deposited in GenBank under the accession numbers PX985120–PX985132. Protein sequences and taxonomic information are publicly available from the NCBI Protein (https://www.ncbi.nlm.nih.gov/protein/) and Taxonomy (https://www.ncbi.nlm.nih.gov/taxonomy) databases. Mass spectrometric data have been deposited in Proteomics Identification Database under accession code PXD079457. Cryo-EM maps have been deposited in the Electron Microscopy Data Bank under accession numbers EMD-73001 (Map F; composite map of the *Se*Avs7–ZL19 MCP–*Ec*EF-Tu tetrameric complex), EMD-73002 (Map B), EMD-73003 (Map A), EMD-73004 (Map E), EMD-73005 (Map D), EMD-73006 (Map C), EMD-48771 (Apo *Se*Avs7) and EMD-48772 (Map G; *Se*Avs7–ZL19 MCP–*Ec*EF-Tu monomeric complex). The corresponding atomic models have been deposited in the PDB under accession codes 9YIX (Map F), 9N01 (Map G) and 9N00 (Apo *Se*Avs7). Previously published structures rendered in this study are publicly available in the PDB, with accession codes provided in the corresponding figures. Source data are provided with this paper.
