# Supplementary material for: Diverse bacterial pattern recognition receptors sense the core phage proteome
Source: Nature. 2026 Jul 29;657(8132):735–43. doi: 10.1038/s41586-026-10852-6 (PMC13581612; doi:10.1038/s41586-026-10852-6)
Supplement: Supplementary file 1 [file 41586_2026_10852_MOESM1_ESM.pdf]

nature portfolio

https://doi.org/10.1038/s41586-026-10852-6

Supplementary information

Diverse bacterial pattern recognition receptors sense the core phage proteome

In the format provided by the authors and unedited

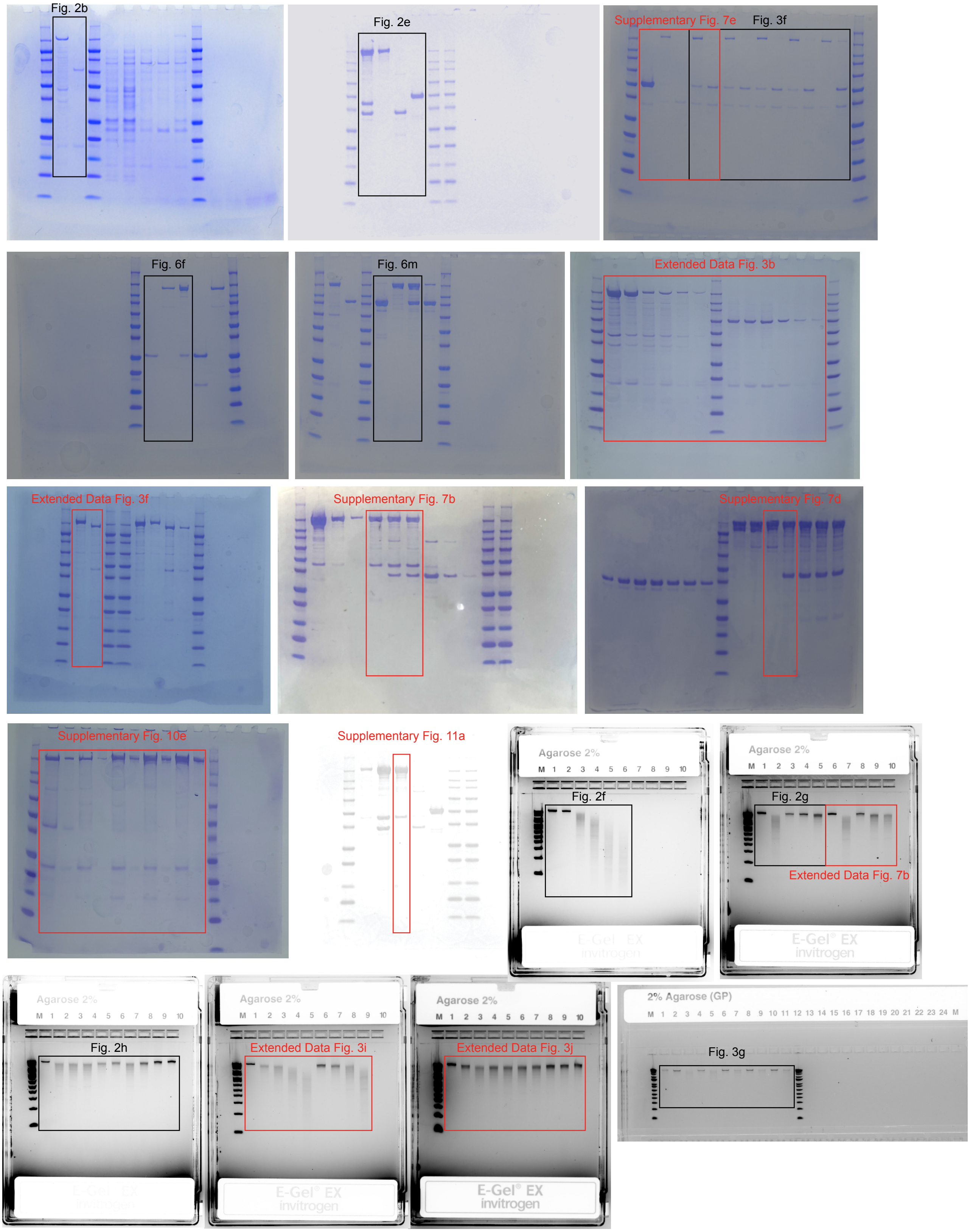

Supplementary Fig. 1 | Uncropped SDS-PAGE and DNA agarose gel images. Boxes indicate the cropped regions.

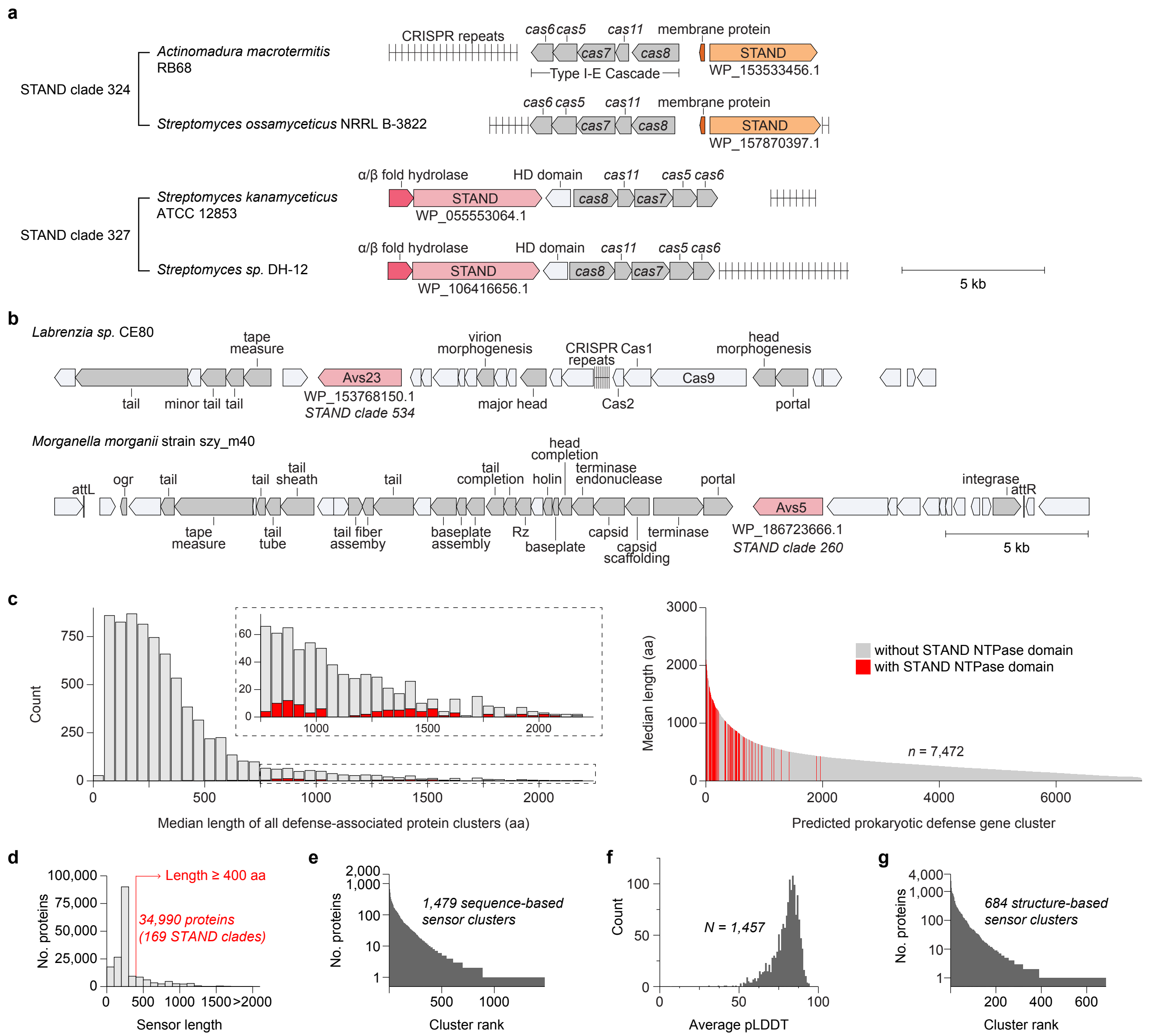

Supplementary Fig. 2 | Related to Fig. 1. a, Representative genomic loci of CRISPR-associated STANDs of unknown function in Actinomycetota. **b,** Representative genomic loci of prophage-encoded Avs5 and Avs23. **c,** Median lengths of 7,472 defense-associated gene clusters predicted previously (Gao et al. *Science* **369**, 1077–1084 (2020)), with STAND NTPases highlighted in red. **d,** Histogram of the sensor domain lengths for proteins within defense-associated STAND clades (n = 178,614). **e,** Number of proteins within each of the 1,479 sequence-based sensor clusters. **f,** Distribution of average pLDDT scores for the AlphaFold models of 1,457 sensors. **g,** Number of proteins within each of the 684 structure-based sensor clusters.

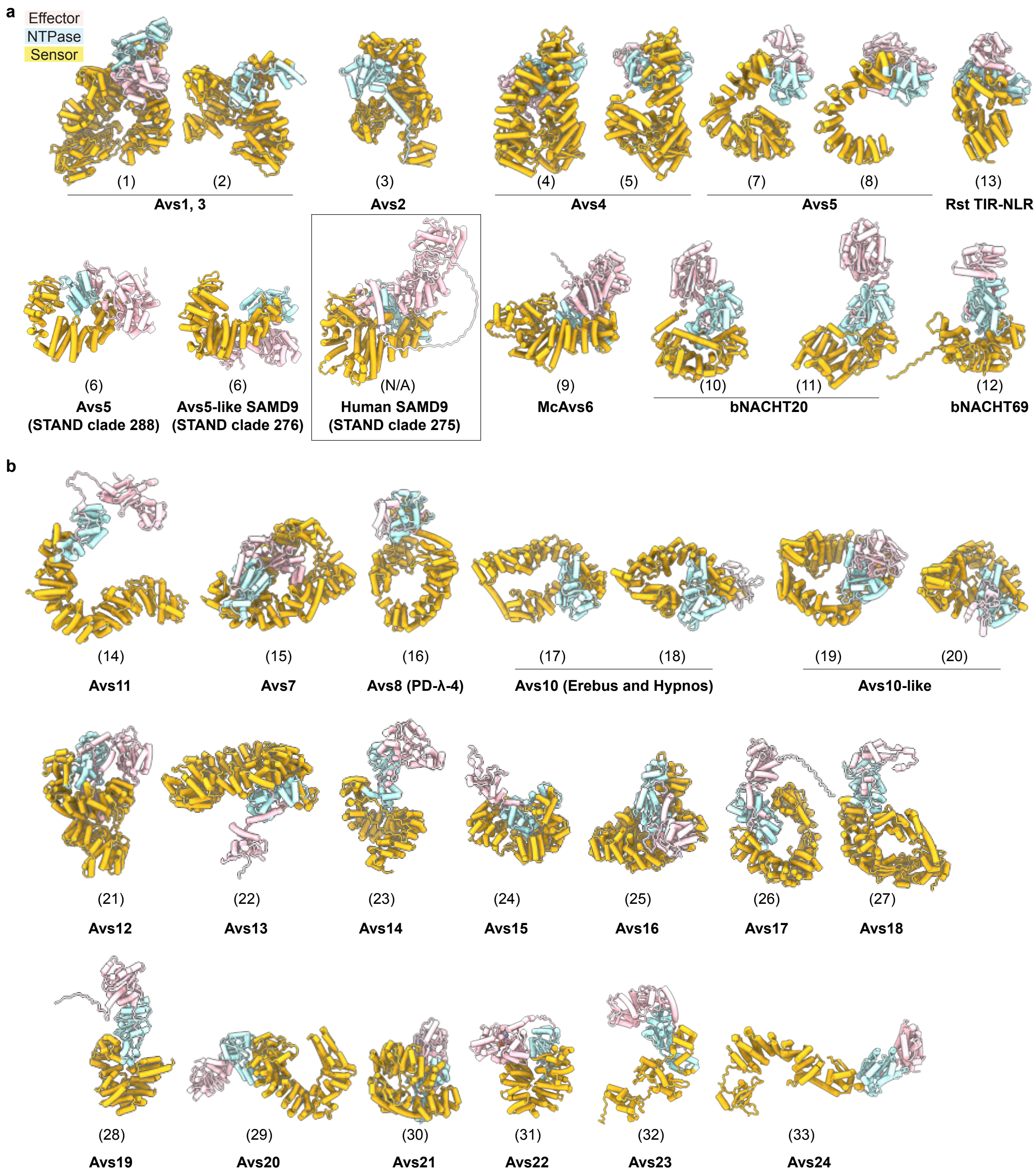

Supplementary Fig. 3 | Related to Fig. 1. a–b, AlphaFold3 models of representatives of structural clusters that were previously characterized (**a**) or investigated in the current study (**b**). Sensor structural cluster numbers are shown in parentheses. Human SAMD9 is shown in the inset for comparison.

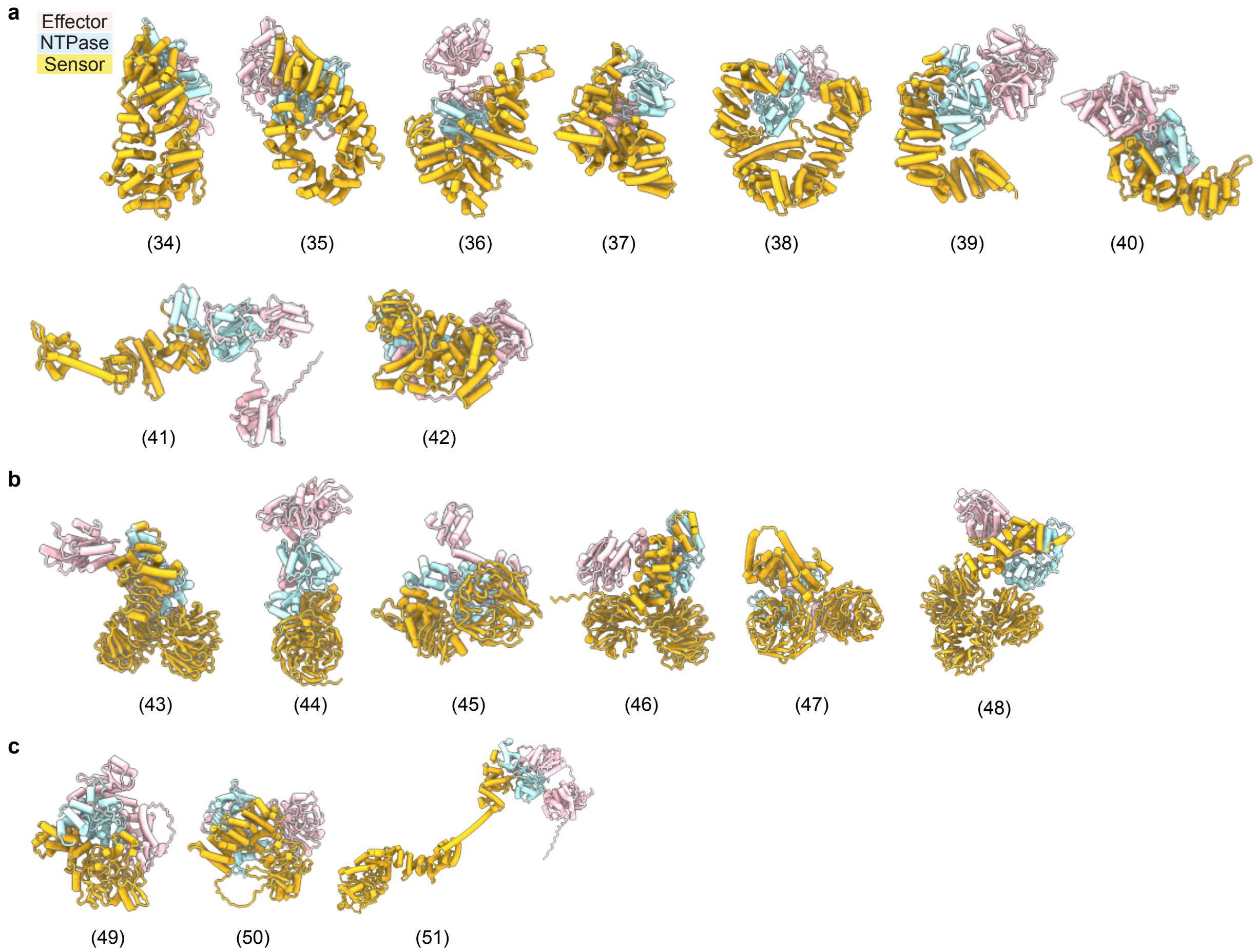

Supplementary Fig. 4 | Related to Fig. 1. a, AlphaFold3 models of representative sensors in *Enterobacteriaceae*. **b–c**, AlphaFold3 models of representative sensors containing a WD40 domain (**b**) or a formylglycine-generating enzyme (FGE) domain (**c**). Sensor structural cluster numbers are shown in parentheses.

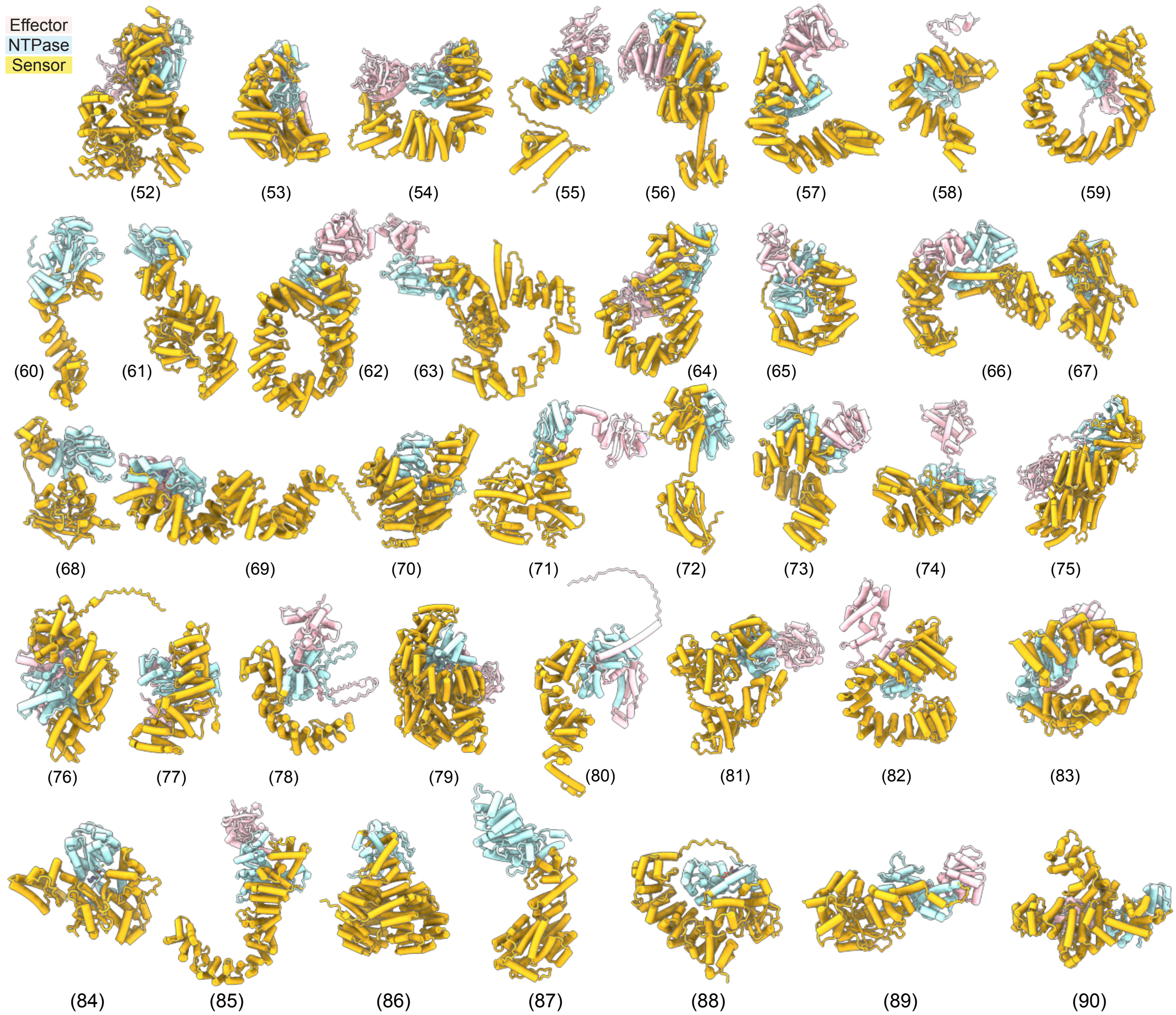

Supplementary Fig. 5 | Related to Fig. 1. AlphaFold3 models of 39 additional representative sensors. Sensor structural cluster numbers are shown in parentheses.

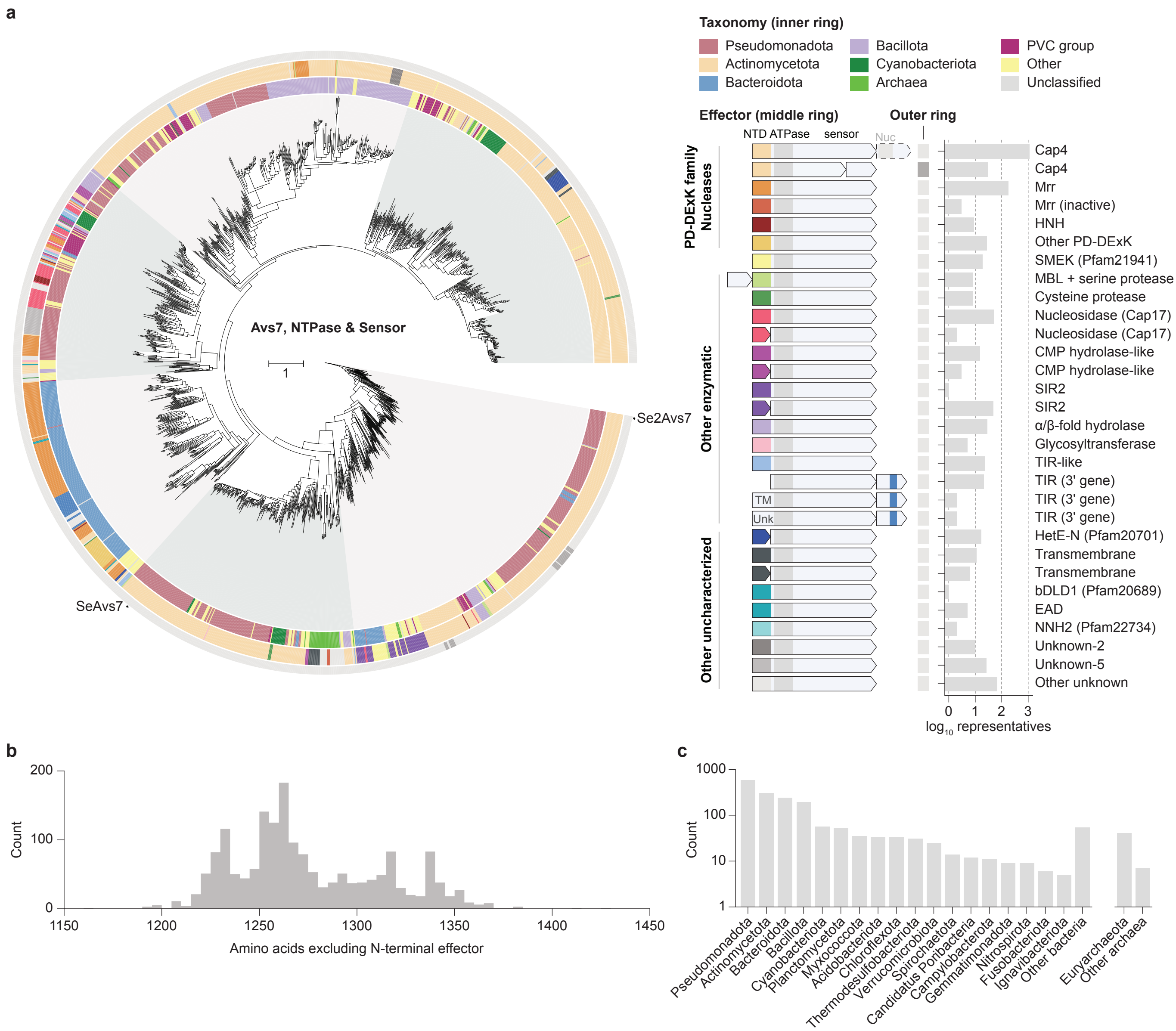

Supplementary Fig. 6 | Prevalence and diversity of Avs7 homologs. a, Maximum likelihood tree based on the NTPase and C-terminal domains of 1,757 representative Avs7 homologs. The inner ring indicates taxonomy, and the middle and outer rings represent the effector and sensor domains, respectively. MBL, metallo-β-lactamase; EAD, effector-associated domain; TM, transmembrane domain; Unk, unknown. **b**, Histogram of the combined length of the NTPase and C-terminal domains across Avs7 homologs. **c**, Abundance of Avs7 homologs across bacterial and archaeal phyla.

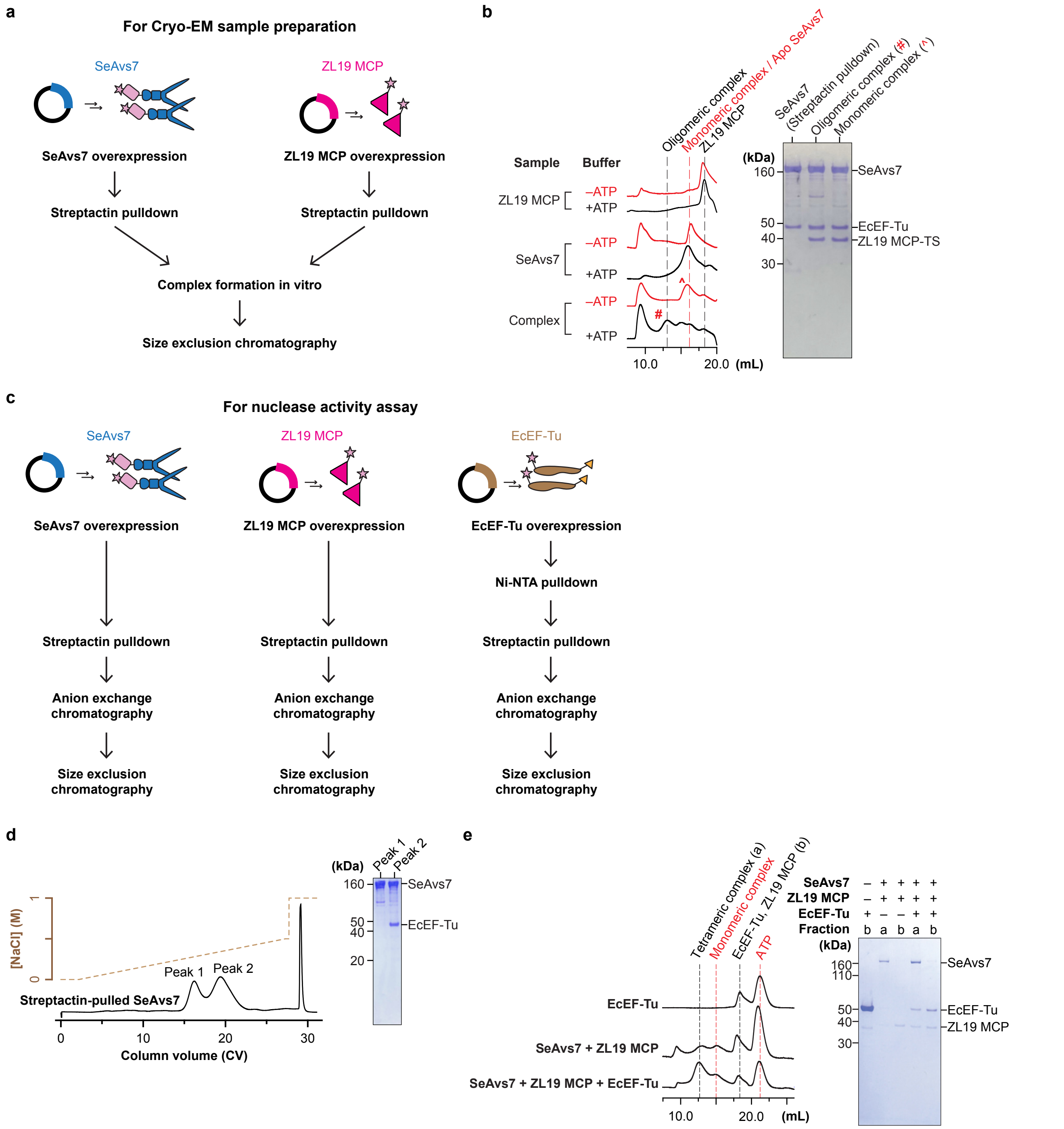

Supplementary Fig. 7 | SeAvs7 binds ZL19 MCP and EcEF-Tu independently of ATP. **a**, Schematic of the SeAvs7–ZL19 MCP complex purification workflow for structural analysis. **b**, Size-exclusion chromatograms of Strep-Tactin-purified SeAvs7, ZL19 MCP, and their *in vitro* assembled complex, with SDS-PAGE analysis of selected fractions. Dashed lines indicate expected retention volumes. **c**, Schematic of the SeAvs7, ZL19 MCP, and EcEF-Tu purification workflow for *in vitro* nuclease assays. **d**, Anion-exchange chromatograms of purified SeAvs7 and SDS-PAGE analysis of two peaks. **e**, Size-exclusion chromatograms and SDS-PAGE analysis of complex formation with purified proteins used for nuclease assays. Data for SeAvs7 + ZL19 MCP + EcEF-Tu are reproduced from Fig. 3f. For gel source data, see Supplementary Fig. 1.

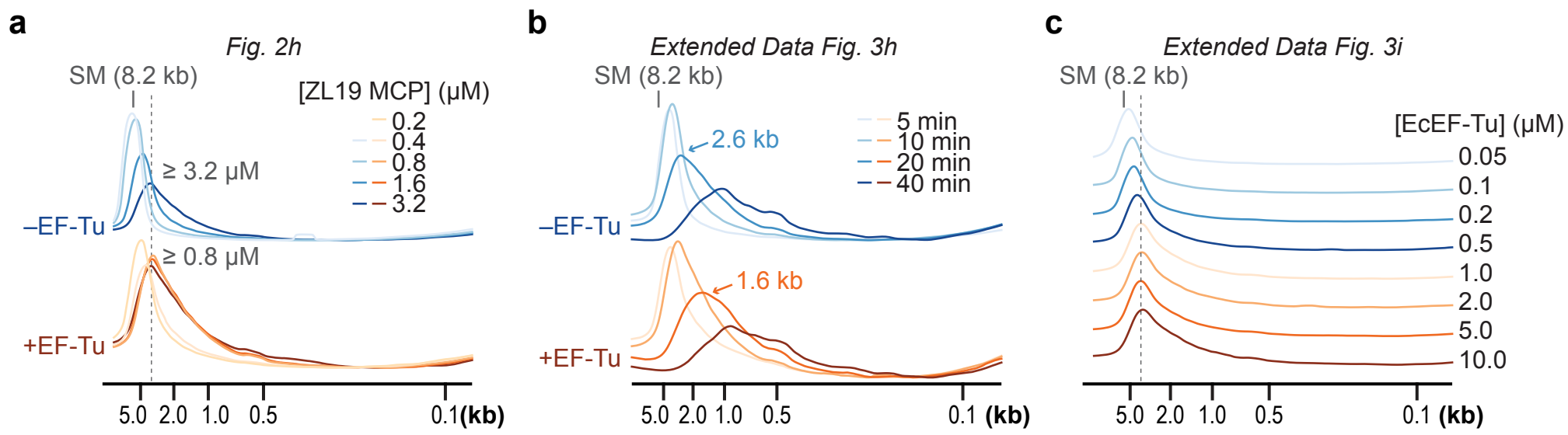

Supplementary Fig. 8 | Quantification of in vitro cleavage kinetics. Densitometric quantification of substrate DNA degradation from raw agarose gels. The most frequent fragment lengths (**a**) or the maximum fragment shift (**b, c**) observed are indicated. SM, substrate double-stranded DNA (8.2 kb).

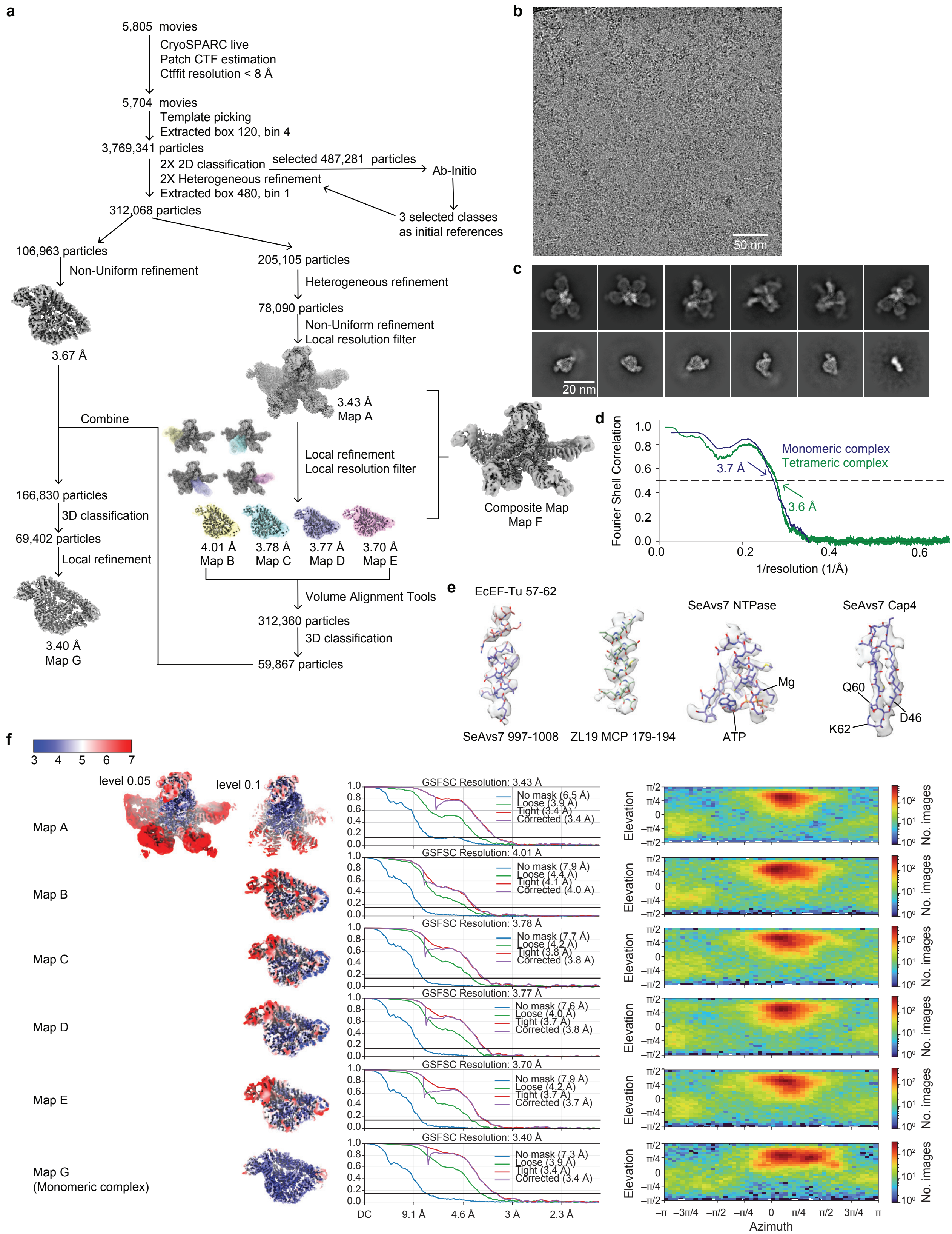

Supplementary Fig. 9 | Cryo-EM data processing and validation for the SeAvs7–ZL19 MCP–EcEF-Tu complex. a, Data processing workflow. **b**, Representative cryo-EM micrograph. **c**, Representative 2D class averages. **d**, Map-to-model Fourier shell correlation (FSC) curve. **e**, Representative cryo-EM density from the composite map overlaid with the atomic model. **f**, Quality assessment metrics, including local resolution maps (left), gold-standard FSC curves (middle), and angular particle distributions (right).

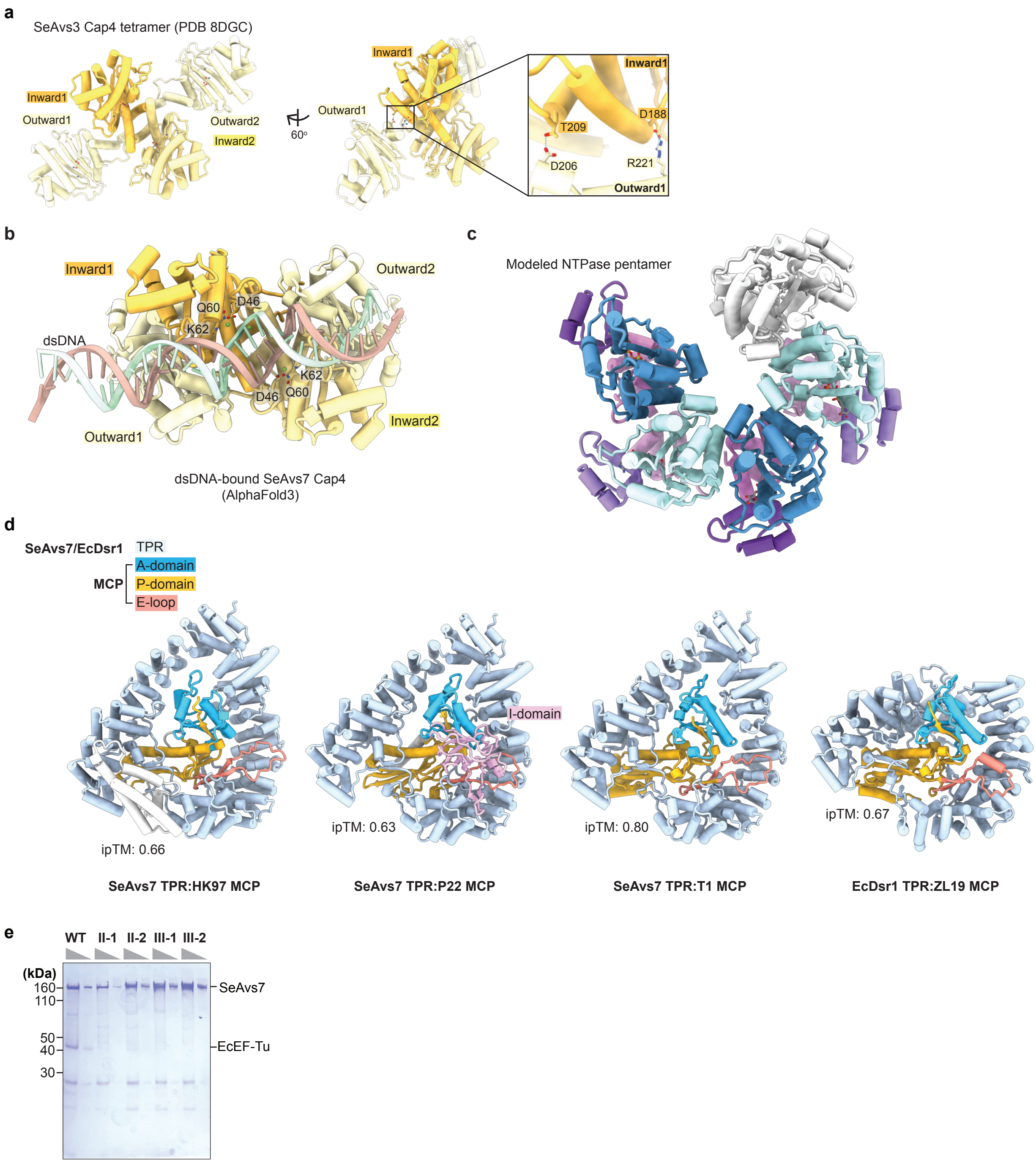

Supplementary Fig. 10 | Related to Fig. 3. a, Structures of the Cap4 tetramers of SeAvs3 (PDB 8DGC) (Gao et al. *Science* **377**, eabm4096 (2022)). The inset highlights interactions between the inward-facing and outward-facing protomers. **b**, AlphaFold3 model of the SeAvs7 Cap4 tetramer bound to a double-stranded DNA substrate and four Mg^{2+} ions. Interface predicted template modeling (ipTM) scores between the DNA strands and Cap4 monomers range from 0.54 to 0.60. **c**, A model of an SeAvs7 NTPase domain pentamer generated from a structural alignment to the NTPase tetramer. **d**, AlphaFold3 models of the SeAvs7 and EcDsr1 TPR–MCP complexes, with ipTM scores indicated. **e**, SDS-PAGE analysis of wild-type and mutant SeAvs7 purified from *E. coli*. See Supplementary Fig. 1 for gel source data.

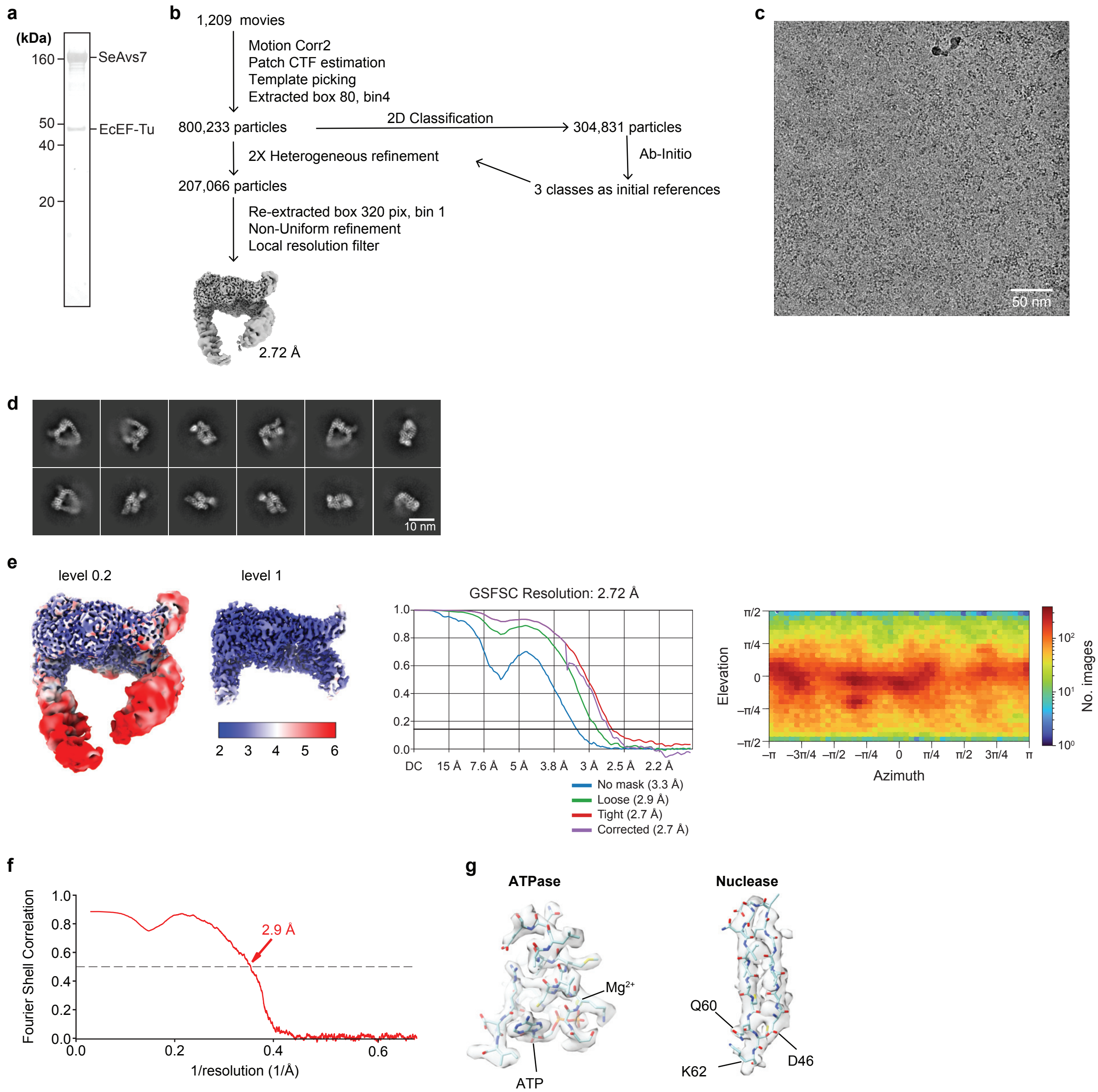

Supplementary Fig. 11 | Cryo-EM data processing and validation for apo SeAvs7. a, SDS-PAGE analysis of SeAvs7 co-purified with endogenous EcEF-Tu for cryo-EM grid preparation. See Supplementary Fig. 1 for source data. **b**, Data processing workflow. **c**, Representative cryo-EM micrograph. **d**, Representative 2D class averages. **e**, Quality assessment metrics, including the local resolution map (left), gold-standard FSC curve (middle), and angular particle distribution (right). **f**, Map-to-model FSC curve. **g**, Representative cryo-EM density overlaid with the atomic model.

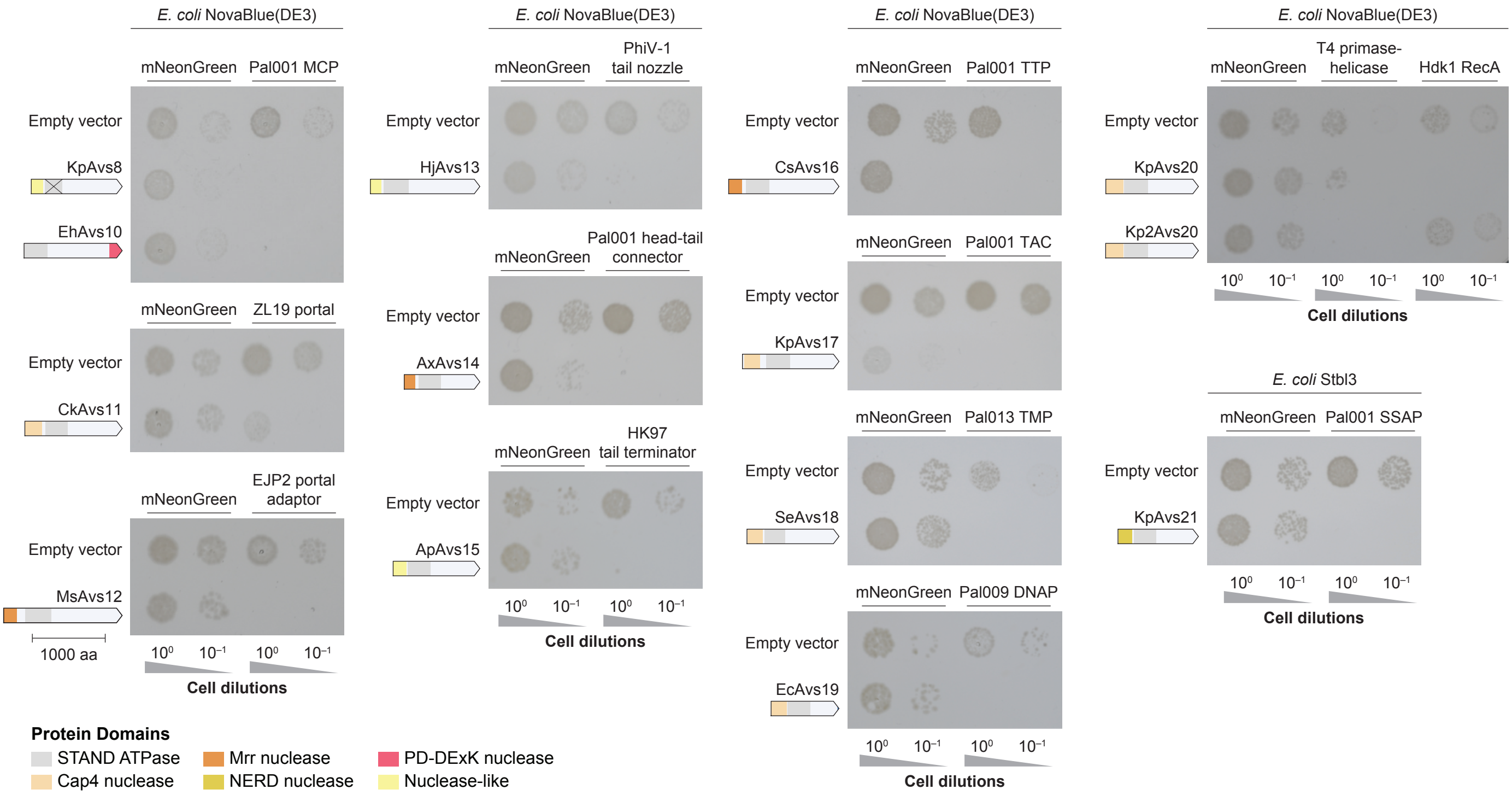

Supplementary Fig. 12 | Co-expression of Avs–trigger pairs induces toxicity in *E. coli*. Representative photographs of *E. coli* co-transformation assays with Avs proteins and phage triggers identified in Fig. 5.

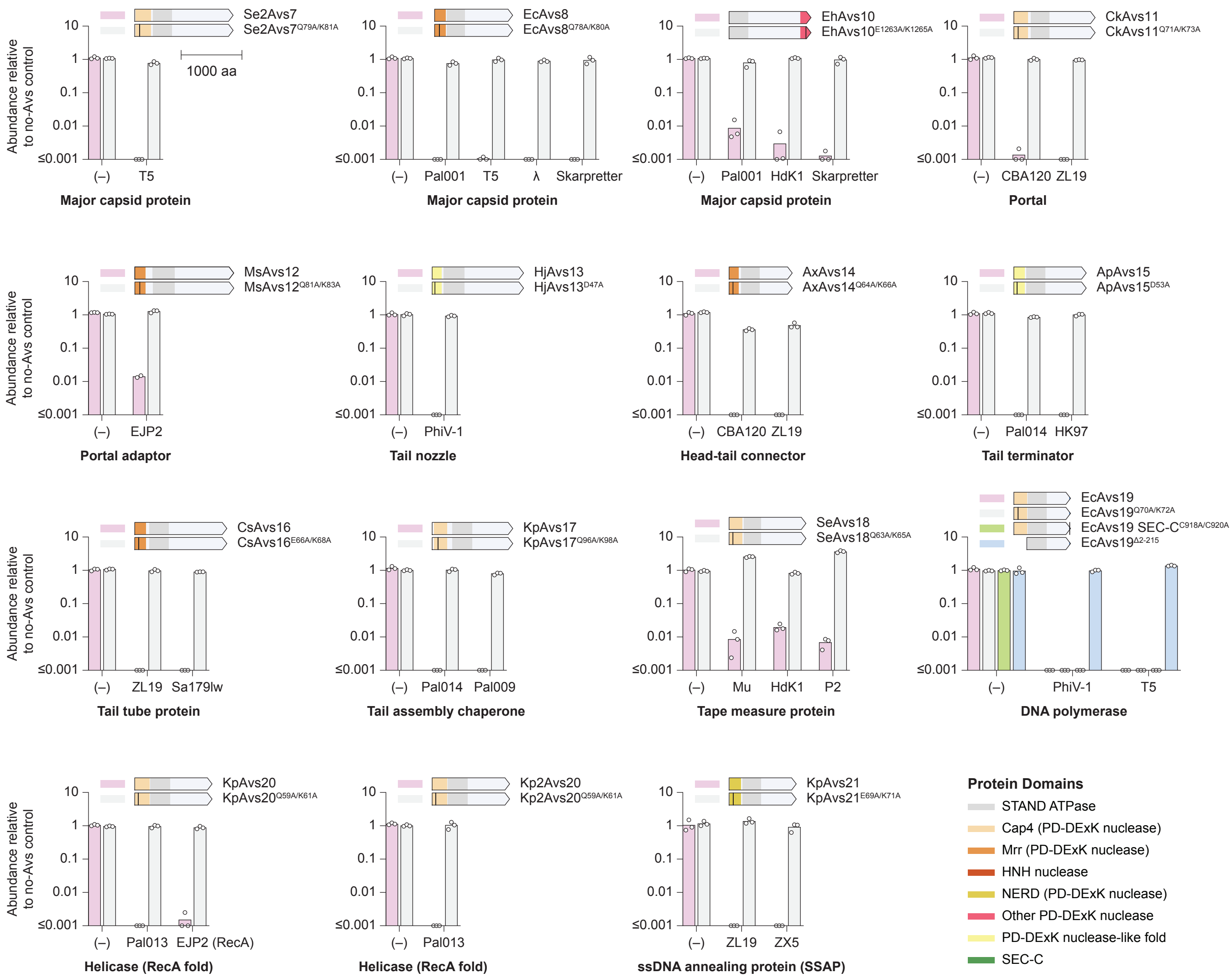

Supplementary Fig. 13 | Avs effector domains are required for cellular toxicity. Toxicity of wild-type Avs and effector domain mutants co-expressed with their cognate phage triggers in *E. coli*.

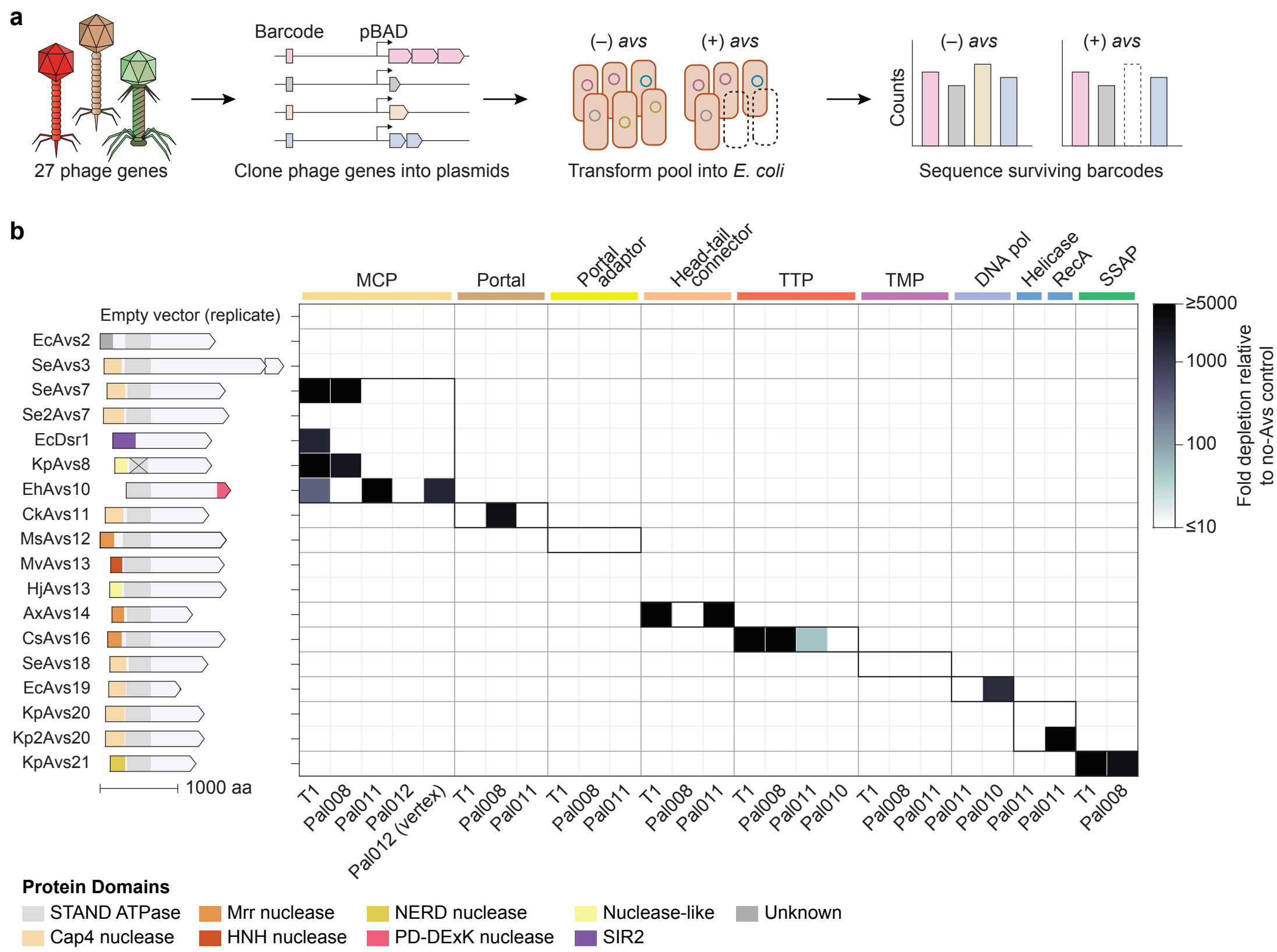

Supplementary Fig. 14 | Expanded co-expression toxicity screen with an additional 27 phage genes.

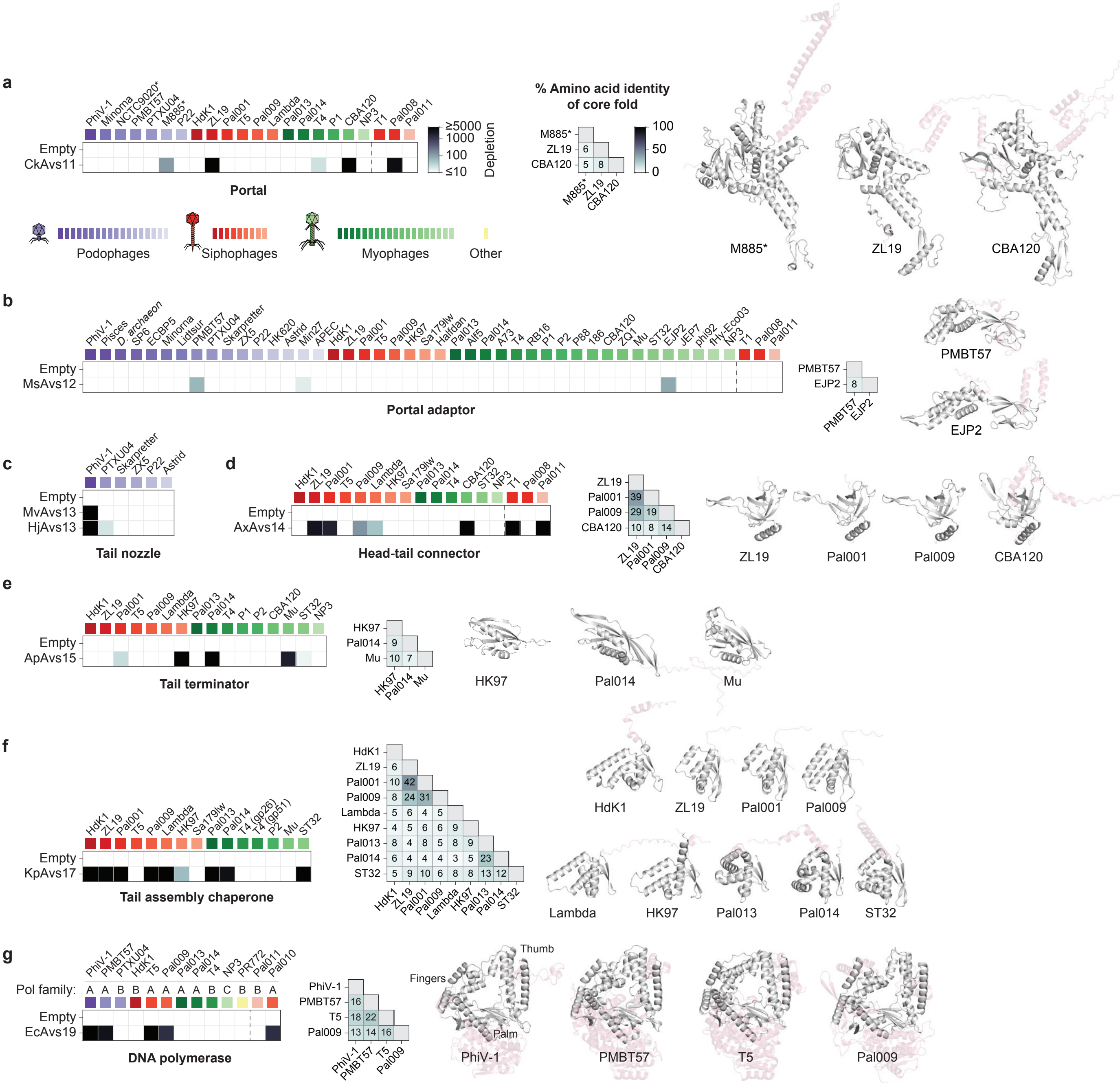

Supplementary Fig. 15 | Avs pattern recognition of conserved core structural folds (related to Fig. 5a). a–f, Toxicity quantification and structural comparisons of AlphaFold3 models for the portal (a), portal adaptor (b), tail nozzle (c), head-tail connector (d), tail terminator (e), tail assembly chaperone (f), and DNA polymerase (g). Toxicity data are replotted from Fig. 5a and Supplementary Fig. 14. Pairwise amino acid sequence identities between representative core folds are indicated; corresponding AlphaFold3 structures highlight these folds in dark gray. Sequence identities below ~5% are considered random. NP3, *Pseudomonas* phage NP3. Asterisks (*) denote prophages.

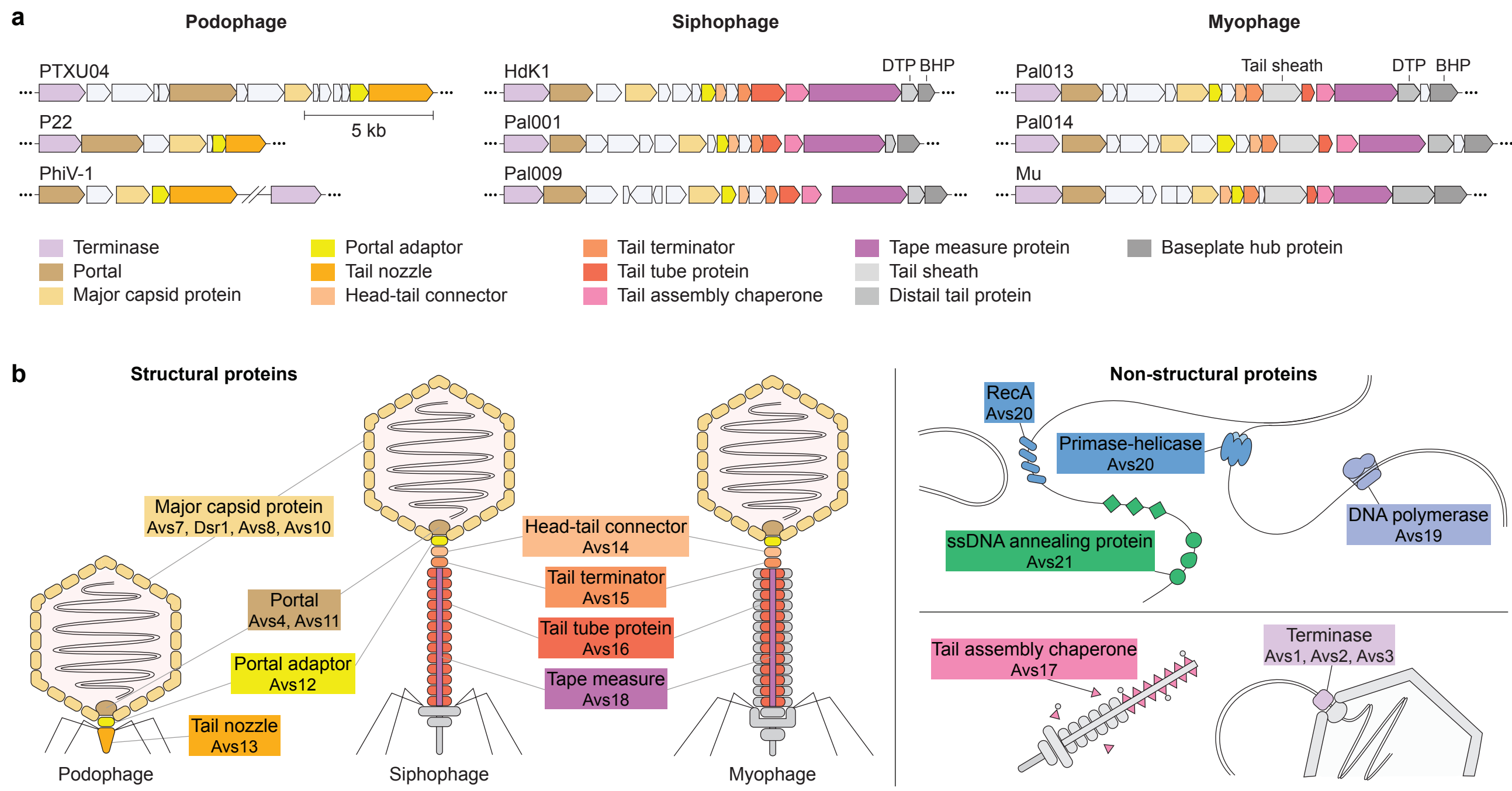

Supplementary Fig. 16 | Avs targets are conserved across diverse tailed phages. a, Genomic context of Avs targets across different representative phages. **b**, Schematic of the functional roles of the structural and replicative phage proteins recognized by distinct Avs families (Fig. 5b). DTP, distal tail protein; BHP, baseplate hub protein.

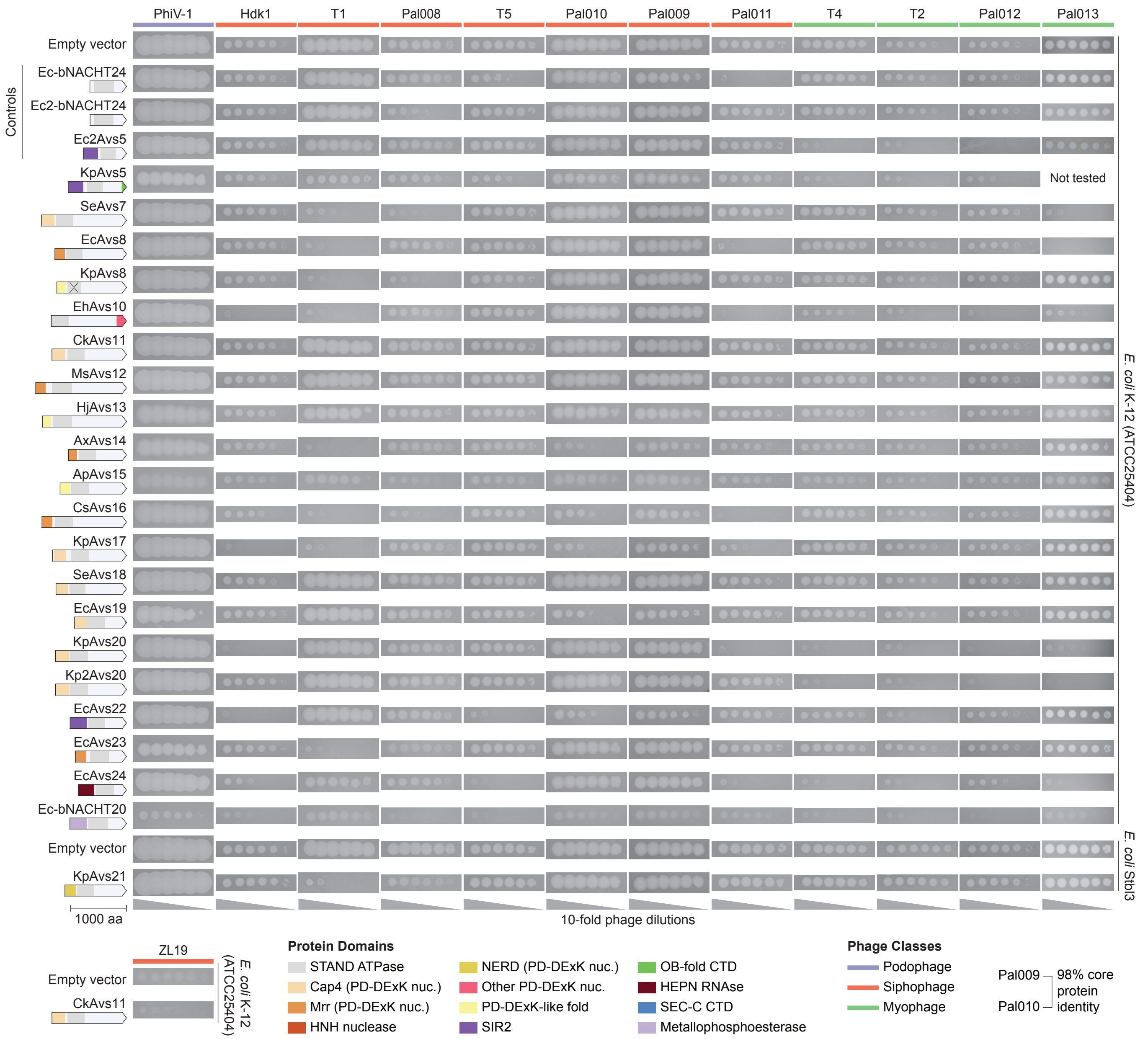

Supplementary Fig. 17 | Anti-phage activity of Avs proteins in *E. coli.* Plaque assays assessing Avs-mediated defense against a panel of 12 coliphages. Spots represent 10-fold serial phage dilutions (left to right). The cross mark on KpAvs8 indicates the natural absence of conserved nucleotide-binding residues in its STAND NTPase domain. Sequence identities for Pal009 and Pal010 are based on core structural and replicative proteins.

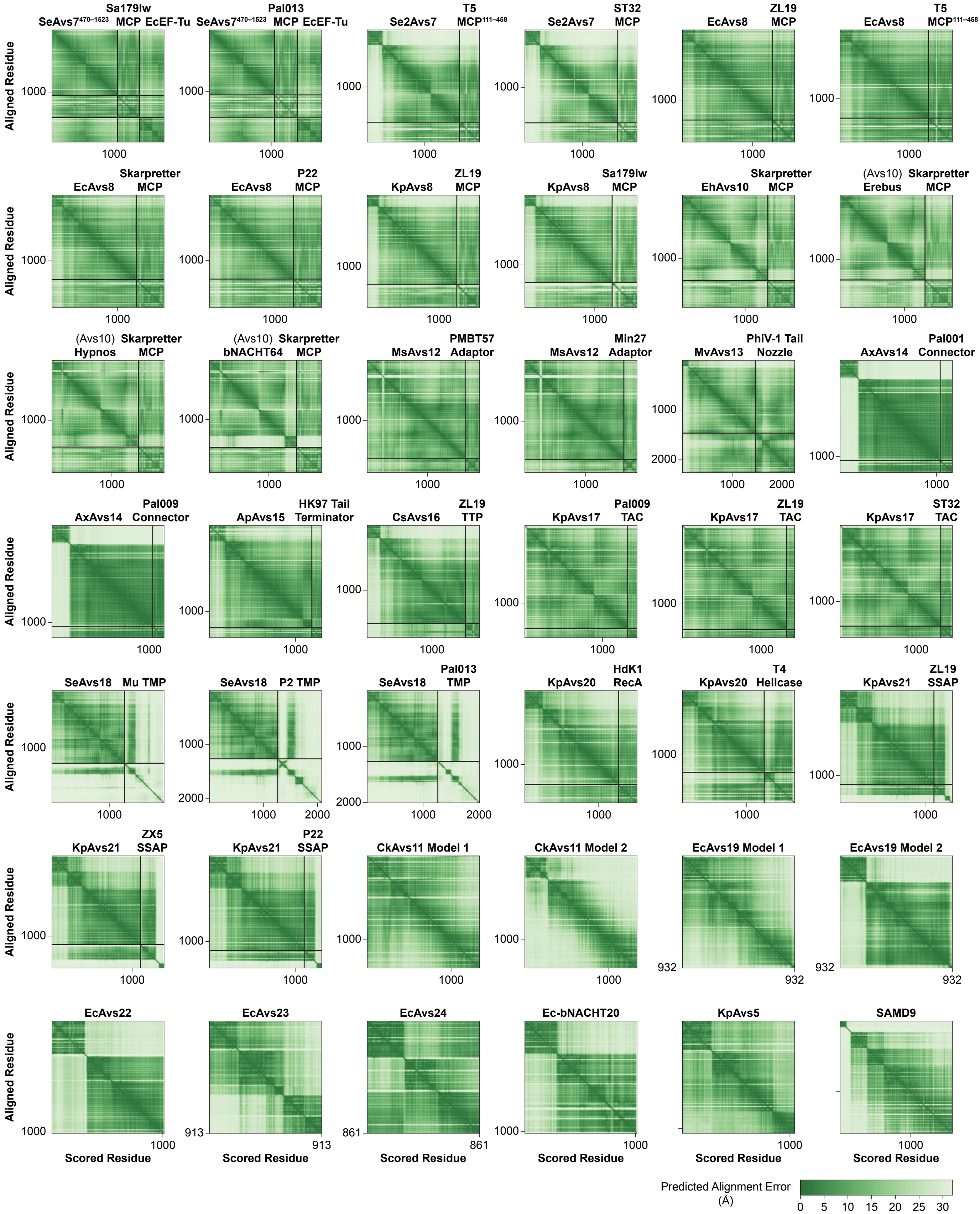

Supplementary Fig. 18 | Quality of AlphaFold structural models. Predicted alignment error (PAE) heatmaps for the AlphaFold2 and AlphaFold3 models presented in Fig. 6 and Extended Data Figs. 10–11.

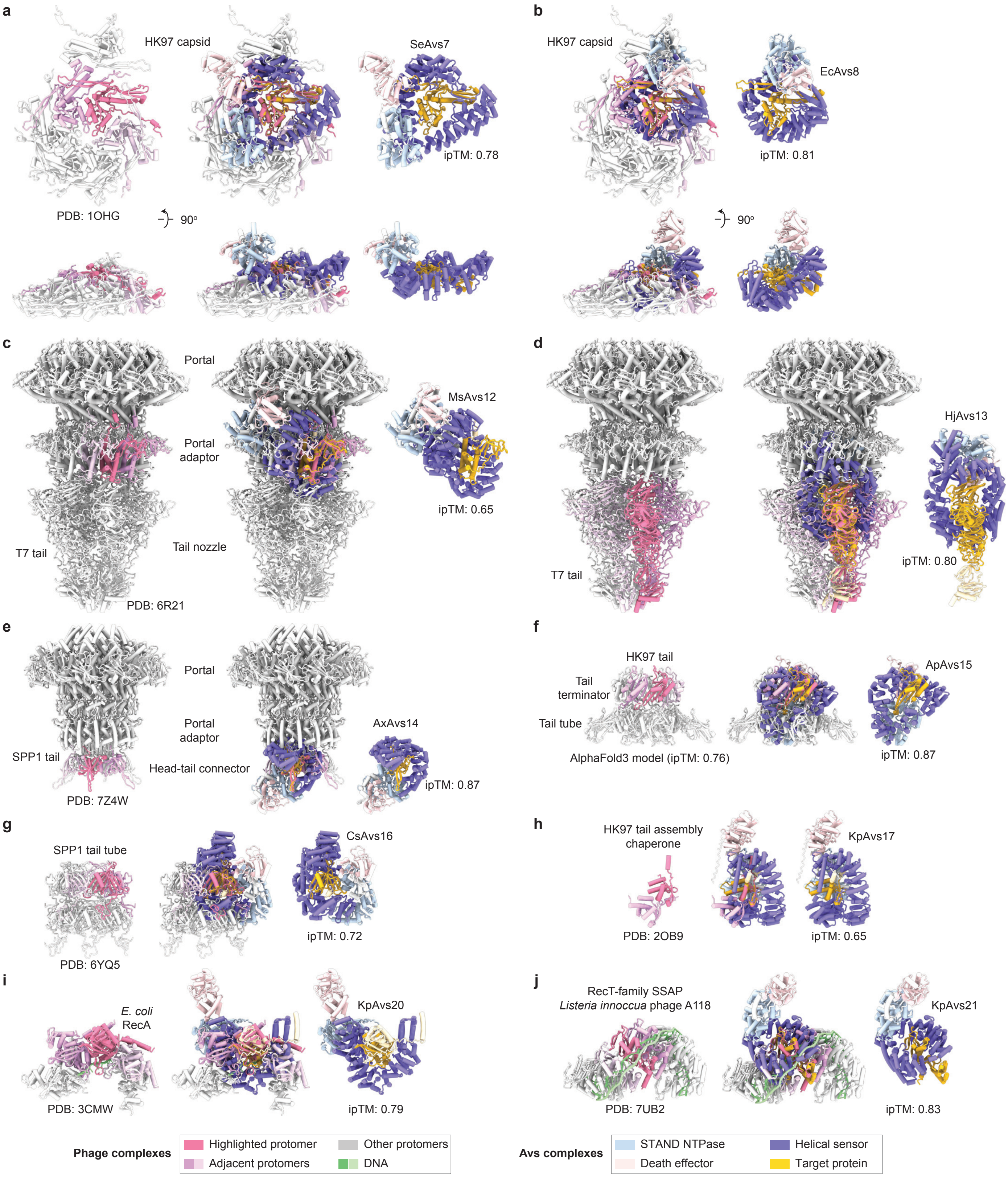

Supplementary Fig. 19 | Avs binding is sterically incompatible with target protein oligomerization. a–j, Structures of the HK97 capsid hexon (**a**, **b**), portal adaptor ring (**c**), complete tail nozzle (**d**), head-tail connector ring (**e**), tail terminator complex (**f**), tail tube (**g**), tail assembly chaperone (**h**), DNA-bound RecA (**i**), and DNA-bound ssDNA-annealing protein (**j**) overlaid with AlphaFold3 models of the respective Avs bound to a target monomer. The tail terminator complex is an AlphaFold3 model; all other target assemblies are experimental structures from the Protein Data Bank. ipTM, interface predicted template modeling score (0.0–1.0).
