# Supplementary material for: Diverse bacterial pattern recognition receptors sense the core phage proteome
Source: Nature. 2026 Jul 29;657(8132):735–43. doi: 10.1038/s41586-026-10852-6 (PMC13581612; doi:10.1038/s41586-026-10852-6)
Supplement: Supplementary file 2 [file 41586_2026_10852_MOESM2_ESM.pdf]

nature portfolio

Corresponding author(s): Alex Gao
Last updated by author(s): June 10, 2026

Reporting Summary

Nature Portfolio wishes to improve the reproducibility of the work that we publish. This form provides structure for consistency and transparency in reporting. For further information on Nature Portfolio policies, see our Editorial Policies and the Editorial Policy Checklist.

Please do not complete any field with "not applicable" or n/a. Refer to the help text for what text to use if an item is not relevant to your study.
For final submission: please carefully check your responses for accuracy; you will not be able to make changes later.

Statistics

For all statistical analyses, confirm that the following items are present in the figure legend, table legend, main text, or Methods section.

n/a	Confirmed	
☐	☒	The exact sample size (*n*) for each experimental group/condition, given as a discrete number and unit of measurement
☐	☒	A statement on whether measurements were taken from distinct samples or whether the same sample was measured repeatedly
☐	☒	The statistical test(s) used AND whether they are one- or two-sided<br>*Only common tests should be described solely by name; describe more complex techniques in the Methods section.*
☒	☐	A description of all covariates tested
☒	☐	A description of any assumptions or corrections, such as tests of normality and adjustment for multiple comparisons
☐	☒	A full description of the statistical parameters including central tendency (e.g. means) or other basic estimates (e.g. regression coefficient) AND variation (e.g. standard deviation) or associated estimates of uncertainty (e.g. confidence intervals)
☐	☒	For null hypothesis testing, the test statistic (e.g. *F*, *t*, *r*) with confidence intervals, effect sizes, degrees of freedom and *P* value noted<br>*Give P values as exact values whenever suitable.*
☒	☐	For Bayesian analysis, information on the choice of priors and Markov chain Monte Carlo settings
☒	☐	For hierarchical and complex designs, identification of the appropriate level for tests and full reporting of outcomes
☒	☐	Estimates of effect sizes (e.g. Cohen's *d*, Pearson's *r*), indicating how they were calculated

Our web collection on statistics for biologists contains articles on many of the points above.

Software and code

Policy information about availability of computer code

Data collection	Nonredundant protein database was downloaded from National Center for Biotechnology Information on February 2022 using update_blastdb.pl of the ncbi-blast+ (2.15.0+). Structural data was acquired using EPU 3.2.
Data analysis	PSI-BLAST (2.15.0+), MUSCLE (v5), FAMSA (v2), FastTree (v2.1.11), MAFFT (v7.520 and v7.525), MMseqs2 (v16.747c6), trimAl (1.2 and 1.5), IQ-TREE (1.6.12 and 2.3.6), HH-suite3 (HHPred; https://github.com/soedinglab/hh-suite), iTOL (https://itol.embl.de), ggtree (v3.10.1), DefenseFinder (v1.2.2), AlphaFold3, Foldseek (version: 8dc75c74ad0eddab73cfd905963d13bf74dc012b), CutAdapt (v1.18), SPAdes (v4.2.0), unicycler (v0.5.1), Prokka (1.14.6), Pharokka (v1.7.0), Phold (v1.0.0), PyGenomeViz (v1.6.1), ImageJ (1.54g), ChimeraX (v1.8), cryoSPARC (v4.4), PyMOL (v2.5.7 and v3.0.3), MotionCor2 (v1.2.3), EMAN2 (v2.91), ColabFold (v1.5.2; https://colab.research.google.com/github/sokrypton/ColabFold/blob/main/AlphaFold2.ipynb), PHASTEST (Docker image wishartlab/phastest-docker-single, ID 2ce737d970ab), Masspike (Gygi lab), Chimera (1.17.3), Coot (0.9.8.93), Phenix (phenix-1.19.2), MolProbity (4.4), PROMALS3D (http://prodata.swmed.edu/promals3d/promals3d.php)

For manuscripts utilizing custom algorithms or software that are central to the research but not yet described in published literature, software must be made available to editors and reviewers. We strongly encourage code deposition in a community repository (e.g. GitHub). See the Nature Portfolio guidelines for submitting code & software for further information.

Data

Policy information about availability of data

All manuscripts must include a data availability statement. This statement should provide the following information, where applicable:

- Accession codes, unique identifiers, or web links for publicly available datasets
- A description of any restrictions on data availability
- For clinical datasets or third party data, please ensure that the statement adheres to our policy

The genome sequences of phages Pal001–013 have been deposited in GenBank under the accession numbers PX985120–PX985132. Protein sequences and taxonomic information are publicly available from the NCBI Protein (https://www.ncbi.nlm.nih.gov/protein/) and Taxonomy (https://www.ncbi.nlm.nih.gov/taxonomy) databases. Mass spectrometric data have been deposited in Proteomics Identification Database under accession code PXD079457. Cryo-EM maps have been deposited in the Electron Microscopy Data Bank under accession numbers EMD-73001 (Map F; composite map of the SeAvs7–ZL19 MCP–EcEF-Tu tetrameric complex), EMD-73002 (Map B), EMD-73003 (Map A), EMD-73004 (Map E), EMD-73005 (Map D), EMD-73006 (Map C), and EMD-48771 (Apo SeAvs7), and EMD-48772 (Map G; SeAvs7–ZL19 MCP–EcEF-Tu monomeric complex). The corresponding atomic models have been deposited in the Protein Data Bank (PDB) under accession codes 9YIX (Map F), 9N01 (Map G) and 9N00 (Apo SeAvs7). Previously published structures rendered in this study are publicly available in the PDB, with accession codes provided in the corresponding figures. Source data for Fig. 2d, 3f, 5a, 6f, j–o, Extended Data Fig. 3d, g, 8, Supplementary Fig. 7–8, 13–14 are provided with this manuscript.

Research involving human participants, their data, or biological material

Policy information about studies with human participants or human data. See also policy information about sex, gender (identity/presentation), and sexual orientation and race, ethnicity and racism.

Reporting on sex and gender	NA
Reporting on race, ethnicity, or other socially relevant groupings	NA
Population characteristics	NA
Recruitment	NA
Ethics oversight	NA

Note that full information on the approval of the study protocol must also be provided in the manuscript.

Field-specific reporting

Please select the one below that is the best fit for your research. If you are not sure, read the appropriate sections before making your selection.

☒ Life sciences ☐ Behavioural & social sciences ☐ Ecological, evolutionary & environmental sciences

For a reference copy of the document with all sections, see nature.com/documents/nr-reporting-summary-flat.pdf

Life sciences study design

All studies must disclose on these points even when the disclosure is negative.

Sample size	No statistical methods were used to predetermine sample size. Each experiment was performed in two or three independent replicates (n = 2 or 3), in line with standard practice for these assays, establishing reproducibility. For cryo-EM, the number of particles in each reconstruction (78,090–207,066 final particles) was sufficient to reach the reported resolutions (2.72–3.43 Å; FSC = 0.143) and to permit reliable model building and refinement, as reflected in the validation statistics in Extended Data Table 1.
Data exclusions	Cryo-EM data processing involved the removal of poor-quality or damaged particles through the standard data classification procedures, as is common and accepted practice in the field.
Replication	All biochemical and genetic experiments were repeated at least three times in independent experiments, with reliable results.
Randomization	Randomization was not relevant to the experiments in our study since no grouping was involved.
Blinding	Blinding was not relevant to the experiments in our study since no subjective allocation was involved.

Reporting for specific materials, systems and methods

We require information from authors about some types of materials, experimental systems and methods used in many studies. Here, indicate whether each material, system or method listed is relevant to your study. If you are not sure if a list item applies to your research, read the appropriate section before selecting a response.

Materials & experimental systems

n/a	Involved in the study
☒	☐ Antibodies
☒	☐ Eukaryotic cell lines
☒	☐ Palaeontology and archaeology
☒	☐ Animals and other organisms
☒	☐ Clinical data
☒	☐ Dual use research of concern
☒	☐ Plants

Methods

n/a	Involved in the study
☒	☐ ChIP-seq
☒	☐ Flow cytometry
☒	☐ MRI-based neuroimaging

Plants

Seed stocks	NA
Novel plant genotypes	NA
Authentication	NA

This checklist template is licensed under a Creative Commons Attribution 4.0 International License, which permits use, sharing, adaptation, distribution and reproduction in any medium or format, as long as you give appropriate credit to the original author(s) and the source, provide a link to the Creative Commons license, and indicate if changes were made. The images or other third party material in this article are included in the article's Creative Commons license, unless indicated otherwise in a credit line to the material. If material is not included in the article's Creative Commons license and your intended use is not permitted by statutory regulation or exceeds the permitted use, you will need to obtain permission directly from the copyright holder. To view a copy of this license, visit http://creativecommons.org/licenses/by/4.0/
